# Supplementary material for: Sampling errors and variability in video transects for assessment of reef fish assemblage structure and diversity
Source: PLoS One. 2022 Jul 25;17(7):e0271043. doi: 10.1371/journal.pone.0271043 (PMC9312474; doi:10.1371/journal.pone.0271043)
Supplement: S1 File — (PDF) [file pone.0271043.s001.pdf]

S1. Instantaneous fish displacement

As fish displacement is not necessarily random, even at very short time scales, we prefer to use the term instantaneous fish displacement, instead of random fish displacement (Irigoyen et al., 2013; MacNeil et al., 2008), throughout the article to include all fish displacement that would take place under natural conditions at very short time scales. While the instantaneous displacement causes actual variation in the local (i.e. within sampling unit) abundance, the random counting/detection errors introduce false variation in local abundance.
