# Supplementary material for: Sampling errors and variability in video transects for assessment of reef fish assemblage structure and diversity
Source: PLoS One. 2022 Jul 25;17(7):e0271043. doi: 10.1371/journal.pone.0271043 (PMC9312474; doi:10.1371/journal.pone.0271043)
Supplement: S2 File — (PDF) [file pone.0271043.s002.pdf]

S2. Applications of video monitoring

Video material has been used for different purposes at different times after the video material was collected. It has been used to characterize physical habitats (Cappo et al., 2003), to obtain biomass estimates (Harvey et al., 2001), to study fish behavior (Watson and Harvey, 2007), and to train algorithms to pick out fish and identify them automatically (Boom et al., 2012). In addition, because the collecting and analyzing of the video material can be done by two different persons, people trained in underwater video collection do not necessarily have to be trained in identifying and counting fish.
