# Supplementary material for: Sampling errors and variability in video transects for assessment of reef fish assemblage structure and diversity
Source: PLoS One. 2022 Jul 25;17(7):e0271043. doi: 10.1371/journal.pone.0271043 (PMC9312474; doi:10.1371/journal.pone.0271043)
Supplement: S3 File — (PDF) [file pone.0271043.s003.pdf]

S3. Video analysis

Fish were counted in such a way that both the MinCount and MaxCount of each observation could be determined. For example, if two individuals of the same species were recorded in $frame_1$ and a few moments later a third individual of the same species entered the field of view (FOV) of $frame_2$, while the other two individuals were still present in the FOV, than only the recording of $frame_2$ would be included. However, if the two individuals encountered in $frame_1$ moved away before the individual of $frame_2$ moved within the FOV, then both recordings were included separately as the third individual may actually have been one of the first two individuals. An easier way to determine MinCount would have been to only note down the maximum number of individuals. For example, if first two individuals would be recorded then the video analyst would write down two. If then three individuals would be recorded the video analyst would change that number to three. If less than three individuals would then be recorded this would not be included. For the MaxCount, the first method was actually the most efficient one and since MaxCount is typically used for video transects (Mallet and Pelletier, 2014; Wartenberg and Booth, 2015), this method was chosen.
