# Supplementary material for: Sampling errors and variability in video transects for assessment of reef fish assemblage structure and diversity
Source: PLoS One. 2022 Jul 25;17(7):e0271043. doi: 10.1371/journal.pone.0271043 (PMC9312474; doi:10.1371/journal.pone.0271043)
Supplement: S4 File — (PDF) [file pone.0271043.s004.pdf]

S4. Indicator species

Applied ecological studies often assess the potential of different species to serve as indicators for water quality. For each island, two potential indicator species were determined using CAP analysis based on the grouping factor Location. The species with the highest Spearman correlation to the CAP axes (m=4) were retained (Table S3), as they are representative for observed differences among groups. The full 50 meter dataset was split up for the two islands and separate CAP analyses were performed. Data was fourth-root transformed and Bray-Curtis dissimilarities were calculated. We decided to perform one CAP analysis per island as the differences in species between the islands may be more related to biogeography and dispersal limitations than to environmental conditions which dictate the water quality. The Spinster Wrasse (*Halichoeres nicholsi*) and Sabertooth Blenny (*Plagiotremus azaleus*) were selected for Santa Cruz, while the Bravo Clinid (*Gobioclinus dendriticus*) and Panamic Fanged Blenny (*Ophioblennius steindachneri*) were selected for Floreana. It should be noted that these species might not be actual indicator species for water quality, as the constrained ordination was performed on an artificial grouping factor, rather than on actual environmental gradients.
