# Supplementary material for: Sampling errors and variability in video transects for assessment of reef fish assemblage structure and diversity
Source: PLoS One. 2022 Jul 25;17(7):e0271043. doi: 10.1371/journal.pone.0271043 (PMC9312474; doi:10.1371/journal.pone.0271043)
Supplement: S1 Fig — (PDF) [file pone.0271043.s005.pdf]

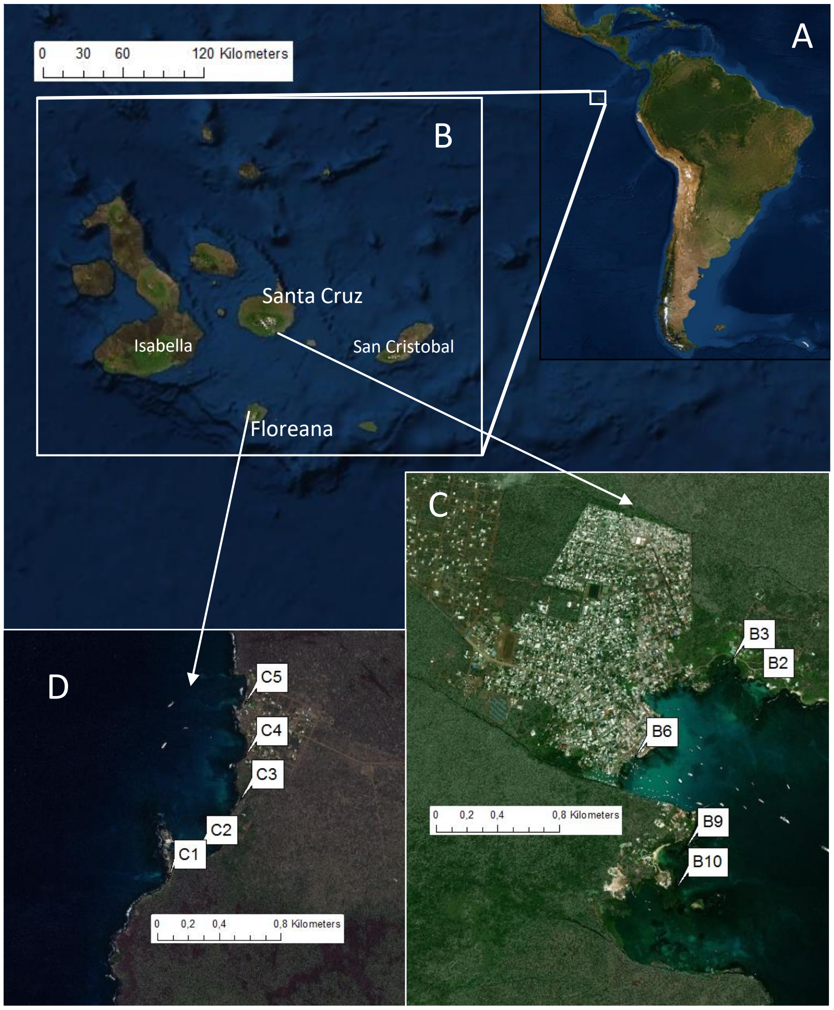

Figure S1: Map of the study area. (**A**) South-American continent with depiction of the Galapagos archipelago. (**B**) Galapagos archipelago with depiction of the two studied islands. (**C**) The city Puerto Ayora on Santa Cruz island with indication of the study locations. (**D**) The city Puerto Velazco Ibarra on Floreana island with indication of the study locations. Landsat 8 imagery was used to construct the maps.
