# Supplementary material for: Sampling errors and variability in video transects for assessment of reef fish assemblage structure and diversity
Source: PLoS One. 2022 Jul 25;17(7):e0271043. doi: 10.1371/journal.pone.0271043 (PMC9312474; doi:10.1371/journal.pone.0271043)
Supplement: S3 Fig — (PDF) [file pone.0271043.s007.pdf]

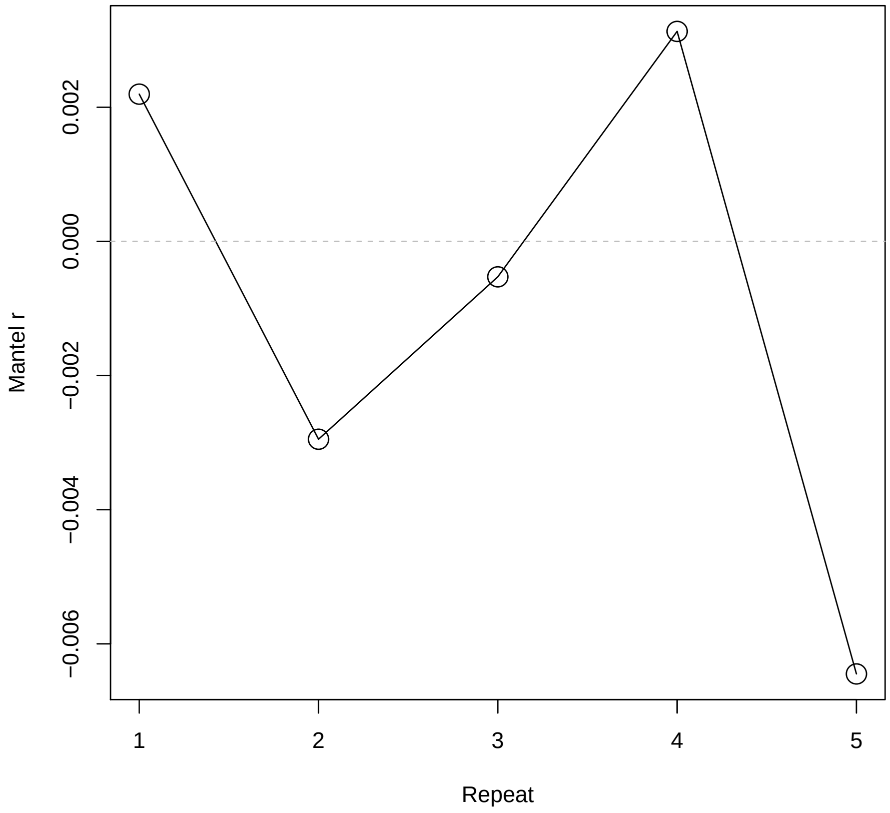

Figure S3: Partial mantel correlogram for temporal auto-correlation of the observed structure of fish assemblages. The effect of the sampling units and observers themselves was partialled out. No significant temporal auto-correlations were found.
