# Supplementary material for: Sampling errors and variability in video transects for assessment of reef fish assemblage structure and diversity
Source: PLoS One. 2022 Jul 25;17(7):e0271043. doi: 10.1371/journal.pone.0271043 (PMC9312474; doi:10.1371/journal.pone.0271043)
Supplement: S5 Fig — (PDF) [file pone.0271043.s009.pdf]

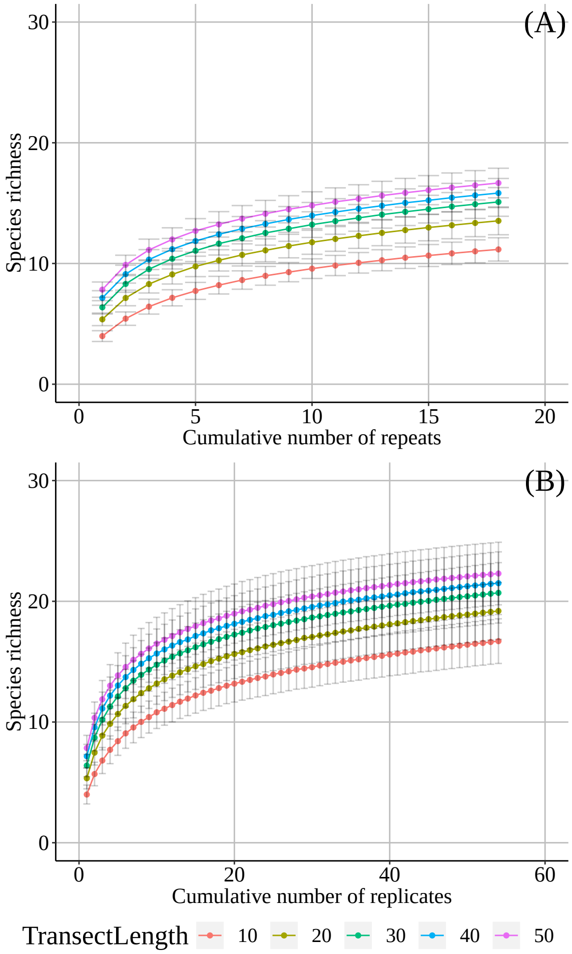

Figure S5: Species Accumulation Curves (SACs) depicting the cumulative species richness in function of the cumulative number of repeats for different transect lengths. Permutations or randomizations without replacement (n=10^4) of the repeats were used to determine the mean and standard deviation. The error bars represent the 95% confidence interval. In (A) the repeats were pooled within Transect (n=18). In (B) the repeats were pooled within Location (n=54).
