# Supplementary material for: Sampling errors and variability in video transects for assessment of reef fish assemblage structure and diversity
Source: PLoS One. 2022 Jul 25;17(7):e0271043. doi: 10.1371/journal.pone.0271043 (PMC9312474; doi:10.1371/journal.pone.0271043)
Supplement: S6 Fig — (PDF) [file pone.0271043.s010.pdf]

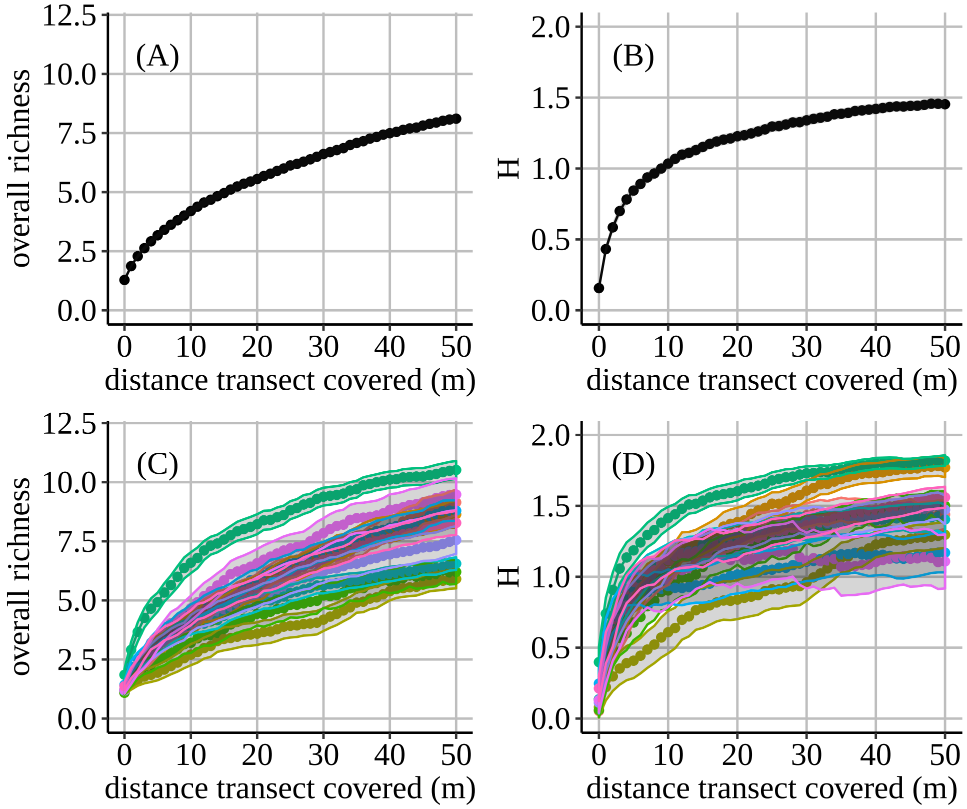

Figure S6: Species Accumulation Curves (SACs) depicting the cumulative species density and cumulative Shannon diversity (H) in function of the transect distance. The error bars represent the 95% confidence interval. In (A) and (B) the average cumulative species density and Shannon diversity of all observations (n=540) are depicted respectively. In (C) and (D) a distinction is made between the 10 locations (n=54).
