# Supplementary material for: Sampling errors and variability in video transects for assessment of reef fish assemblage structure and diversity
Source: PLoS One. 2022 Jul 25;17(7):e0271043. doi: 10.1371/journal.pone.0271043 (PMC9312474; doi:10.1371/journal.pone.0271043)
Supplement: S7 Fig — (PDF) [file pone.0271043.s011.pdf]

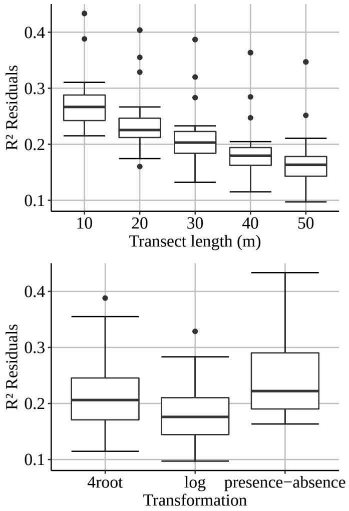

Figure S7: R^2 of the residuals of the PERMANOVA models with Island as fixed factor, Location and Transect as nested random factors and Observer as crossed random factor. $R^2_{Residuals}$ is defined here as the ratio of the sum of squares of the error over the total sum of squares. Different parameters were assessed including the transect length (10, 20, 30, 40 and 50 meters), metric (MaxCount and MinCount), transformation (4root, logarithm and presence-absence) and the method to calculate the dissimilarity matrix (Bray-Curtis, Euclidean, Gower and Kulczynski).
