# Supplementary material for: Sampling errors and variability in video transects for assessment of reef fish assemblage structure and diversity
Source: PLoS One. 2022 Jul 25;17(7):e0271043. doi: 10.1371/journal.pone.0271043 (PMC9312474; doi:10.1371/journal.pone.0271043)
Supplement: S8 Fig — (PDF) [file pone.0271043.s012.pdf]

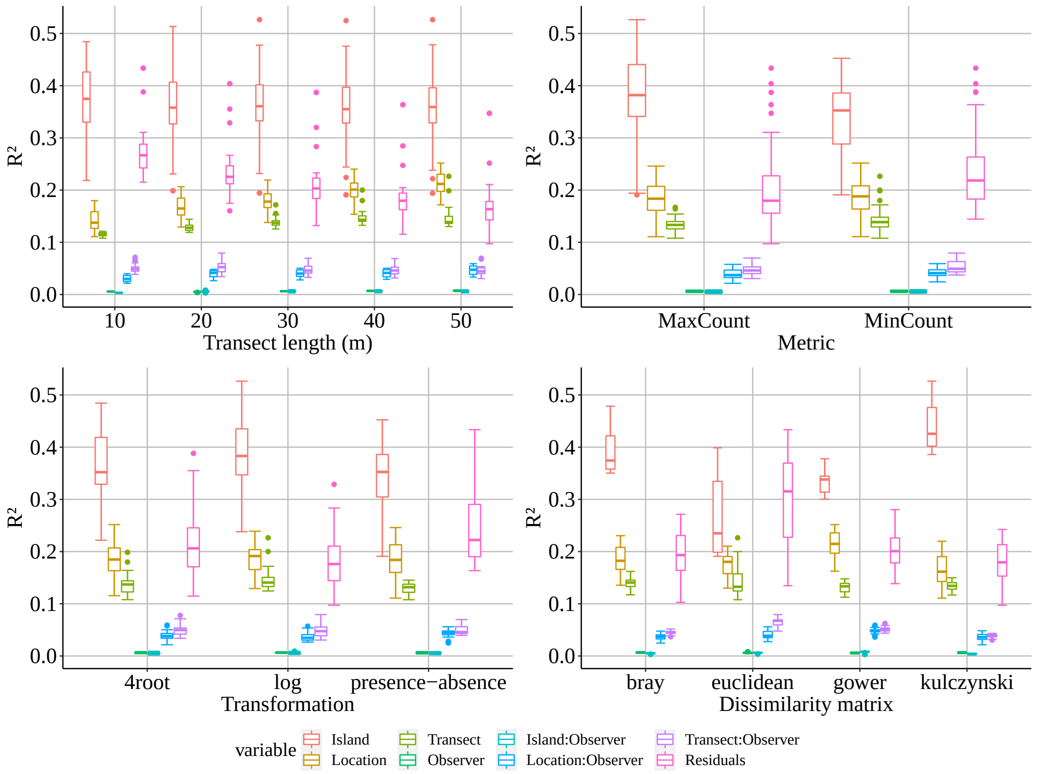

Figure S8: R^2 of the different factors of the PERMANOVA models with Island as fixed factor, Location and Transect as nested random factors and Observer and its interactions with the other factors as crossed random factors. R^2 is defined here as the ratio of the sum of squares over the total sum of squares. Different parameters were assessed including the transect length (10, 20, 30, 40 and 50 meters), metric (MaxCount and MinCount), transformation (4root, logarithm and presence absence data) and the method to calculate the dissimilarity matrix (Bray-Curtis, Euclidean, Gower and Kulczynski).
