# Supplementary material for: Sampling errors and variability in video transects for assessment of reef fish assemblage structure and diversity
Source: PLoS One. 2022 Jul 25;17(7):e0271043. doi: 10.1371/journal.pone.0271043 (PMC9312474; doi:10.1371/journal.pone.0271043)
Supplement: S9 Fig — (PDF) [file pone.0271043.s013.pdf]

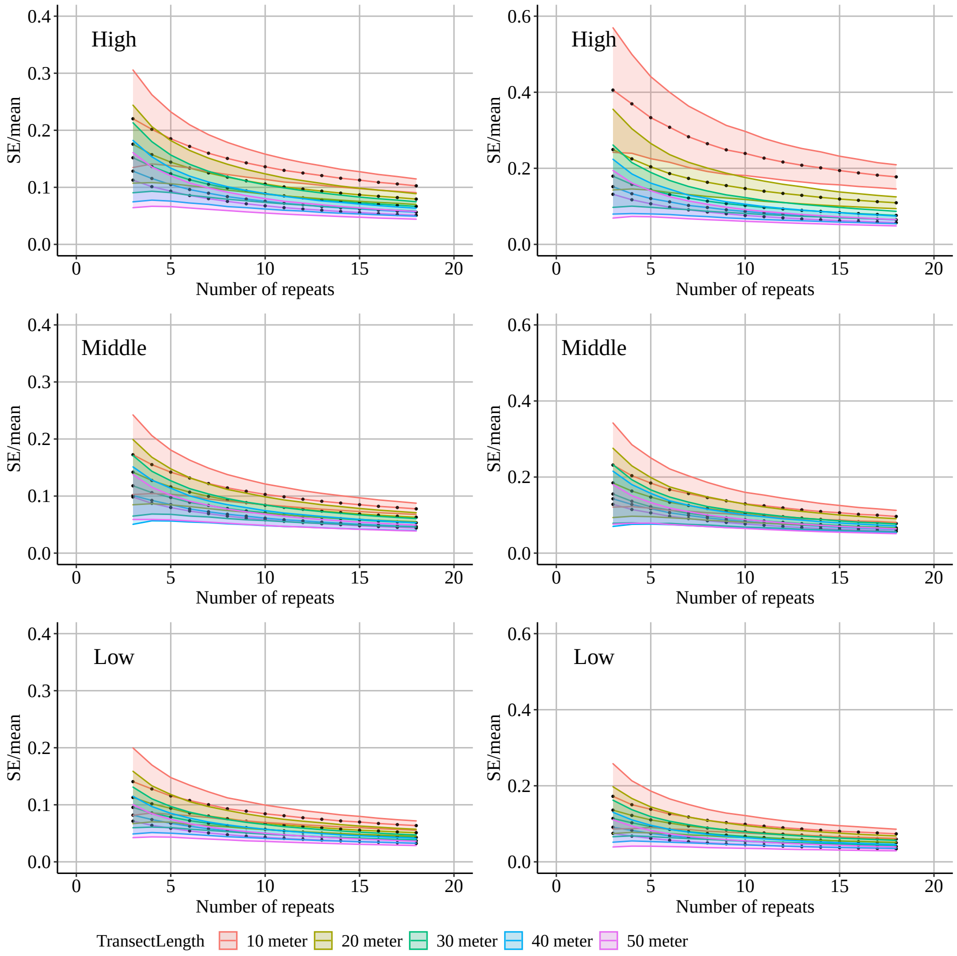

Figure S9: Precision estimates of species density (left) and Shannon diversity (right) with precision defined as the inverse of the standard error over the mean. The 30 transects were divided in 3 equally sized groups with different levels of turbidity (High, Middle and Low). Different transect lengths were considered ranging from 10, 20, 30, 40 to 50 meters. Monte Carlo simulations (n=10^4) were applied to determine the precision per transect. The average precision over all transects is visualized. The error bars represent the 95% confidence intervals which were constructed using the pooled standard deviation of the estimates.
