# Supplementary material for: Sampling errors and variability in video transects for assessment of reef fish assemblage structure and diversity
Source: PLoS One. 2022 Jul 25;17(7):e0271043. doi: 10.1371/journal.pone.0271043 (PMC9312474; doi:10.1371/journal.pone.0271043)
Supplement: S10 Fig — (PDF) [file pone.0271043.s014.pdf]

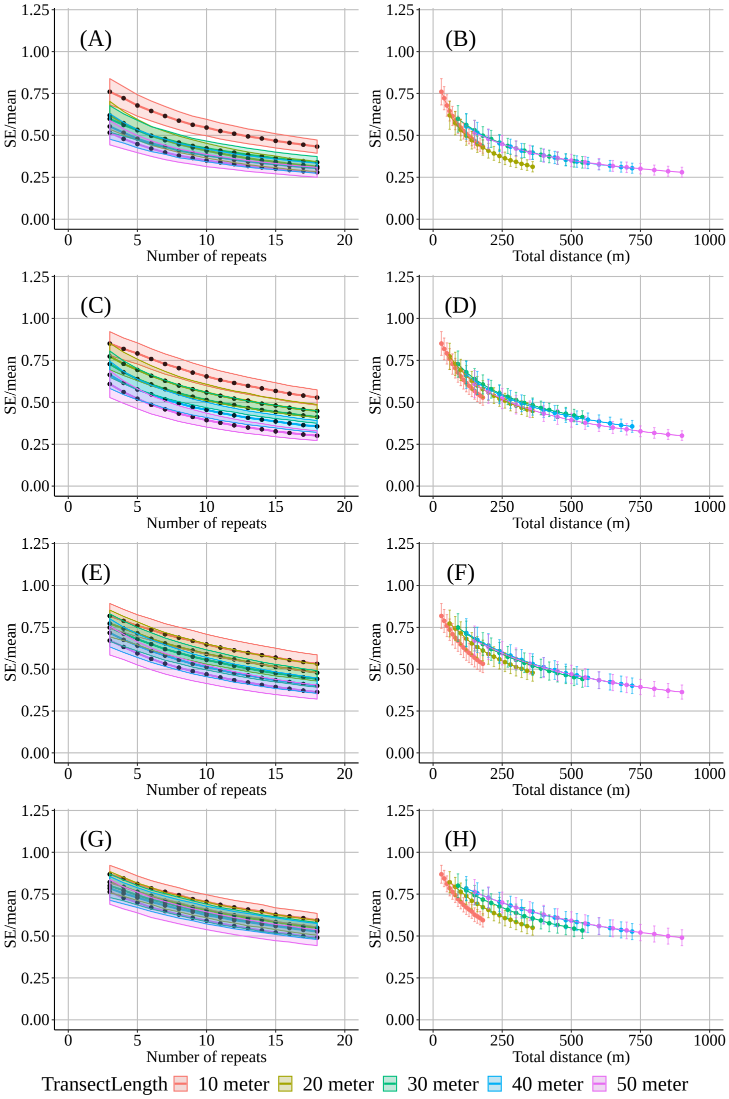


Figure S10: Precision estimates of abundances (N) of different potential indicator species in function of the number of repeats (A,C,E,G) and in function of the total swim distance (B,D,F,H). Precision is defined as the inverse of the standard error over the mean. Total swim distance is defined as the number of repeats multiplied with the transect length. The potential indicator species were Spinster Wrasse (*Halichoeres nicholsi*); A, B, Sabertooth Blenny (*Plagiotremus azaleus*); C, D, Bravo Clinid (*Gobioclinus dendriticus*); E, F, and Panamic Fanged Blenny (*Ophioblennius steindachneri*)); G, H. Different transect lengths were considered ranging from 10, 20, 30, 40 to 50 meters. Monte Carlo simulations ($n=10^4$) were applied to determine the precision per transect. The average precision over all transects is visualized. The error bars represent the 95% confidence intervals which were constructed using the pooled standard deviation of the estimates.
