# Supplementary material for: Sampling errors and variability in video transects for assessment of reef fish assemblage structure and diversity
Source: PLoS One. 2022 Jul 25;17(7):e0271043. doi: 10.1371/journal.pone.0271043 (PMC9312474; doi:10.1371/journal.pone.0271043)
Supplement: S1 Table — (PDF) [file pone.0271043.s015.pdf]

Species	lag 0	lag 1	lag 2	lag 3	lag 4	lag 5	lag 6	lag 7	lag 8
Amarillo snapper (*Lutjanus argentiventris*)	1	-0.03	-0.05	-0.02	-0.11	0	-0.03	-0.1	-0.01
Balloon fish (*Diodon holocanthus*)	1	-0.06	-0.07	-0.07	-0.07	-0.08	-0.08	-0.08	-0.03
Banded wrasse (*Halichoeres notospilus*)	1	0.2	0.16	0.03	-0.03	-0.07	-0.13	-0.15	-0.07
Black striped salema (*Xenocys jessiae*)	1	-0.01	0.03	0.02	0.03	-0.01	-0.04	-0.09	-0.05
Blacktip cardinalfish (*Apogon atradorsatus*)	1	0.06	0.14	-0.03	0.04	-0.08	-0.09	-0.13	-0.11
Blue and gold snapper (*Lutjanus viridis*)	1	0.06	0.07	-0.09	-0.11	-0.09	-0.1	-0.03	-0.04
Blue chin parrotfish (*Scarus ghobban*)	1	0.02	0.01	0.04	0.02	-0.07	-0.07	-0.03	-0.04
Bravo clinid (*Gobioclinus dendriticus*)	1	0.16	0.11	0.01	0.02	-0.03	-0.08	-0.1	-0.14
Bullseye puffer (*Sphoeroides annulatus*)	1	0.09	-0.05	-0.07	0.01	-0.04	-0.1	-0.11	-0.06
Chameleon wrasse (*Halichoeres dispilus*)	1	0.29	0.18	0	0	-0.03	-0.09	-0.09	-0.09
Cortez rainbow wrasse (*Thalassoma lucasanum*)	1	0.13	0.17	0	-0.01	-0.07	-0.13	-0.1	-0.06
Eagle ray (*Aetobatus narinari*)	1	-0.06	-0.07	-0.07	-0.07	-0.02	-0.02	-0.02	-0.03
Flag cabrilla (*Epinephelus labriformis*)	1	0.04	0.01	-0.04	-0.07	-0.06	-0.07	-0.03	-0.04
Galapagos bullhead shark (*Heterodontus quoyi*)	1	0	-0.01	-0.01	-0.01	-0.02	-0.02	-0.02	-0.03
Galapagos grunt (*Orthopristis forbesi*)	1	0.21	0.03	0.01	0.09	-0.01	-0.17	-0.11	-0.07
Galapagos ringtail damselfish (*Stegastes beebei*)	1	0.26	0.22	0.04	0.03	-0.11	-0.12	-0.11	-0.15
Galapagos Seabream (*Archosargus pourtalesi*)	1	0.27	-0.05	0	0.09	-0.06	-0.18	-0.04	-0.08
Galapagos shark (*Carcharhinus galapagensis*)	1	-0.06	-0.05	-0.05	-0.03	-0.02	-0.02	-0.02	-0.03
Galapagos sheepshead wrasse (*Semicossyphus darwini*)	1	-0.06	-0.07	-0.07	-0.07	-0.08	-0.02	-0.02	-0.03
Galapagos triplefin blenny (*Lepidonectes corallicola*)	1	0.16	-0.03	-0.01	-0.04	-0.04	-0.08	-0.07	-0.03
Giant hawkfish (*Cirrhitus rivulatus*)	1	-0.02	0.13	-0.03	-0.07	-0.07	-0.14	-0.05	-0.1
Harlequin wrasse (*Bodianus eclancheri*)	1	0.73*	0.47*	0.2	-0.06	-0.08	-0.1	-0.11	-0.13
Jewel moray (*Muraena lentiginosa*)	1	-0.06	-0.06	-0.06	0.13	-0.05	-0.06	-0.05	-0.05
King angelfish (*Holacanthus passer*)	1	0.06	-0.02	-0.02	-0.01	-0.05	-0.07	-0.06	-0.06
Loosetooth parrotfish (*Nicholsina denticulata*)	1	-0.06	-0.07	-0.07	-0.07	-0.02	-0.02	-0.02	-0.03
Marbled goby (*Gobio manchada*)	1	0.17	0.15	0.04	0.02	-0.06	-0.11	-0.12	-0.12
Marbled ray (*Taeniurops meyeni*)	1	-0.06	-0.07	-0.07	-0.01	-0.02	-0.02	-0.02	-0.03
Mexican hogfish (*Bodianus diplotaenia*)	1	0.11	0.07	0.04	0.05	-0.07	-0.04	-0.07	-0.08
Mojarra grunt (*Haemulon scudderii*)	1	0	0.03	0	0.01	-0.05	-0.04	-0.04	-0.05
Mullet snapper (*Lutjanus aratus*)	1	0.42	-0.08	-0.02	-0.03	-0.03	-0.04	-0.05	-0.06
Pacific dog snapper (*Lutjanus novemfasciatus*)	1	0.46*	0.2	0.29	0.32	0.14	-0.13	-0.15	-0.17
Pacific spotfin mojarra (*Eucinostomus dowii*)	1	0.14	0.09	0.07	0.03	0.01	0.02	-0.11	-0.04
Panamic fanged blenny (*Ophioblennius steindachneri*)	1	0	0	-0.07	0	-0.09	-0.07	-0.04	-0.03
Panamic sergeant major (*Abudefduf troschelii*)	1	0.37	0.19	0.07	0.04	-0.06	-0.1	-0.14	-0.08
Razor surgeonfish (*Prionurus laticlavius*)	1	0.06	0.16	-0.09	-0.1	-0.07	-0.15	-0.06	-0.05
Reef cornetfish (*Fistularia commersonii*)	1	0.29	0.06	-0.04	-0.03	-0.02	-0.09	-0.08	-0.08
Sabertooth blenny (*Plagiotremus azaleus*)	1	0.12	0.08	0.01	-0.04	-0.11	-0.12	-0.13	-0.07
Spinster wrasse (*Halichoeres nicholsi*)	1	0.11	0.12	0.02	-0.03	-0.06	-0.06	-0.04	-0.05
Spotted cabrilla (*Epinephelus analogus*)	1	0	-0.01	-0.01	-0.01	-0.02	-0.02	-0.02	-0.03
Stone scorpionfish (*Scorpaena mystes*)	1	0.27	0.08	0.04	-0.02	-0.08	-0.07	-0.08	-0.08
Striped mullet (*Mugil cephalus*)	1	-0.06	-0.01	-0.01	-0.01	-0.02	-0.02	-0.02	-0.03
Three banded butterfly fish (*Chaetodon humeralis*)	1	0.07	0.05	0.04	-0.04	-0.13	0.04	-0.04	-0.07
Throat-spotted blenny (*Malacoctenus tetranemus*)	1	0.29	-0.07	-0.06	-0.06	-0.07	-0.08	-0.08	-0.09
Tiger snake eel (*Myrichthys maculosus*)	1	0.04	-0.07	-0.08	-0.01	-0.04	-0.05	-0.13	-0.09
Triplefin blenny (*Lepidonectes corallicola*)	1	-0.06	-0.01	-0.01	-0.01	-0.02	-0.02	-0.02	-0.03
White mullet (*Mugil curema*)	1	-0.13	0.42	-0.15	-0.09	-0.1	-0.04	-0.05	-0.06
White salema (*Xenichthys agassizii*)	1	0.13	0.07	0.1	0.03	-0.1	-0.08	-0.1	-0.09
Wounded wrasse (*Halichoeres chierchiae*)	1	0.17	0.15	0.1	0.04	-0.03	-0.12	-0.12	-0.1
Yellowtail damselfish (*Microspathodon bairdii*)	1	0.14	0.18	-0.02	0.02	-0.08	-0.08	-0.16	-0.09

Table S1: Averaged auto-correlation function (ACF) for observation lags from 0 to 8. Per transect 18 observations were made by three different observers subsequently. The considered response variables were the fourth-root transformed counts of every species. Auto-correlations were determined per transect and averaged afterwards. Per species, only transects were considered of which at least one observation had at least one observed individual. significant auto-correlations were indicated ($p<0.05$) with *.
