# Supplementary material for: Sampling errors and variability in video transects for assessment of reef fish assemblage structure and diversity
Source: PLoS One. 2022 Jul 25;17(7):e0271043. doi: 10.1371/journal.pone.0271043 (PMC9312474; doi:10.1371/journal.pone.0271043)
Supplement: S2 Table — (PDF) [file pone.0271043.s016.pdf]

Species	Variable	18 Observations~					6 Observations				
		Estimate	SE	z-value	p-value	n	Estimate	SE	z-value	p-value	n
Amarillo snapper	Intercept	-2.48	0.61	-4.05	0.000052	7	-1.13	0.56	-2.01	0.044399	14
Lutjanus argentiventris	Order	0.09	0.05	1.98	0.047997		0.06	0.14	0.42	0.671628	
Balloonfish	Intercept	-3.45	2.45	-1.41	0.159794	1	-4.93	4.61	-1.07	0.284486	1
Diodon holocanthus	Order	0.06	0.20	0.29	0.768144		0.79	0.94	0.85	0.397174	
Banded wrasse	Intercept	-0.87	0.31	-2.76	0.005776	21	0.12	0.38	0.32	0.747055	29
Halichoeres notospilus	Order	-0.03	0.02	-1.46	0.144351		-0.05	0.09	-0.57	0.565406	
Black striped salema	Intercept	-0.57	0.68	-0.84	0.401543	6	-0.12	0.84	-0.14	0.891117	10
Xenocys jessiae	Order	0.02	0.04	0.52	0.603868		0.14	0.15	0.92	0.356786	
Blacktip cardinalfish	Intercept	0.32	0.70	0.46	0.648619	12	0.49	0.66	0.74	0.457521	20
Apogon atradorsatus	Order	0.02	0.03	0.69	0.490417		0.09	0.11	0.82	0.409622	
Blue and gold snapper	Intercept	-2.23	0.72	-3.11	0.001862	7	0.47	0.71	0.67	0.504090	10
Lutjanus viridis	Order	0.04	0.05	0.79	0.429302		-0.41	0.18	-2.26	0.023927	
Blue chin parrotfish	Intercept	-1.67	0.63	-2.66	0.007844	6	-0.47	0.65	-0.72	0.470633	9
Scarus ghobban	Order	0.00	0.05	-0.08	0.939047		-0.04	0.17	-0.25	0.802441	
Bravo clinid	Intercept	0.20	0.38	0.53	0.598770	26	0.30	0.43	0.71	0.477527	50
Gobioclinus dendriticus	Order	0.00	0.02	-0.22	0.827335		0.12	0.07	1.76	0.078212	
Bullseye puffer	Intercept	0.28	0.65	0.44	0.661254	12	0.60	0.59	1.02	0.309205	24
Sphoeroides annulatus	Order	0.01	0.03	0.22	0.829083		0.01	0.10	0.05	0.95902	
Chameleon wrasse	Intercept	4.23	1.93	2.19	0.028528	9	4.27	2.03	2.11	0.035024	18
Halichoeres dispilus	Order	0.06	0.03	1.72	0.085938		0.18	0.11	1.62	0.104518	
Cortez rainbow wrasse	Intercept	4.58	1.04	4.4	0.000011	15	3.40	0.88	3.86	0.000113	29
Thalassoma lucasanum	Order	-0.11	0.03	-3.5	0.000460		-0.03	0.09	-0.37	0.713771	
Eagle ray	Intercept	-5.38	3.82	-1.41	0.158958	1	0.62	2.43	0.25	0.799435	1
Aetobatus narinari	Order	0.22	0.27	0.81	0.419141		-0.79	0.94	-0.85	0.397174	
Flag cabrilla	Intercept	-2.40	0.43	-5.63	0.001000	17	-1.38	0.47	-2.95	0.003156	23
Epinephelus labriformis	Order	0.02	0.04	0.59	0.552298		0.06	0.12	0.53	0.599324	
Galapagos bullhead shark	Intercept	-699.80	332061.42	0.00	0.998319	1	-253.12	470628.30	0.00	0.999571	1
Heterodontus quoyi	Order	40.00	19072.48	0.00	0.998327		46.06	86811.83	0.00	0.999577	
Galapagos grunt	Intercept	-1.47	0.55	-2.67	0.007593	6	-1.58	0.79	-1.99	0.046188	8
Orthopristis forbesi	Order	0.00	0.05	-0.02	0.980965		0.37	0.19	1.91	0.055509	
Galapagos ringtail damselfish	Intercept	6.84	2.26	3.02	0.002521	9	5.69	2.36	2.41	0.016135	17
Stegastes beebei	Order	0.03	0.04	0.89	0.375651		0.16	0.12	1.32	0.185678	
Galapagos Seabream	Intercept	0.34	0.75	0.45	0.653361	3	-0.53	0.88	-0.60	0.548921	7
Archosargus pourtalesi	Order	-0.01	0.06	-0.17	0.865352		0.33	0.20	1.63	0.103143	
Galapagos shark	Intercept	-7.21	3.19	-2.26	0.024065	3	-1.37	1.40	-0.98	0.327356	3
Carcharhinus galapagensis	Order	0.34	0.21	1.64	0.100432		-0.07	0.37	-0.18	0.853274	
Galapagos sheepshead wrasse	Intercept	-1.64	1.78	-0.92	0.357165	1	-253.12	470628.30	0.00	0.999571	1
Semicossyphus darwini	Order	-0.15	0.23	-0.66	0.511200		46.06	86811.83	0.00	0.999577	
Galapagos triplefin blenny	Intercept	-2.65	0	-930.18	0.001000	11	-0.36	0.57	-0.64	0.522337	14
Lepidonectes corallicola	Order	0.07	0	24.72	0.001000		-0.1	0.14	-0.69	0.488474	
Giant hawkfish	Intercept	-1.32	0.47	-2.79	0.005201	8	-1.34	0.62	-2.19	0.028840	13
Cirrhitus rivulatus	Order	-0.06	0.05	-1.29	0.196009		0.06	0.15	0.39	0.699636	
Harlequin wrasse	Intercept	-592.04	337281.7	0.00	0.998599	1	-117.08	268839.08	0.00	0.999653	1
Choerodon fasciatus	Order	40.83	23269.99	0.00	0.998600		46.79	103459.15	0.00	0.999639	
Jewel moray	Intercept	-3.52	0.95	-3.71	0.000209	6	-1.25	0.91	-1.37	0.170569	6
Muraena lentiginosa	Order	0.09	0.08	1.25	0.212205		0.00	0.23	0.00	1.000000	
Kingangelfish	Intercept	-2.8	0.66	-4.26	0.000002	8	-0.29	0.7	-0.42	0.676893	8
Holacanthus passer	Order	0.06	0.06	1.02	0.306740		-0.18	0.19	-0.92	0.355855	
Loosetooth parrotfish	Intercept	-1.29	1.75	-0.74	0.460641	1	-4.93	4.61	-1.07	0.284486	1
Nicholsina denticulata	Order	-0.22	0.27	-0.81	0.419141		0.79	0.94	0.85	0.397174	
Marbled goby	Intercept	-0.22	0.37	-0.59	0.555023	27	-0.05	0.43	-0.11	0.911877	39
Gobio manchada	Order	-0.05	0.02	-2.43	0.015042		0.11	0.08	1.44	0.151206	
Marbled ray	Intercept	-6.62	4.95	-1.34	0.181138	1	-0.92	2.33	-0.39	0.69451	1
Taeniurops meyeni	Order	0.30	0.33	0.92	0.356654		-0.21	0.66	-0.32	0.751098	
Mexican hogfish	Intercept	-2.00	0.39	-5.15	0.001000	20	-0.81	0.51	-1.57	0.117022	33
Bodianus diplotaenia	Order	0.07	0.03	2.68	0.007294		0.06	0.09	0.74	0.456656	
Mojarra grunt	Intercept	-1.93	0.79	-2.45	0.014254	4	-1.01	1.03	-0.98	0.329466	4
Haemulon scudderii	Order	-0.03	0.08	-0.42	0.674035		0.03	0.26	0.13	0.895424	
Mullet snapper	Intercept	-12.06	8.31	-1.45	0.146901	1	-2.92	2.68	-1.09	0.276547	1
Lutjanus aratus	Order	0.71	0.52	1.37	0.169709		0.59	0.62	0.95	0.344115	
Pacific dog snapper	Intercept	-12.15	6.75	-1.8	0.071946	1	0.92	2.33	0.39	0.694510	1
Lutjanus novemfasciatus	Order	0.90	0.50	1.81	0.070510		0.21	0.66	0.32	0.751098	
Pacific spotfin mojarra	Intercept	0.77	0.80	0.97	0.333770	9	1.32	0.64	2.05	0.040643	19
Eucinostomus dowii	Order	0.13	0.04	3.47	0.000528		0.17	0.11	1.58	0.114509	
Panamic fanged blenny	Intercept	-0.92	0.42	-2.19	0.028323	24	-0.72	0.38	-1.91	0.056012	46
Ophioblennius steindachneri	Order	0.01	0.02	0.63	0.526563		0.15	0.08	1.94	0.052235	
Panamic sergeant major	Intercept	2.20	2.51	0.88	0.380888	8	2.07	1.14	1.81	0.070653	10
Abudefduf troschelii	Order	0.31	0.07	4.61	0.000004		0.46	0.17	2.69	0.007223	
Razor surgeonfish	Intercept	-1.36	0.51	-2.65	0.007970	10	-0.26	0.47	-0.55	0.580781	21
Prionurus laticlavius	Order	0.06	0.03	1.63	0.102885		0.01	0.11	0.05	0.957375	
Reef cornetfish	Intercept	-0.92	0.50	-1.84	0.066457	6	-1.73	0.88	-1.95	0.050716	9
Fistularia commersonii	Order	-0.05	0.05	-1.11	0.266538		0.38	0.19	1.98	0.047591	
Sabertooth blenny	Intercept	0.79	0.38	2.07	0.038441	23	1.39	0.51	2.76	0.005756	45
Plagiotremus azaleus	Order	-0.01	0.02	-0.29	0.774761		-0.13	0.07	-1.77	0.077200	
Spinster wrasse	Intercept	1.34	0.57	2.35	0.018863	18	1.41	0.57	2.47	0.013542	32
Halichoeres nicholsi	Order	0.04	0.03	1.47	0.141888		0.18	0.09	1.93	0.052991	
Spotted cabrilla	Intercept	-699.8	332061.42	0.00	0.998319	1	-253.12	470628.30	0.00	0.999571	1
Epinephelus analogus	Order	40.00	19072.48	0.00	0.998327		46.06	86811.83	0.00	0.999577	
Stonescorpionfish	Intercept	0.32	0.81	0.39	0.697075	4	-0.22	1.14	-0.19	0.849113	4
Scorpaena mystes	Order	-0.36	0.13	-2.77	0.005677		-0.11	0.27	-0.41	0.684013	
Striped mullet	Intercept	-15.29	14.39	-1.06	0.287992	1	-4.93	4.61	-1.07	0.284486	1
Mugil cephalus	Order	0.84	0.86	0.98	0.325896		0.79	0.94	0.85	0.397174	
Three banded butterfly fish	Intercept	-0.73	0.74	-0.99	0.321277	8	0.04	0.76	0.06	0.953188	12
Chaetodon humeralis	Order	-0.01	0.04	-0.24	0.812474		0.01	0.15	0.08	0.940187	
Throatspotted blenny	Intercept	-1.59	0.88	-1.81	0.069802	3	0.19	1.16	0.17	0.867320	3
Malacoctenus tetranemus	Order	-0.08	0.09	-0.85	0.397326		-0.35	0.33	-1.05	0.293921	
Tiger snake eel	Intercept	-2.13	0.60	-3.55	0.000385	7	-0.57	0.66	-0.87	0.386257	10
Myrichthys maculosus	Order	0.02	0.05	0.43	0.665442		-0.10	0.17	-0.59	0.557025	
triplefin blenny	Intercept	0.71	2.30	0.31	0.756327	1	0.62	2.43	0.25	0.799435	1
Lepidonectes corallicola	Order	-0.84	0.86	-0.98	0.325896		-0.79	0.94	-0.85	0.397174	
White mullet	Intercept	-0.38	1.35	-0.28	0.779185	1	-5.24	4.16	-1.26	0.207754	1
Mugil curema	Order	-0.23	0.2	-1.17	0.243282		1.14	0.93	1.23	0.217323	
White salema	Intercept	-2.41	0.64	-3.75	0.00018	11	-0.38	0.72	-0.52	0.603209	13
Xenichthys agassizii	Order	0.08	0.04	2.12	0.033621		0.02	0.14	0.15	0.880997	
Wounded wrasse	Intercept	-1.65	0.46	-3.59	0.000336	10	0.00	0.73	0.01	0.994750	10
Halichoeres chierchiae	Order	0.01	0.04	0.13	0.893250		-0.06	0.15	-0.39	0.698345	
Yellowtail damselfish	Intercept	4.01	1.21	3.33	0.00088	13	4.96	0.00	5765.33	0.001000	30
Chrysiptera parasema	Order	0.01	0.03	0.43	0.664362		-0.19	0.00	-220.48	0.001000	

Table S2: Output of binomial mixed models with Presence/Absence as response variable, Order of the observation as fixed effect and Transect as random effect. The coefficients (Estimate), standard error (SE), z-value and p-value are given for each species. Significant positive effects ($p<0.05$) are indicated in green, Significant negative effects ($p<0.05$) are indicated in red. n represents the number of series of 6 or 18 repeats during which at least one individual of the concerning species was detected.
