# Supplementary material for: Sampling errors and variability in video transects for assessment of reef fish assemblage structure and diversity
Source: PLoS One. 2022 Jul 25;17(7):e0271043. doi: 10.1371/journal.pone.0271043 (PMC9312474; doi:10.1371/journal.pone.0271043)
Supplement: S3 Table — (PDF) [file pone.0271043.s017.pdf]

	Santa Cruz		Floreana	
	Spinster Wrasse	Sabertooth Blenny	Bravo Clinid	Panamic Fanged Blenny
CAP1	-0.815	-0.593	-0.645	0.665
CAP2	0.186	-0.414	0.426	0.420
CAP3	-0.071	-0.270	-0.025	-0.223
CAP4	0.172	0.093	-0.058	-0.224

Table S3: Selection of potential indicator species using CAP discriminant analysis with Location as grouping factor. CAP analysis was performed for each island. Four CAP axes were determined as optimal and correlations of each species with the axes were determined. The species with the highest correlations were retained and given in the table.
