# Supplementary material for: Sampling errors and variability in video transects for assessment of reef fish assemblage structure and diversity
Source: PLoS One. 2022 Jul 25;17(7):e0271043. doi: 10.1371/journal.pone.0271043 (PMC9312474; doi:10.1371/journal.pone.0271043)
Supplement: S4 Table — (PDF) [file pone.0271043.s018.pdf]

Species	ICC	10 meters	20 meters	30 meters	40 meters	50 meters
Spinster wrasse	$ICC_{Transect}$	0.107	0.021	0.041	0.05	0.054
	$ICC_{Location}$	0.651	0.188	0.294	0.383	0.494
	$ICC_{Observer}$	0.007	0.001	0.006	0.009	0.010
	$ICC_{Observer:Location}$	0.025	0.007	0.009	0.007	0.008
	$ICC_{Observer:Transect}$	0.000	0.000	0.000	0.001	0.003
	ICC_{Total}	0.790	0.217	0.350	0.450	0.569
	$ICC_{Sampling}$	0.235	0.790	0.659	0.558	0.442
Sabertooth blenny	$ICC_{Transect}$	0.108	0.106	0.131	0.165	0.188
	$ICC_{Location}$	0.683	0.677	0.765	0.583	0.593
	$ICC_{Observer}$	0.000	0.000	0.000	0.000	0.000
	$ICC_{Observer:Location}$	0.003	0.000	0.000	0.000	0.000
	$ICC_{Observer:Transect}$	0.092	0.041	0.046	0.043	0.026
	ICC_{Total}	0.886	0.824	0.942	0.791	0.807
	$ICC_{Sampling}$	0.209	0.217	0.104	0.252	0.219
Bravo clinid	$ICC_{Transect}$	0.362	0.178	0.120	0.098	0.099
	$ICC_{Location}$	0.184	0.162	0.153	0.159	0.163
	$ICC_{Observer}$	0.055	0.047	0.019	0.027	0.013
	$ICC_{Observer:Location}$	0.188	0.223	0.268	0.207	0.184
	$ICC_{Observer:Transect}$	0.041	0.062	0.040	0.043	0.043
	ICC_{Total}	0.830	0.672	0.600	0.534	0.502
	$ICC_{Sampling}$	0.399	0.613	0.708	0.716	0.725
Panamic fanged blenny	$ICC_{Transect}$	0.000	0.070	0.184	0.135	0.158
	$ICC_{Location}$	0.000	0.158	0.414	0.388	0.469
	$ICC_{Observer}$	0.000	0.002	0.010	0.012	0.010
	$ICC_{Observer:Location}$	0.000	0.000	0.000	0.000	0.005
	$ICC_{Observer:Transect}$	0.000	0.000	0.025	0.024	0.022
	ICC_{Total}	0.000	0.230	0.633	0.559	0.664
	$ICC_{Sampling}$	1.0000	0.770	0.392	0.465	0.363

Table S4: Model output (ICC (Intraclass Correlation Coefficient) of the random effects, total ICC explained by the factors of the model and ICC associated with the sampling variability) for generalized linear mixed models (zero-inflated Conway-Maxwell-Poisson) with either the count of the Spinster wrasse (*Halichoeres nicholsi*), Sabertooth blenny (*Plagiotremus azaleus*), Bravo clinid (*Gobioclinus dendriticus*) and Panamic fanged blenny (*Ophioblennius steindachneri*) as response, Location and Transect as nested random effects and Observer as crossed random effect. Transect lengths of 10, 20, 30, 40 and 50 meters were assessed.
