# Supplementary material for: Sampling errors and variability in video transects for assessment of reef fish assemblage structure and diversity
Source: PLoS One. 2022 Jul 25;17(7):e0271043. doi: 10.1371/journal.pone.0271043 (PMC9312474; doi:10.1371/journal.pone.0271043)
Supplement: S5 Table — (PDF) [file pone.0271043.s019.pdf]

.

Species	ICC *Transect*	ICC *Location*	ICC *Observer*	ICC *Observer: Location*	ICC *Observer: Transect*	ICC *Total*	ICC *Sampling*	Reaction to observer	Recorded response
Amarillo snapper	0.4584	0.5193	0.0000	0.0000	0.0000	0.9777	0.0223	2	attracted
Banded wrasse	0.0460	0.0303	0.0000	0.4178	0.0932	0.5873	0.9237	3	
Black-striped salema	0.0328	0.0005	0.0000	0.0014	0.0001	0.0348	0.9985	5	
Blacktip cardinalfish	0.0195	0.0000	0.0000	0.0005	0.0015	0.0215	0.9805	5	
Blue and gold snapper	0.8299	0.0000	0.0000	0.0232	0.0000	0.8531	0.1701	2	scared
Bluechin parrotfish	0.0052	0.0000	0.0000	0.0000	0.019	0.0242	0.9948	3	
Bravo clinid	0.0837	0.0328	0.0000	0.2844	0.0382	0.4391	0.8835	6	
Bullseye puffer	0.3082	0.3218	0.0196	0.0093	0.0238	0.6827	0.3504	5	
Chameleon wrasse	0.0121	0.1224	0.0000	0.0047	0.0085	0.1477	0.8655	3	
Cortez rainbow wrasse	0.0746	0.0621	0.0034	0.0026	0.0130	0.1557	0.8599	3	scared
Flag cabrilla	0.0999	0.2053	0.0000	0.0421	0.3869	0.7342	0.6948	5	
Galapagos grunt	0.0000	0.0000	0.0000	0.2610	0.0483	0.3093	1.0000	5	
Galapagos ringtail damselfish	0.1248	0.3435	0.0006	0.0156	0.0246	0.5091	0.5311	4	
Galapagos seabream	0.8974	0.0000	0.0000	0.0000	0.0187	0.9161	0.1026	4	
Galapagos triplefin blenny	0.0000	0.0000	0.0000	0.5883	0.3947	0.9830	1.0000	6	attracted
Giant hawkfish	0.7725	0.0000	0.0000	0.2037	0.0000	0.9762	0.2275	6	
Jewel moray	0.0001	0.0003	0.0000	0.0030	0.9937	0.9971	0.9996	6	
Marbled goby	0.0654	0.0765	0.0000	0.2367	0.0270	0.4056	0.8581	1	scared
Mexican hogfish	0.2232	0.0000	0.1765	0.0000	0.3115	0.7112	0.6003	5	attracted
Mojarra grunt	0.0000	0.0000	0.0000	0.0829	0.8992	0.9821	1.0000	4	
Mullet snapper	0.0000	0.0000	0.0000	0.0000	0.9914	0.9914	1.0000	2	
Pacific dog snapper	0.0000	0.0000	0.0000	0.0000	0.9899	0.9899	1.0000	2	
Pacific spotfin mojarra	0.0320	0.0109	0.0000	0.0037	0.0024	0.0490	0.9571	4	attracted
Panamic fanged blenny	0.1255	0.4897	0.0000	0.0300	0.0235	0.6687	0.3848	3	
Panamic sergeant major	0.0299	0.0739	0.0010	0.0000	0.0316	0.1364	0.8952	5	attracted
Razor surgeonfish	0.0191	0.0000	0.0000	0.0000	0.0028	0.0219	0.9809	4	
Reef cornetfish	0.0186	0.0362	0.0000	0.0000	0.9258	0.9806	0.9452	4	attracted
Sabertooth blenny	0.1248	0.2837	0.0017	0.0368	0.0412	0.4882	0.5898	3	
Spinster wrasse	0.1045	0.4102	0.0000	0.0207	0.0174	0.5528	0.4853	3	
Striped mullet	0.0543	0.0184	0.8041	0.0277	0.0753	0.9798	0.1232	4	
Three banded butterflyfish	0.6487	0.1585	0.0208	0.0000	0.0980	0.9260	0.1720	4	
Tiger snake eel	0.0000	0.1814	0.0000	0.0000	0.4897	0.6711	0.8186	1	
White mullet	0.0000	0.0000	0.0000	0.0000	0.9961	0.9961	1.0000	4	
White salema	0.0535	0.0000	0.0000	0.2214	0.0020	0.2769	0.9465	5	attracted
Wounded wrasse	0.0679	0.0000	0.0000	0.4247	0.0000	0.4926	0.9321	4	
Yellowtail damselfish	0.0055	0.0045	0.0002	0.0019	0.0001	0.0122	0.9898	5	scared

Table S5: ICC values of generalized linear mixed models (zero-inflated Conway-Maxwell-Poisson) with the counts of the different observed species as response, Island as fixed effect, Location and Transect as nested random effects and Observer as crossed random effect. The data originating from the full 50-meter transect was used. The reaction-to-observer provides a literature-based score from 1, for seemingly shy and easily frightened species, to 6, for seemingly curious species (Humann and Deloach, 2003). The recorded response provides an estimate of the recorded behavior of different species. Only the significant results of the binomial mixed models, given in Table D.2., are given.
