# Supplementary material for: Sampling errors and variability in video transects for assessment of reef fish assemblage structure and diversity
Source: PLoS One. 2022 Jul 25;17(7):e0271043. doi: 10.1371/journal.pone.0271043 (PMC9312474; doi:10.1371/journal.pone.0271043)
Supplement: S6 Table — (PDF) [file pone.0271043.s020.pdf]

Observations pooled	Dissimilarity	Transect length (meters) 10	20	30	40	50
1	NA	0.007	0.000	0.000	0.000	0.000
2	NA	0.000	0.000	0.000	0.000	0.000
3	NA	0.000	0.000	0.000	0.000	0.000
4	NA	0.000	0.000	0.000	0.000	0.000
5	NA	0.000	0.000	0.000	0.000	0.000
6	NA	0.000	0.000	0.000	0.000	0.000
1	0	0.0908	0.014	0.014	0.007	0.000
2	0	0.0094	0.000	0.000	0.000	0.000
3	0	0.0026	0.000	0.000	0.000	0.000
4	0	0.0013	0.000	0.000	0.000	0.000
5	0	0.000	0.000	0.000	0.000	0.000
6	0	0.000	0.000	0.000	0.000	0.000
1	1	4.2488	0.9783	0.3494	0.0699	0.000
2	1	0.1689	0.0177	0.0023	0.000	0.000
3	1	0.0065	0,001	0.000	0.000	0.000
4	1	0.000	0.000	0.000	0.000	0.000
5	1	0.000	0.000	0.000	0.000	0.000
6	1	0.000	0.000	0.000	0.000	0.000

Table S6: Effect of different pooling scenarios (1 to 6 observations with steps of 1) within locations for different transect lengths on the percentage of undefined (NA) dissimilarity values and perfectly similar (0) and dissimilar (1) observations. Monte Carlo simulations (n=10000) were applied to pool the observations.
