# Supplementary material for: Sampling errors and variability in video transects for assessment of reef fish assemblage structure and diversity
Source: PLoS One. 2022 Jul 25;17(7):e0271043. doi: 10.1371/journal.pone.0271043 (PMC9312474; doi:10.1371/journal.pone.0271043)
Supplement: S8 Table — (PDF) [file pone.0271043.s022.pdf]

Source	df	MS	pseudo-F	p-value	Unique perms
Island	1	445960	15.17	0.001	999
Observer	2	3913	1.52	0.248	60
Location	8	26981	2.90	0.001	994
Island X Observer	2	2578	1.06	0.400	999
Transect	20	7222	7.32	0.001	997
Location X Observer	16	2437	2.47	0.001	997
Transect X Observer	40	987	3.63	0.001	998
Residuals	450	272			

Table S8: PERMANOVA testing for effect of Island as fixed factor, Location and Transect as nested random factors and Observer as crossed random factor on the structure of the observed fish assemblages. PERMANOVA done on Bray-Curtis dissimilarity matrices (fourth-root transformation).

	10 meters	20 meters	30 meters	40 meters	50 meters
R^2_{Island}	0.420**	0.418**	0.428**	0.429**	0.437**
$R^2_{Observer}$	0.006	0.005	0.006	0.007	0.008
$R^2_{Location}$	0.145**	0.165**	0.178**	0.196**	0.212**
$R^2_{Island:Observer}$	0.003	0.004	0.004	0.004	0.004
$R^2_{Transect}$	0.145**	0.132**	0.140**	0.144**	0.142**
$R^2_{Location:Observer}$	0.022*	0.028*	0.027*	0.027*	0.031*
$R^2_{Transect:Observer}$	0.045**	0.046**	0.042**	0.040**	0.039**
$R^2_{VideoAnalyst}$	0.003	0.007	0.007	0.008	0.008
$R^2_{Residuals}$	0.239	0.193	0.166	0.144	0.120

Table S9: Goodness-of-fit (R^2) estimates for PERMANOVA models based on Bray-Curtis dissimilarities (fourth-root transformation) with Island as fixed factor, Location and Transect as nested random factors and Observer and Video analyst as crossed random factors for transect lengths of 10, 20, 30, 40 and 50 meters.
