# Supplementary material for: Sampling errors and variability in video transects for assessment of reef fish assemblage structure and diversity
Source: PLoS One. 2022 Jul 25;17(7):e0271043. doi: 10.1371/journal.pone.0271043 (PMC9312474; doi:10.1371/journal.pone.0271043)
Supplement: S1 Appendix — (PDF) [file pone.0271043.s024.pdf]

Appendix A. Additional information

Appendix A.1. Instantaneous fish displacement

As fish displacement is not necessarily random, even at very short time scales, we prefer to use the term instantaneous fish displacement, instead of random fish displacement (Irigoyen et al., 2013; MacNeil et al., 2008), throughout the article to include all fish displacement that would take place under natural conditions at very short time scales. While the instantaneous displacement causes actual variation in the local (i.e. within sampling unit) abundance, the random counting/detection errors introduce false variation in local abundance.

Appendix A.2. Applications of video monitoring

Video material has been used for different purposes at different times after the video material was collected. It has been used to characterize physical habitats (Cappo et al., 2003), to obtain biomass estimates (Harvey et al., 2001), to study fish behavior (Watson and Harvey, 2007), and to train algorithms to pick out fish and identify them automatically (Boom et al., 2012). In addition, because the collecting and analyzing of the video material can be done by two different persons, people trained in underwater video collection do not necessarily have to be trained in identifying and counting fish.

Appendix B. Map

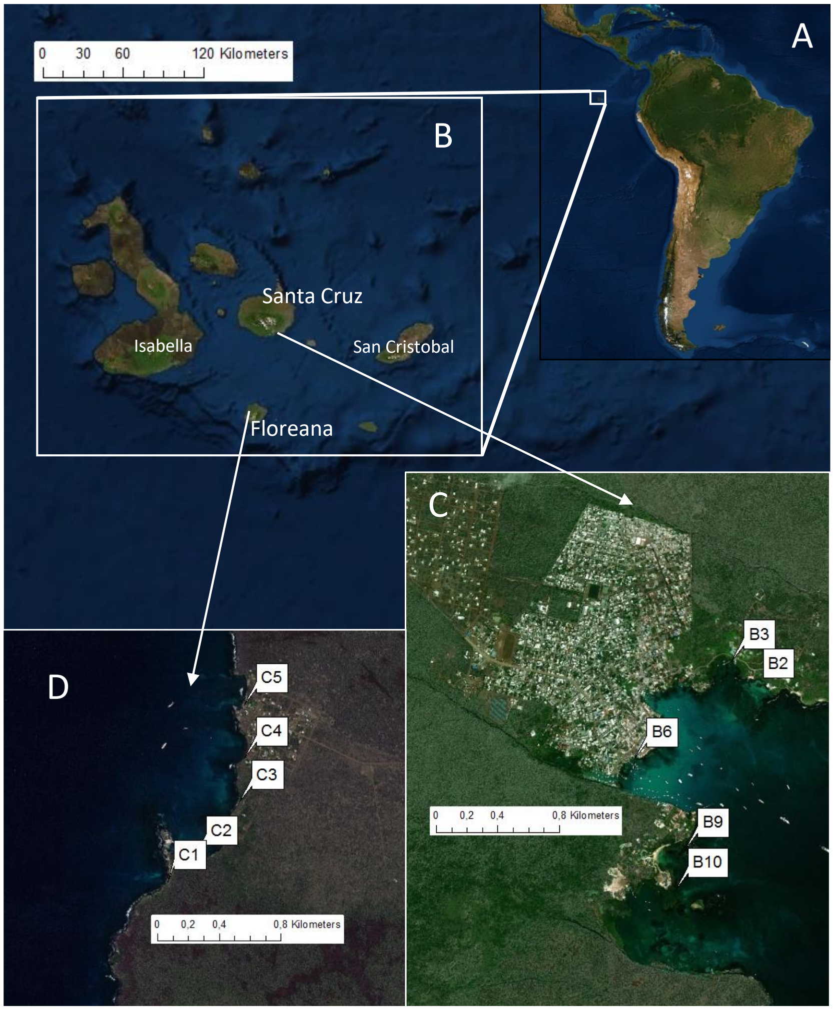

Figure B.1: Map of the study area. (**A**) South-American continent with depiction of the Galapagos archipelago. (**B**) Galapagos archipelago with depiction of the two studied islands. (**C**) The city Puerto Ayora on Santa Cruz island with indication of the study locations. (**D**) The city Puerto Velazco Ibarra on Floreana island with indication of the study locations. Landsat 8 imagery was used to construct the maps.

Appendix C. Video analysis

Fish were counted in such a way that both the MinCount and MaxCount of each observation could be determined. For example, if two individuals of the same species were recorded in frame$_1$ and a few moments later a third individual of the same species entered the field of view (FOV) of frame$_2$, while the other two individuals were still present in the FOV, than only the recording of frame$_2$ would be included. However, if the two individuals encountered in frame$_1$ moved away before the individual of frame$_2$ moved within the FOV, then both recordings were included separately as the third individual may actually have been one of the first two individuals. An easier way to determine MinCount would have been to only note down the maximum number of individuals. For example, if first two individuals would be recorded then the video analyst would write down two. If then three individuals would be recorded the video analyst would change that number to three. If less than three individuals would then be recorded this would not be included. For the MaxCount, the first method was actually the most efficient one and since MaxCount is typically used for video transects (Mallet and Pelletier, 2014; Wartenberg and Booth, 2015), this method was chosen.

Appendix D. Independence of observations

Species	lag 0	lag 1	lag 2	lag 3	lag 4	lag 5	lag 6	lag 7	lag 8
Amarillo snapper (*Lutjanus argentiventris*)	1	-0.03	-0.05	-0.02	-0.11	0	-0.03	-0.1	-0.01
Balloon fish (*Diodon holocanthus*)	1	-0.06	-0.07	-0.07	-0.07	-0.08	-0.08	-0.08	-0.03
Banded wrasse (*Halichoeres notospilus*)	1	0.2	0.16	0.03	-0.03	-0.07	-0.13	-0.15	-0.07
Black striped salema (*Xenocys jessiae*)	1	-0.01	0.03	0.02	0.03	-0.01	-0.04	-0.09	-0.05
Blacktip cardinalfish (*Apogon atradorsatus*)	1	0.06	0.14	-0.03	0.04	-0.08	-0.09	-0.13	-0.11
Blue and gold snapper (*Lutjanus viridis*)	1	0.06	0.07	-0.09	-0.11	-0.09	-0.1	-0.03	-0.04
Blue chin parrotfish (*Scarus ghobban*)	1	0.02	0.01	0.04	0.02	-0.07	-0.07	-0.03	-0.04
Bravo clinid (*Gobioclinus dendriticus*)	1	0.16	0.11	0.01	0.02	-0.03	-0.08	-0.1	-0.14
Bullseye puffer (*Sphoeroides annulatus*)	1	0.09	-0.05	-0.07	0.01	-0.04	-0.1	-0.11	-0.06
Chameleon wrasse (*Halichoeres dispilus*)	1	0.29	0.18	0	0	-0.03	-0.09	-0.09	-0.09
Cortez rainbow wrasse (*Thalassoma lucasanum*)	1	0.13	0.17	0	-0.01	-0.07	-0.13	-0.1	-0.06
Eagle ray (*Aetobatus narinari*)	1	-0.06	-0.07	-0.07	-0.07	-0.02	-0.02	-0.02	-0.03
Flag cabrilla (*Epinephelus labriformis*)	1	0.04	0.01	-0.04	-0.07	-0.06	-0.07	-0.03	-0.04
Galapagos bullhead shark (*Heterodontus quoyi*)	1	0	-0.01	-0.01	-0.01	-0.02	-0.02	-0.02	-0.03
Galapagos grunt (*Orthopristis forbesi*)	1	0.21	0.03	0.01	0.09	-0.01	-0.17	-0.11	-0.07
Galapagos ringtail damselfish (*Stegastes beebei*)	1	0.26	0.22	0.04	0.03	-0.11	-0.12	-0.11	-0.15
Galapagos Seabream (*Archosargus pourtalesi*)	1	0.27	-0.05	0	0.09	-0.06	-0.18	-0.04	-0.08
Galapagos shark (*Carcharhinus galapagensis*)	1	-0.06	-0.05	-0.05	-0.03	-0.02	-0.02	-0.02	-0.03
Galapagos sheepshead wrasse (*Semicossyphus darwini*)	1	-0.06	-0.07	-0.07	-0.07	-0.08	-0.02	-0.02	-0.03
Galapagos triplefin blenny (*Lepidonectes corallicola*)	1	0.16	-0.03	-0.01	-0.04	-0.04	-0.08	-0.07	-0.03
Giant hawkfish (*Cirrhitus rivulatus*)	1	-0.02	0.13	-0.03	-0.07	-0.07	-0.14	-0.05	-0.1
Harlequin wrasse (*Bodianus eclancheri*)	1	0.73*	0.47*	0.2	-0.06	-0.08	-0.1	-0.11	-0.13
Jewel moray (*Muraena lentiginosa*)	1	-0.06	-0.06	-0.06	0.13	-0.05	-0.06	-0.05	-0.05
King angelfish (*Holacanthus passer*)	1	0.06	-0.02	-0.02	-0.01	-0.05	-0.07	-0.06	-0.06
Loosetooth parrotfish (*Nicholsina denticulata*)	1	-0.06	-0.07	-0.07	-0.07	-0.02	-0.02	-0.02	-0.03
Marbled goby (*Gobio manchada*)	1	0.17	0.15	0.04	0.02	-0.06	-0.11	-0.12	-0.12
Marbled ray (*Taeniurops meyeni*)	1	-0.06	-0.07	-0.07	-0.01	-0.02	-0.02	-0.02	-0.03
Mexican hogfish (*Bodianus diplotaenia*)	1	0.11	0.07	0.04	0.05	-0.07	-0.04	-0.07	-0.08
Mojarra grunt (*Haemulon scudderii*)	1	0	0.03	0	0.01	-0.05	-0.04	-0.04	-0.05
Mullet snapper (*Lutjanus aratus*)	1	0.42	-0.08	-0.02	-0.03	-0.03	-0.04	-0.05	-0.06
Pacific dog snapper (*Lutjanus novemfasciatus*)	1	0.46*	0.2	0.29	0.32	0.14	-0.13	-0.15	-0.17
Pacific spotfin mojarra (*Eucinostomus dowii*)	1	0.14	0.09	0.07	0.03	0.01	0.02	-0.11	-0.04
Panamic fanged blenny (*Ophioblennius steindachneri*)	1	0	0	-0.07	0	-0.09	-0.07	-0.04	-0.03
Panamic sergeant major (*Abudefduf troschelii*)	1	0.37	0.19	0.07	0.04	-0.06	-0.1	-0.14	-0.08
Razor surgeonfish (*Prionurus laticlavius*)	1	0.06	0.16	-0.09	-0.1	-0.07	-0.15	-0.06	-0.05
Reef cornetfish (*Fistularia commersonii*)	1	0.29	0.06	-0.04	-0.03	-0.02	-0.09	-0.08	-0.08
Sabertooth blenny (*Plagiotremus azaleus*)	1	0.12	0.08	0.01	-0.04	-0.11	-0.12	-0.13	-0.07
Spinster wrasse (*Halichoeres nicholsi*)	1	0.11	0.12	0.02	-0.03	-0.06	-0.06	-0.04	-0.05
Spotted cabrilla (*Epinephelus analogus*)	1	0	-0.01	-0.01	-0.01	-0.02	-0.02	-0.02	-0.03
Stone scorpionfish (*Scorpaena mystes*)	1	0.27	0.08	0.04	-0.02	-0.08	-0.07	-0.08	-0.08
Striped mullet (*Mugil cephalus*)	1	-0.06	-0.01	-0.01	-0.01	-0.02	-0.02	-0.02	-0.03
Three banded butterfly fish (*Chaetodon humeralis*)	1	0.07	0.05	0.04	-0.04	-0.13	0.04	-0.04	-0.07
Throat-spotted blenny (*Malacoctenus tetranemus*)	1	0.29	-0.07	-0.06	-0.06	-0.07	-0.08	-0.08	-0.09
Tiger snake eel (*Myrichthys maculosus*)	1	0.04	-0.07	-0.08	-0.01	-0.04	-0.05	-0.13	-0.09
Triplefin blenny (*Lepidonectes corallicola*)	1	-0.06	-0.01	-0.01	-0.01	-0.02	-0.02	-0.02	-0.03
White mullet (*Mugil curema*)	1	-0.13	0.42	-0.15	-0.09	-0.1	-0.04	-0.05	-0.06
White salema (*Xenichthys agassizii*)	1	0.13	0.07	0.1	0.03	-0.1	-0.08	-0.1	-0.09
Wounded wrasse (*Halichoeres chierchiae*)	1	0.17	0.15	0.1	0.04	-0.03	-0.12	-0.12	-0.1
Yellowtail damselfish (*Microspathodon bairdii*)	1	0.14	0.18	-0.02	0.02	-0.08	-0.08	-0.16	-0.09

Table D.1: Averaged auto-correlation function (ACF) for observation lags from 0 to 8. Per transect 18 observations were made by three different observers subsequently. The considered response variables were the fourth-root transformed counts of every species. Auto-correlations were determined per transect and averaged afterwards. Per species, only transects were considered of which at least one observation had at least one observed individual. significant auto-correlations were indicated ($p<0.05$) with *.

Species	Variable	18 Observations~					6 Observations				
		Estimate	SE	z-value	p-value	n	Estimate	SE	z-value	p-value	n
Amarillo snapper	Intercept	-2.48	0.61	-4.05	0.000052	7	-1.13	0.56	-2.01	0.044399	14
Lutjanus argentiventris	Order	0.09	0.05	1.98	0.047997		0.06	0.14	0.42	0.671628	
Balloonfish	Intercept	-3.45	2.45	-1.41	0.159794	1	-4.93	4.61	-1.07	0.284486	1
Diodon holocanthus	Order	0.06	0.20	0.29	0.768144		0.79	0.94	0.85	0.397174	
Banded wrasse	Intercept	-0.87	0.31	-2.76	0.005776	21	0.12	0.38	0.32	0.747055	29
Halichoeres notospilus	Order	-0.03	0.02	-1.46	0.144351		-0.05	0.09	-0.57	0.565406	
Black striped salema	Intercept	-0.57	0.68	-0.84	0.401543	6	-0.12	0.84	-0.14	0.891117	10
Xenocys jessiae	Order	0.02	0.04	0.52	0.603868		0.14	0.15	0.92	0.356786	
Blacktip cardinalfish	Intercept	0.32	0.70	0.46	0.648619	12	0.49	0.66	0.74	0.457521	20
Apogon atradorsatus	Order	0.02	0.03	0.69	0.490417		0.09	0.11	0.82	0.409622	
Blue and gold snapper	Intercept	-2.23	0.72	-3.11	0.001862	7	0.47	0.71	0.67	0.504090	10
Lutjanus viridis	Order	0.04	0.05	0.79	0.429302		-0.41	0.18	-2.26	0.023927	
Blue chin parrotfish	Intercept	-1.67	0.63	-2.66	0.007844	6	-0.47	0.65	-0.72	0.470633	9
Scarus ghobban	Order	0.00	0.05	-0.08	0.939047		-0.04	0.17	-0.25	0.802441	
Bravo clinid	Intercept	0.20	0.38	0.53	0.598770	26	0.30	0.43	0.71	0.477527	50
Gobioclinus dendriticus	Order	0.00	0.02	-0.22	0.827335		0.12	0.07	1.76	0.078212	
Bullseye puffer	Intercept	0.28	0.65	0.44	0.661254	12	0.60	0.59	1.02	0.309205	24
Sphoeroides annulatus	Order	0.01	0.03	0.22	0.829083		0.01	0.10	0.05	0.95902	
Chameleon wrasse	Intercept	4.23	1.93	2.19	0.028528	9	4.27	2.03	2.11	0.035024	18
Halichoeres dispilus	Order	0.06	0.03	1.72	0.085938		0.18	0.11	1.62	0.104518	
Cortez rainbow wrasse	Intercept	4.58	1.04	4.4	0.000011	15	3.40	0.88	3.86	0.000113	29
Thalassoma lucasanum	Order	-0.11	0.03	-3.5	0.000460		-0.03	0.09	-0.37	0.713771	
Eagle ray	Intercept	-5.38	3.82	-1.41	0.158958	1	0.62	2.43	0.25	0.799435	1
Aetobatus narinari	Order	0.22	0.27	0.81	0.419141		-0.79	0.94	-0.85	0.397174	
Flag cabrilla	Intercept	-2.40	0.43	-5.63	0.001000	17	-1.38	0.47	-2.95	0.003156	23
Epinephelus labriformis	Order	0.02	0.04	0.59	0.552298		0.06	0.12	0.53	0.599324	
Galapagos bullhead shark	Intercept	-699.80	332061.42	0.00	0.998319	1	-253.12	470628.30	0.00	0.999571	1
Heterodontus quoyi	Order	40.00	19072.48	0.00	0.998327		46.06	86811.83	0.00	0.999577	
Galapagos grunt	Intercept	-1.47	0.55	-2.67	0.007593	6	-1.58	0.79	-1.99	0.046188	8
Orthopristis forbesi	Order	0.00	0.05	-0.02	0.980965		0.37	0.19	1.91	0.055509	
Galapagos ringtail damselfish	Intercept	6.84	2.26	3.02	0.002521	9	5.69	2.36	2.41	0.016135	17
Stegastes beebei	Order	0.03	0.04	0.89	0.375651		0.16	0.12	1.32	0.185678	
Galapagos Seabream	Intercept	0.34	0.75	0.45	0.653361	3	-0.53	0.88	-0.60	0.548921	7
Archosargus pourtalesi	Order	-0.01	0.06	-0.17	0.865352		0.33	0.20	1.63	0.103143	
Galapagos shark	Intercept	-7.21	3.19	-2.26	0.024065	3	-1.37	1.40	-0.98	0.327356	3
Carcharhinus galapagensis	Order	0.34	0.21	1.64	0.100432		-0.07	0.37	-0.18	0.853274	
Galapagos sheepshead wrasse	Intercept	-1.64	1.78	-0.92	0.357165	1	-253.12	470628.30	0.00	0.999571	1
Semicossyphus darwini	Order	-0.15	0.23	-0.66	0.511200		46.06	86811.83	0.00	0.999577	
Galapagos triplefin blenny	Intercept	-2.65	0	-930.18	0.001000	11	-0.36	0.57	-0.64	0.522337	14
Lepidonectes corallicola	Order	0.07	0	24.72	0.001000		-0.1	0.14	-0.69	0.488474	
Giant hawkfish	Intercept	-1.32	0.47	-2.79	0.005201	8	-1.34	0.62	-2.19	0.028840	13
Cirrhitus rivulatus	Order	-0.06	0.05	-1.29	0.196009		0.06	0.15	0.39	0.699636	
Harlequin wrasse	Intercept	-592.04	337281.7	0.00	0.998599	1	-117.08	268839.08	0.00	0.999653	1
Choerodon fasciatus	Order	40.83	23269.99	0.00	0.998600		46.79	103459.15	0.00	0.999639	
Jewel moray	Intercept	-3.52	0.95	-3.71	0.000209	6	-1.25	0.91	-1.37	0.170569	6
Muraena lentiginosa	Order	0.09	0.08	1.25	0.212205		0.00	0.23	0.00	1.000000	
Kingangelfish	Intercept	-2.8	0.66	-4.26	0.000002	8	-0.29	0.7	-0.42	0.676893	8
Holacanthus passer	Order	0.06	0.06	1.02	0.306740		-0.18	0.19	-0.92	0.355855	
Loosetooth parrotfish	Intercept	-1.29	1.75	-0.74	0.460641	1	-4.93	4.61	-1.07	0.284486	1
Nicholsina denticulata	Order	-0.22	0.27	-0.81	0.419141		0.79	0.94	0.85	0.397174	
Marbled goby	Intercept	-0.22	0.37	-0.59	0.555023	27	-0.05	0.43	-0.11	0.911877	39
Gobio manchada	Order	-0.05	0.02	-2.43	0.015042		0.11	0.08	1.44	0.151206	
Marbled ray	Intercept	-6.62	4.95	-1.34	0.181138	1	-0.92	2.33	-0.39	0.69451	1
Taeniurops meyeni	Order	0.30	0.33	0.92	0.356654		-0.21	0.66	-0.32	0.751098	
Mexican hogfish	Intercept	-2.00	0.39	-5.15	0.001000	20	-0.81	0.51	-1.57	0.117022	33
Bodianus diplotaenia	Order	0.07	0.03	2.68	0.007294		0.06	0.09	0.74	0.456656	
Mojarra grunt	Intercept	-1.93	0.79	-2.45	0.014254	4	-1.01	1.03	-0.98	0.329466	4
Haemulon scudderii	Order	-0.03	0.08	-0.42	0.674035		0.03	0.26	0.13	0.895424	
Mullet snapper	Intercept	-12.06	8.31	-1.45	0.146901	1	-2.92	2.68	-1.09	0.276547	1
Lutjanus aratus	Order	0.71	0.52	1.37	0.169709		0.59	0.62	0.95	0.344115	
Pacific dog snapper	Intercept	-12.15	6.75	-1.8	0.071946	1	0.92	2.33	0.39	0.694510	1
Lutjanus novemfasciatus	Order	0.90	0.50	1.81	0.070510		0.21	0.66	0.32	0.751098	
Pacific spotfin mojarra	Intercept	0.77	0.80	0.97	0.333770	9	1.32	0.64	2.05	0.040643	19
Eucinostomus dowii	Order	0.13	0.04	3.47	0.000528		0.17	0.11	1.58	0.114509	
Panamic fanged blenny	Intercept	-0.92	0.42	-2.19	0.028323	24	-0.72	0.38	-1.91	0.056012	46
Ophioblennius steindachneri	Order	0.01	0.02	0.63	0.526563		0.15	0.08	1.94	0.052235	
Panamic sergeant major	Intercept	2.20	2.51	0.88	0.380888	8	2.07	1.14	1.81	0.070653	10
Abudefduf troschelii	Order	0.31	0.07	4.61	0.000004		0.46	0.17	2.69	0.007223	
Razor surgeonfish	Intercept	-1.36	0.51	-2.65	0.007970	10	-0.26	0.47	-0.55	0.580781	21
Prionurus laticlavius	Order	0.06	0.03	1.63	0.102885		0.01	0.11	0.05	0.957375	
Reef cornetfish	Intercept	-0.92	0.50	-1.84	0.066457	6	-1.73	0.88	-1.95	0.050716	9
Fistularia commersonii	Order	-0.05	0.05	-1.11	0.266538		0.38	0.19	1.98	0.047591	
Sabertooth blenny	Intercept	0.79	0.38	2.07	0.038441	23	1.39	0.51	2.76	0.005756	45
Plagiotremus azaleus	Order	-0.01	0.02	-0.29	0.774761		-0.13	0.07	-1.77	0.077200	
Spinster wrasse	Intercept	1.34	0.57	2.35	0.018863	18	1.41	0.57	2.47	0.013542	32
Halichoeres nicholsi	Order	0.04	0.03	1.47	0.141888		0.18	0.09	1.93	0.052991	
Spotted cabrilla	Intercept	-699.8	332061.42	0.00	0.998319	1	-253.12	470628.30	0.00	0.999571	1
Epinephelus analogus	Order	40.00	19072.48	0.00	0.998327		46.06	86811.83	0.00	0.999577	
Stonescorpionfish	Intercept	0.32	0.81	0.39	0.697075	4	-0.22	1.14	-0.19	0.849113	4
Scorpaena mystes	Order	-0.36	0.13	-2.77	0.005677		-0.11	0.27	-0.41	0.684013	
Striped mullet	Intercept	-15.29	14.39	-1.06	0.287992	1	-4.93	4.61	-1.07	0.284486	1
Mugil cephalus	Order	0.84	0.86	0.98	0.325896		0.79	0.94	0.85	0.397174	
Three banded butterfly fish	Intercept	-0.73	0.74	-0.99	0.321277	8	0.04	0.76	0.06	0.953188	12
Chaetodon humeralis	Order	-0.01	0.04	-0.24	0.812474		0.01	0.15	0.08	0.940187	
Throatspotted blenny	Intercept	-1.59	0.88	-1.81	0.069802	3	0.19	1.16	0.17	0.867320	3
Malacoctenus tetranemus	Order	-0.08	0.09	-0.85	0.397326		-0.35	0.33	-1.05	0.293921	
Tiger snake eel	Intercept	-2.13	0.60	-3.55	0.000385	7	-0.57	0.66	-0.87	0.386257	10
Myrichthys maculosus	Order	0.02	0.05	0.43	0.665442		-0.10	0.17	-0.59	0.557025	
triplefin blenny	Intercept	0.71	2.30	0.31	0.756327	1	0.62	2.43	0.25	0.799435	1
Lepidonectes corallicola	Order	-0.84	0.86	-0.98	0.325896		-0.79	0.94	-0.85	0.397174	
White mullet	Intercept	-0.38	1.35	-0.28	0.779185	1	-5.24	4.16	-1.26	0.207754	1
Mugil curema	Order	-0.23	0.2	-1.17	0.243282		1.14	0.93	1.23	0.217323	
White salema	Intercept	-2.41	0.64	-3.75	0.00018	11	-0.38	0.72	-0.52	0.603209	13
Xenichthys agassizii	Order	0.08	0.04	2.12	0.033621		0.02	0.14	0.15	0.880997	
Wounded wrasse	Intercept	-1.65	0.46	-3.59	0.000336	10	0.00	0.73	0.01	0.994750	10
Halichoeres chierchiae	Order	0.01	0.04	0.13	0.893250		-0.06	0.15	-0.39	0.698345	
Yellowtail damselfish	Intercept	4.01	1.21	3.33	0.00088	13	4.96	0.00	5765.33	0.001000	30
Chrysiptera parasema	Order	0.01	0.03	0.43	0.664362		-0.19	0.00	-220.48	0.001000	

Table D.2: Output of binomial mixed models with Presence/Absence as response variable, Order of the observation as fixed effect and Transect as random effect. The coefficients (Estimate), standard error (SE), z-value and p-value are given for each species. Significant positive effects ($p<0.05$) are indicated in green, Significant negative effects ($p<0.05$) are indicated in red. n represents the number of series of 6 or 18 repeats during which at least one individual of the concerning species was detected.

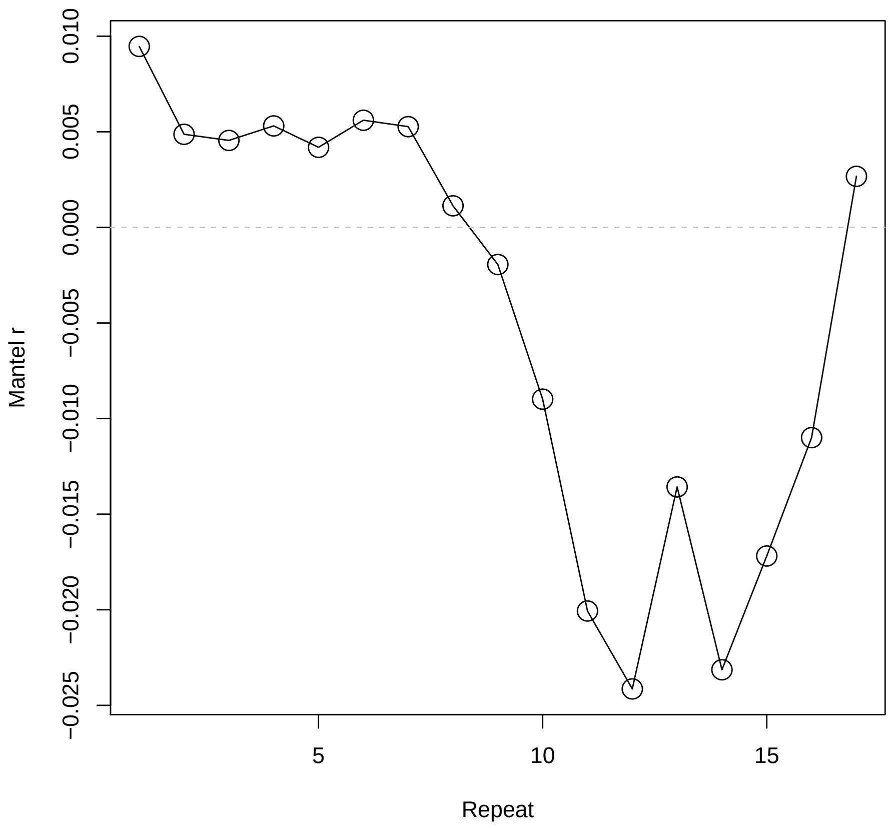

Figure D.1: Partial mantel correlogram for temporal auto-correlation of the observed structure of fish assemblages. The effect of the sampling units themselves was partialled out. No significant temporal auto-correlations were found.

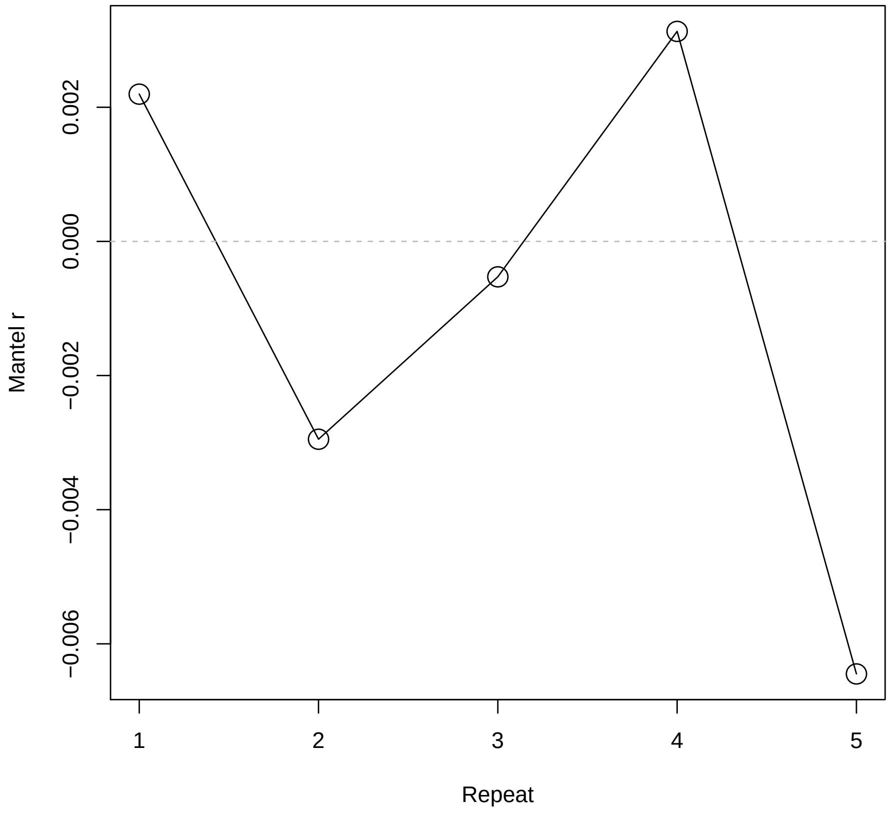

Figure D.2: Partial mantel correlogram for temporal auto-correlation of the observed structure of fish assemblages. The effect of the sampling units and observers themselves was partialled out. No significant temporal auto-correlations were found.

Appendix E. Indicator species

Applied ecological studies often assess the potential of different species to serve as indicators for water quality. For each island, two potential indicator species were determined using CAP analysis based on the group-

ing factor Location. The species with the highest Spearman correlation to the CAP axes (m=4) were retained (Table E.1), as they are representative for observed differences among groups. The full 50 meter dataset was split up for the two islands and separate CAP analyses were performed. Data was fourth-root transformed and Bray-Curtis dissimilarities were calculated. We decided to perform one CAP analysis per island as the differences in species between the islands may be more related to biogeography and dispersal limitations than to environmental conditions which dictate the water quality. The Spinster Wrasse (*Halichoeres nicholsi*) and Sabertooth Blenny (*Plagiotremus azaleus*) were selected for Santa Cruz, while the Bravo Clinid (*Gobioclinus dendriticus*) and Panamic Fanged Blenny (*Ophioblennius steindachneri*) were selected for Floreana. It should be noted that these species might not be actual indicator species for water quality, as the constrained ordination was performed on an artificial grouping factor, rather than on actual environmental gradients.

	Santa Cruz		Floreana	
	Spinster Wrasse	Sabertooth Blenny	Bravo Clinid	Panamic Fanged Blenny
CAP1	-0.815	-0.593	-0.645	0.665
CAP2	0.186	-0.414	0.426	0.420
CAP3	-0.071	-0.270	-0.025	-0.223
CAP4	0.172	0.093	-0.058	-0.224

Table E.1: Selection of potential indicator species using CAP discriminant analysis with Location as grouping factor. CAP analysis was performed for each island. Four CAP axes were determined as optimal and correlations of each species with the axes were determined. The species with the highest correlations were retained and given in the table.

Appendix F. Species Accumulation Curves (SACs)

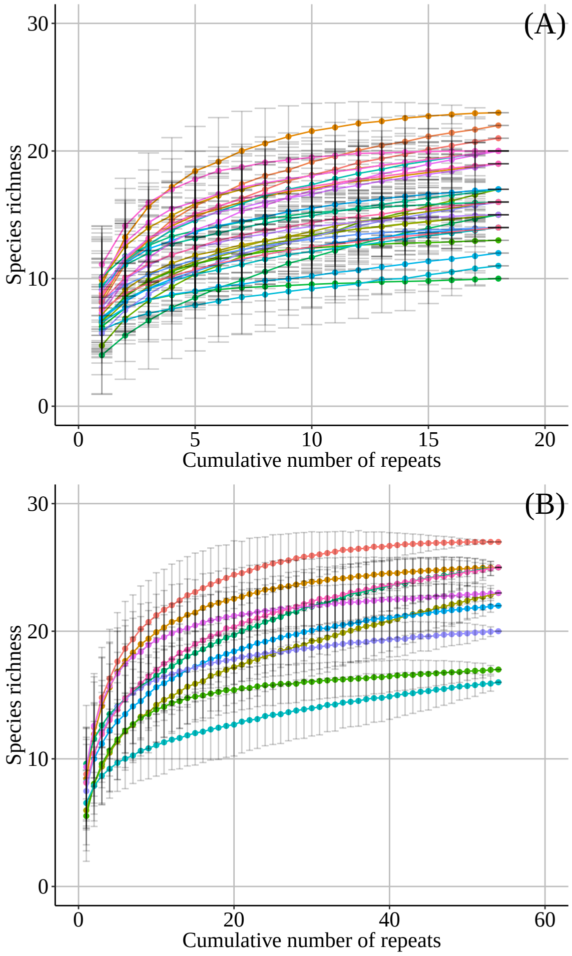

Figure F.1: Species Accumulation Curves (SACs) depicting the cumulative species richness in function of the cumulative number of repeats. Permutations or randomizations without replacement (n=10^4) of the repeats were used to determine the mean and standard deviation. The error bars represent the 95% confidence interval. In (A) the repeats were pooled within Transect, resulting in one SAC per transect (n=18). In (B) the repeats were pooled within Location, resulting in one SAC per location (n=54).

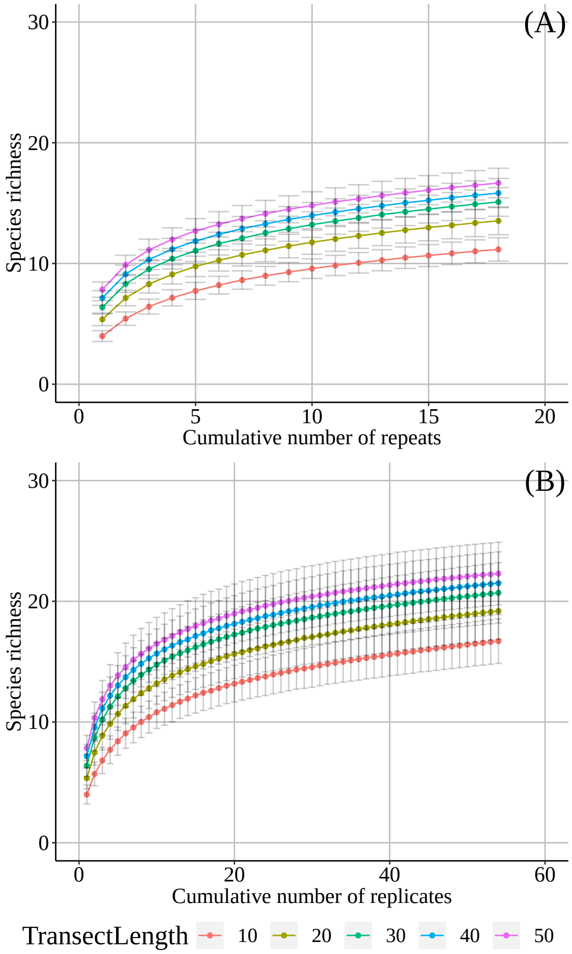

Figure F.2: Species Accumulation Curves (SACs) depicting the cumulative species richness in function of the cumulative number of repeats for different transect lengths. Permutations or randomizations without replacement (n=10^4) of the repeats were used to determine the mean and standard deviation. The error bars represent the 95% confidence interval. In (A) the repeats were pooled within Transect (n=18). In (B) the repeats were pooled within Location (n=54).

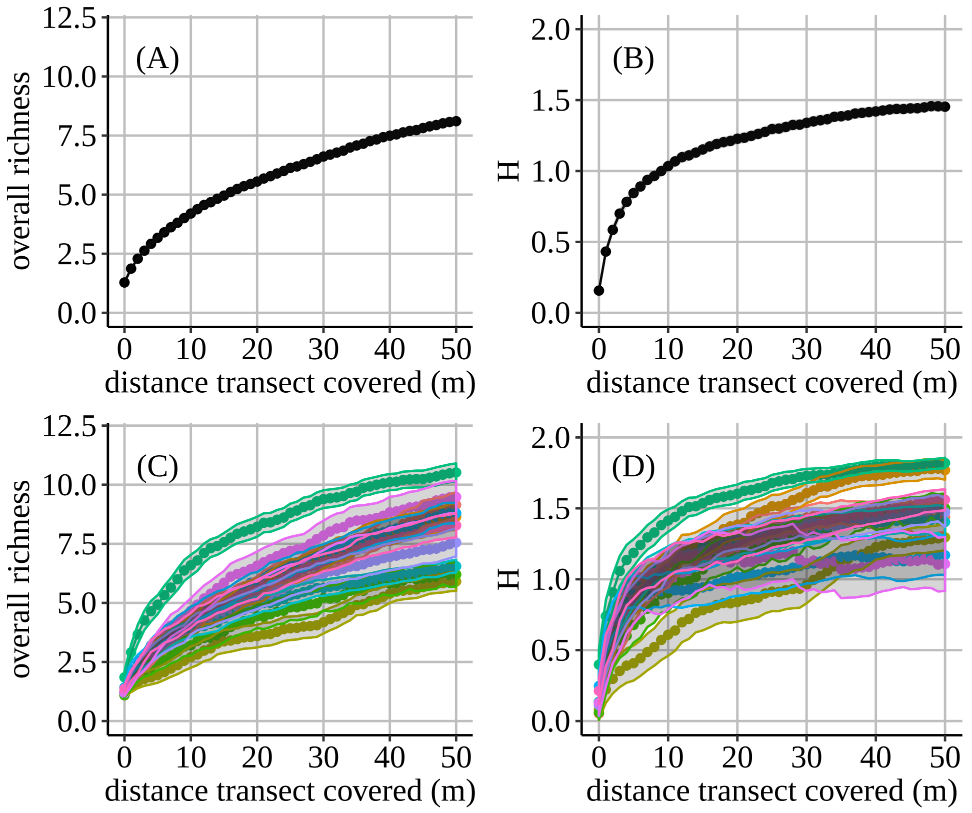

Figure F.3: Species Accumulation Curves (SACs) depicting the cumulative species density and cumulative Shannon diversity (H) in function of the transect distance. The error bars represent the 95% confidence interval. In (A) and (B) the average cumulative species density and Shannon diversity of all observations (n=540) are depicted respectively. In (C) and (D) a distinction is made between the 10 locations (n=54).

Appendix G. Generalized linear mixed models

Species	ICC	10 meters	20 meters	30 meters	40 meters	50 meters
Spinster wrasse	$ICC_{Transect}$	0.107	0.021	0.041	0.05	0.054
	$ICC_{Location}$	0.651	0.188	0.294	0.383	0.494
	$ICC_{Observer}$	0.007	0.001	0.006	0.009	0.010
	$ICC_{Observer:Location}$	0.025	0.007	0.009	0.007	0.008
	$ICC_{Observer:Transect}$	0.000	0.000	0.000	0.001	0.003
	ICC_{Total}	0.790	0.217	0.350	0.450	0.569
	$ICC_{Sampling}$	0.235	0.790	0.659	0.558	0.442
Sabertooth blenny	$ICC_{Transect}$	0.108	0.106	0.131	0.165	0.188
	$ICC_{Location}$	0.683	0.677	0.765	0.583	0.593
	$ICC_{Observer}$	0.000	0.000	0.000	0.000	0.000
	$ICC_{Observer:Location}$	0.003	0.000	0.000	0.000	0.000
	$ICC_{Observer:Transect}$	0.092	0.041	0.046	0.043	0.026
	ICC_{Total}	0.886	0.824	0.942	0.791	0.807
	$ICC_{Sampling}$	0.209	0.217	0.104	0.252	0.219
Bravo clinid	$ICC_{Transect}$	0.362	0.178	0.120	0.098	0.099
	$ICC_{Location}$	0.184	0.162	0.153	0.159	0.163
	$ICC_{Observer}$	0.055	0.047	0.019	0.027	0.013
	$ICC_{Observer:Location}$	0.188	0.223	0.268	0.207	0.184
	$ICC_{Observer:Transect}$	0.041	0.062	0.040	0.043	0.043
	ICC_{Total}	0.830	0.672	0.600	0.534	0.502
	$ICC_{Sampling}$	0.399	0.613	0.708	0.716	0.725
Panamic fanged blenny	$ICC_{Transect}$	0.000	0.070	0.184	0.135	0.158
	$ICC_{Location}$	0.000	0.158	0.414	0.388	0.469
	$ICC_{Observer}$	0.000	0.002	0.010	0.012	0.010
	$ICC_{Observer:Location}$	0.000	0.000	0.000	0.000	0.005
	$ICC_{Observer:Transect}$	0.000	0.000	0.025	0.024	0.022
	ICC_{Total}	0.000	0.230	0.633	0.559	0.664
	$ICC_{Sampling}$	1.0000	0.770	0.392	0.465	0.363

Table G.1: Model output (ICC (Intraclass Correlation Coefficient) of the random effects, total ICC explained by the factors of the model and ICC associated with the sampling variability) for generalized linear mixed models (zero-inflated Conway-Maxwell-Poisson) with either the count of the Spinster wrasse (*Halichoeres nicholsi*), Sabertooth blenny (*Plagiotremus azaleus*), Bravo clinid (*Gobioclinus dendriticus*) and Panamic fanged blenny (*Ophioblennius steindachneri*) as response, Location and Transect as nested random effects and Observer as crossed random effect. Transect lengths of 10, 20, 30, 40 and 50 meters were assessed.

.

Species	ICC *Transect*	ICC *Location*	ICC *Observer*	ICC *Observer: Location*	ICC *Observer: Transect*	ICC *Total*	ICC *Sampling*	Reaction to observer	Recorded response
Amarillo snapper	0.4584	0.5193	0.0000	0.0000	0.0000	0.9777	0.0223	2	attracted
Banded wrasse	0.0460	0.0303	0.0000	0.4178	0.0932	0.5873	0.9237	3	
Black-striped salema	0.0328	0.0005	0.0000	0.0014	0.0001	0.0348	0.9985	5	
Blacktip cardinalfish	0.0195	0.0000	0.0000	0.0005	0.0015	0.0215	0.9805	5	
Blue and gold snapper	0.8299	0.0000	0.0000	0.0232	0.0000	0.8531	0.1701	2	scared
Bluechin parrotfish	0.0052	0.0000	0.0000	0.0000	0.019	0.0242	0.9948	3	
Bravo clinid	0.0837	0.0328	0.0000	0.2844	0.0382	0.4391	0.8835	6	
Bullseye puffer	0.3082	0.3218	0.0196	0.0093	0.0238	0.6827	0.3504	5	
Chameleon wrasse	0.0121	0.1224	0.0000	0.0047	0.0085	0.1477	0.8655	3	
Cortez rainbow wrasse	0.0746	0.0621	0.0034	0.0026	0.0130	0.1557	0.8599	3	scared
Flag cabrilla	0.0999	0.2053	0.0000	0.0421	0.3869	0.7342	0.6948	5	
Galapagos grunt	0.0000	0.0000	0.0000	0.2610	0.0483	0.3093	1.0000	5	
Galapagos ringtail damselfish	0.1248	0.3435	0.0006	0.0156	0.0246	0.5091	0.5311	4	
Galapagos seabream	0.8974	0.0000	0.0000	0.0000	0.0187	0.9161	0.1026	4	
Galapagos triplefin blenny	0.0000	0.0000	0.0000	0.5883	0.3947	0.9830	1.0000	6	attracted
Giant hawkfish	0.7725	0.0000	0.0000	0.2037	0.0000	0.9762	0.2275	6	
Jewel moray	0.0001	0.0003	0.0000	0.0030	0.9937	0.9971	0.9996	6	
Marbled goby	0.0654	0.0765	0.0000	0.2367	0.0270	0.4056	0.8581	1	scared
Mexican hogfish	0.2232	0.0000	0.1765	0.0000	0.3115	0.7112	0.6003	5	attracted
Mojarra grunt	0.0000	0.0000	0.0000	0.0829	0.8992	0.9821	1.0000	4	
Mullet snapper	0.0000	0.0000	0.0000	0.0000	0.9914	0.9914	1.0000	2	
Pacific dog snapper	0.0000	0.0000	0.0000	0.0000	0.9899	0.9899	1.0000	2	
Pacific spotfin mojarra	0.0320	0.0109	0.0000	0.0037	0.0024	0.0490	0.9571	4	attracted
Panamic fanged blenny	0.1255	0.4897	0.0000	0.0300	0.0235	0.6687	0.3848	3	
Panamic sergeant major	0.0299	0.0739	0.0010	0.0000	0.0316	0.1364	0.8952	5	attracted
Razor surgeonfish	0.0191	0.0000	0.0000	0.0000	0.0028	0.0219	0.9809	4	
Reef cornetfish	0.0186	0.0362	0.0000	0.0000	0.9258	0.9806	0.9452	4	attracted
Sabertooth blenny	0.1248	0.2837	0.0017	0.0368	0.0412	0.4882	0.5898	3	
Spinster wrasse	0.1045	0.4102	0.0000	0.0207	0.0174	0.5528	0.4853	3	
Striped mullet	0.0543	0.0184	0.8041	0.0277	0.0753	0.9798	0.1232	4	
Three banded butterflyfish	0.6487	0.1585	0.0208	0.0000	0.0980	0.9260	0.1720	4	
Tiger snake eel	0.0000	0.1814	0.0000	0.0000	0.4897	0.6711	0.8186	1	
White mullet	0.0000	0.0000	0.0000	0.0000	0.9961	0.9961	1.0000	4	
White salema	0.0535	0.0000	0.0000	0.2214	0.0020	0.2769	0.9465	5	attracted
Wounded wrasse	0.0679	0.0000	0.0000	0.4247	0.0000	0.4926	0.9321	4	
Yellowtail damselfish	0.0055	0.0045	0.0002	0.0019	0.0001	0.0122	0.9898	5	scared

Table G.2: ICC values of generalized linear mixed models (zero-inflated Conway-Maxwell-Poisson) with the counts of the different observed species as response, Island as fixed effect, Location and Transect as nested random effects and Observer as crossed random effect. The data originating from the full 50-meter transect was used. The reaction-to-observer provides a literature-based score from 1, for seemingly shy and easily frightened species, to 6, for seemingly curious species (Humann and Deloach, 2003). The recorded response provides an estimate of the recorded behavior of different species. Only the significant results of the binomial mixed models, given in Table D.2., are given.

Appendix H. Design error

Observations pooled	Dissimilarity	Transect length (meters)				
		10	20	30	40	50
1	NA	0.007	0.000	0.000	0.000	0.000
2	NA	0.000	0.000	0.000	0.000	0.000
3	NA	0.000	0.000	0.000	0.000	0.000
4	NA	0.000	0.000	0.000	0.000	0.000
5	NA	0.000	0.000	0.000	0.000	0.000
6	NA	0.000	0.000	0.000	0.000	0.000
1	0	0.0908	0.014	0.014	0.007	0.000
2	0	0.0094	0.000	0.000	0.000	0.000
3	0	0.0026	0.000	0.000	0.000	0.000
4	0	0.0013	0.000	0.000	0.000	0.000
5	0	0.000	0.000	0.000	0.000	0.000
6	0	0.000	0.000	0.000	0.000	0.000
1	1	4.2488	0.9783	0.3494	0.0699	0.000
2	1	0.1689	0.0177	0.0023	0.000	0.000
3	1	0.0065	0,001	0.000	0.000	0.000
4	1	0.000	0.000	0.000	0.000	0.000
5	1	0.000	0.000	0.000	0.000	0.000
6	1	0.000	0.000	0.000	0.000	0.000

Table H.1: Effect of different pooling scenarios (1 to 6 observations with steps of 1) within locations for different transect lengths on the percentage of undefined (NA) dissimilarity values and perfectly similar (0) and dissimilar (1) observations. Monte Carlo simulations (n=10000) were applied to pool the observations.

Observations pooled	Dissimilarity	Transect length (meters) 10	20	30	40	50
1	NA	0.0023	0.000	0.000	0.000	0.000
2	NA	0.000	0.000	0.000	0.000	0.000
3	NA	0.000	0.000	0.000	0.000	0.000
4	NA	0.000	0.000	0.000	0.000	0.000
5	NA	0.000	0.000	0.000	0.000	0.000
6	NA	0.000	0.000	0.000	0.000	0.000
1	0	0.0116	0.0023	0.0047	0.0023	0.000
2	0	0.0019	0.000	0.000	0.000	0.000
3	0	0,001	0.000	0.000	0.000	0.000
4	0	0.000	0.000	0.000	0.000	0.000
5	0	0.000	0.000	0.000	0.000	0.000
6	0	0.000	0.000	0.000	0.000	0.000
1	1	0.2399	0.0349	0.014	0.000	0.000
2	1	0.0141	0,001	0.000	0.000	0.000
3	1	0.0017	0.000	0.000	0.000	0.000
4	1	0.000	0.000	0.000	0.000	0.000
5	1	0.000	0.000	0.000	0.000	0.000
6	1	0.000	0.000	0.000	0.000	0.000

Table H.2: Effect of different pooling scenarios (1 to 6 observations with steps of 1) within transects for different transect lengths on the percentage of undefined (NA) dissimilarity values and perfectly similar (0) and dissimilar (1) observations. Monte Carlo simulations (n=10000) were applied to pool the observations.

1087 Appendix I. PERMANOVA

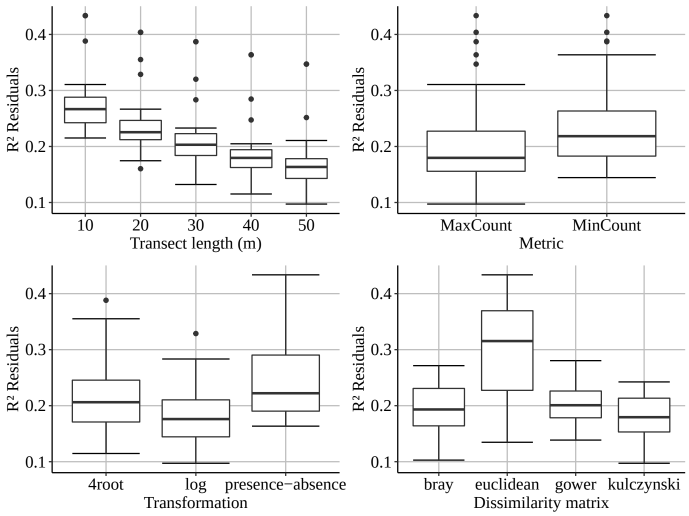

Figure I.1: R^2 of the residuals of the PERMANOVA models with Island as fixed factor, Location and Transect as nested random factors and Observer as crossed random factor. $R^2_{Residuals}$ is defined here as the ratio of the sum of squares of the error over the total sum of squares. Different parameters were assessed including the transect length (10, 20, 30, 40 and 50 meters), metric (MaxCount and MinCount), transformation (4root, logarithm and presence-absence) and the method to calculate the dissimilarity matrix (Bray-Curtis, Euclidean, Gower and Kulczynski).

Source	df	MS	pseudo-F	p-value	Unique perms
Island	1	445960	15.17	0.001	999
Observer	2	3913	1.52	0.248	60
Location	8	26981	2.90	0.001	994
Island X Observer	2	2578	1.06	0.400	999
Transect	20	7222	7.32	0.001	997
Location X Observer	16	2437	2.47	0.001	997
Transect X Observer	40	987	3.63	0.001	998
Residuals	450	272			

Table I.1: PERMANOVA testing for effect of Island as fixed factor, Location and Transect as nested random factors and Observer as crossed random factor on the structure of the observed fish assemblages. PERMANOVA done on Bray-Curtis dissimilarity matrices (fourth-root transformation).

	10 meters	20 meters	30 meters	40 meters	50 meters
R^2_{Island}	0.420**	0.418**	0.428**	0.429**	0.437**
$R^2_{Observer}$	0.006	0.005	0.006	0.007	0.008
$R^2_{Location}$	0.145**	0.165**	0.178**	0.196**	0.212**
$R^2_{Island:Observer}$	0.003	0.004	0.004	0.004	0.004
$R^2_{Transect}$	0.145**	0.132**	0.140**	0.144**	0.142**
$R^2_{Location:Observer}$	0.022*	0.028*	0.027*	0.027*	0.031*
$R^2_{Transect:Observer}$	0.045**	0.046**	0.042**	0.040**	0.039**
$R^2_{VideoAnalyst}$	0.003	0.007	0.007	0.008	0.008
$R^2_{Residuals}$	0.239	0.193	0.166	0.144	0.120

Table I.2: Goodness-of-fit (R^2) estimates for PERMANOVA models based on Bray-Curtis dissimilarities (fourth-root transformation) with Island as fixed factor, Location and Transect as nested random factors and Observer and Video analyst as crossed random factors for transect lengths of 10, 20, 30, 40 and 50 meters.

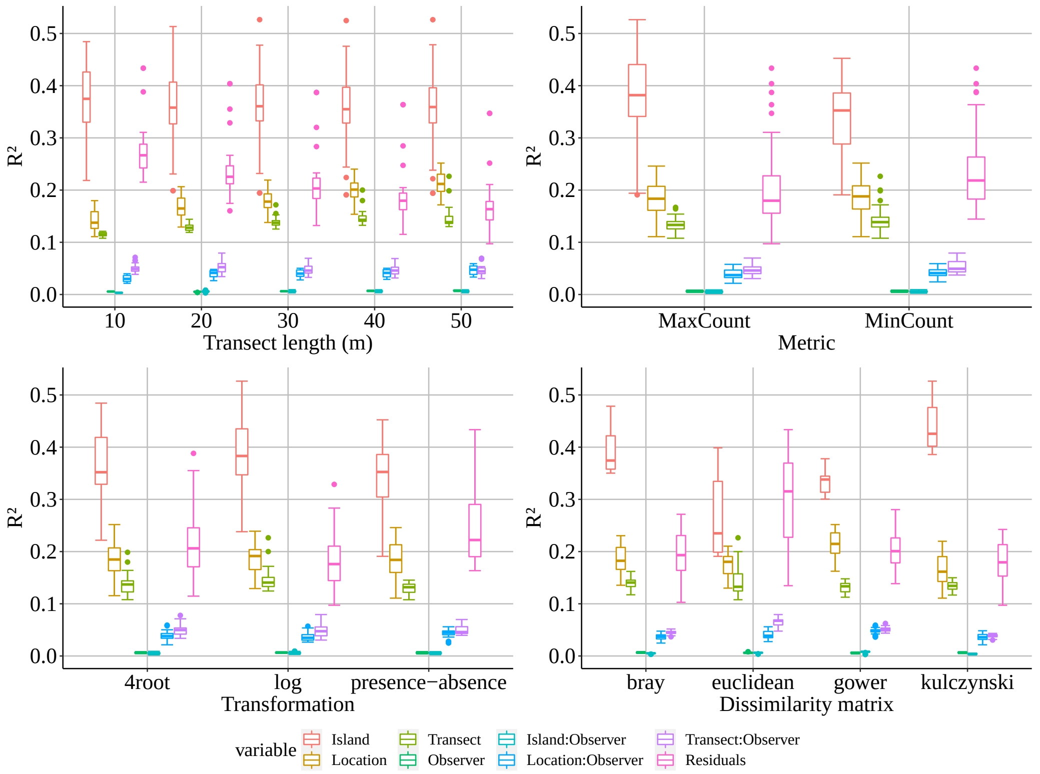

Figure I.2: R^2 of the different factors of the PERMANOVA models with Island as fixed factor, Location and Transect as nested random factors and Observer and its interactions with the other factors as crossed random factors. R^2 is defined here as the ratio of the sum of squares over the total sum of squares. Different parameters were assessed including the transect length (10, 20, 30, 40 and 50 meters), metric (MaxCount and MinCount), transformation (4root, logarithm and presence absence data) and the method to calculate the dissimilarity matrix (Bray-Curtis, Euclidean, Gower and Kulczynski).

1088 Appendix J. Precision

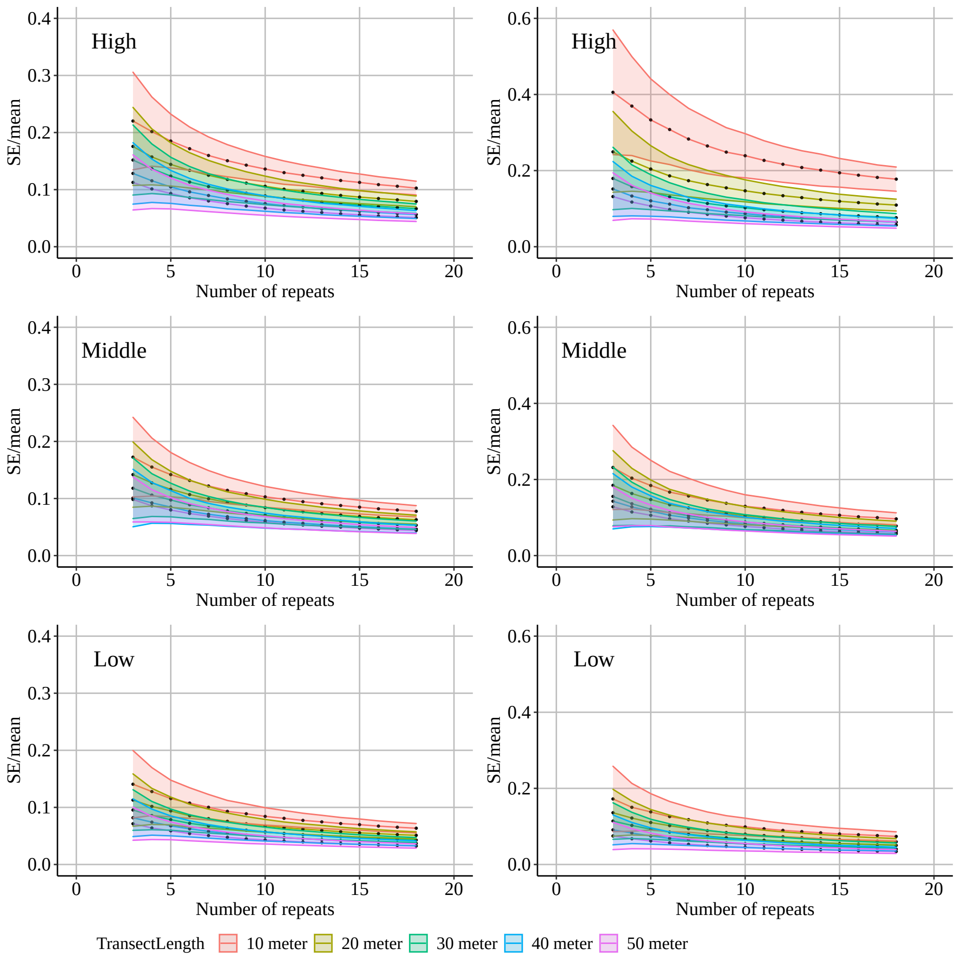

Figure J.1: Precision estimates of species density (left) and Shannon diversity (right) with precision defined as the inverse of the standard error over the mean. The 30 transects were divided in 3 equally sized groups with different levels of turbidity (High, Middle and Low). Different transect lengths were considered ranging from 10, 20, 30, 40 to 50 meters. Monte Carlo simulations (n=10^4) were applied to determine the precision per transect. The average precision over all transects is visualized. The error bars represent the 95% confidence intervals which were constructed using the pooled standard deviation of the estimates.

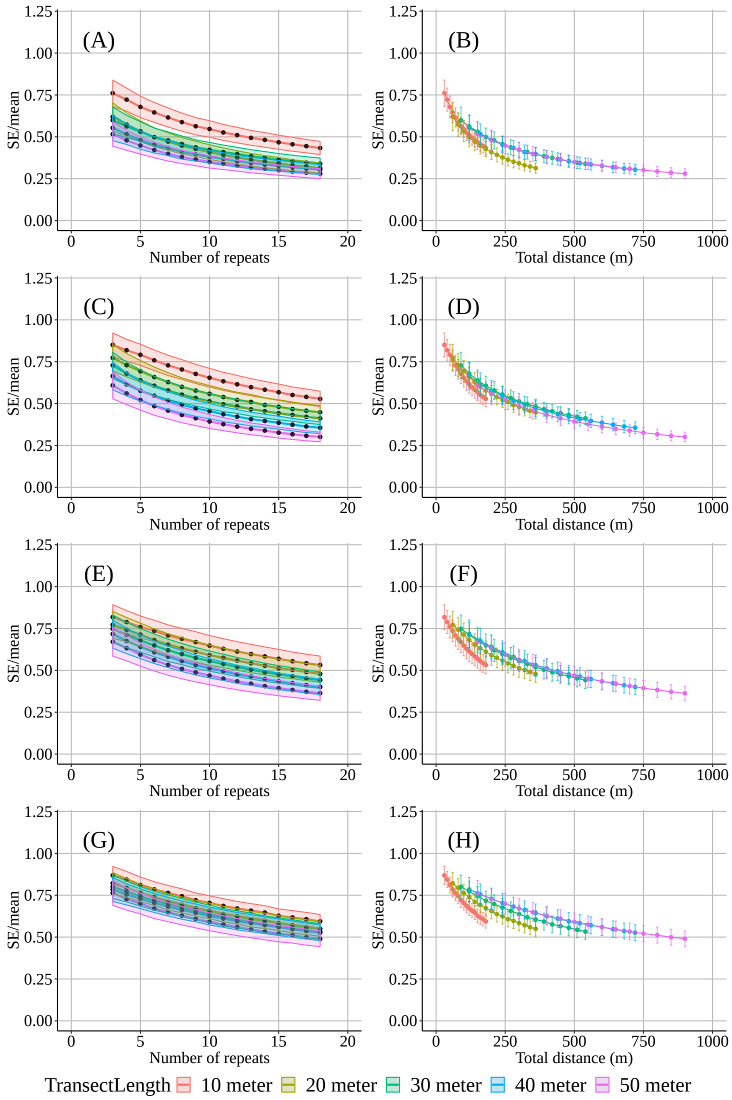

Figure J.2: Precision estimates of abundances (N) of different potential indicator species in function of the number of repeats (A,C,E,G) and in function of the total swim distance (B,D,F,H). Precision is defined as the inverse of the standard error over the mean. Total swim distance is defined as the number of repeats multiplied with the transect length. The potential indicator species were Spinster Wrasse (*Halichoeres nicholsi*); A, B, Sabertooth Blenny (*Plagiotremus azaleus*); C, D, Bravo Clinid (*Gobioclinus dendriticus*); E, F, and Panamic Fanged Blenny (*Ophioblennius steindachneri*)); G, H. Different transect lengths were considered ranging from 10, 20, 30, 40 to 50 meters. Monte Carlo simulations ($n=10^4$) were applied to determine the precision per transect. The average precision over all transects is visualized. The error bars represent the 95% confidence intervals which were constructed using the pooled standard deviation of the estimates.
