# Supplementary material for: Sampling errors and variability in video transects for assessment of reef fish assemblage structure and diversity
Source: PLoS One. 2022 Jul 25;17(7):e0271043. doi: 10.1371/journal.pone.0271043 (PMC9312474; doi:10.1371/journal.pone.0271043)
Supplement: Attachment — Submitted filename: PONE-D-21-06060_errors and variability in video transects.pdf [file pone.0271043.s025.pdf]

PLOS ONE

Sampling errors and variability in video transects for assessment of reef fish assemblage structure and diversity

--Manuscript Draft--

**Manuscript Number:**	PONE-D-21-06060
**Article Type:**	Research Article
**Full Title:**	Sampling errors and variability in video transects for assessment of reef fish assemblage structure and diversity
**Short Title:**	Video transects for reef fish assemblages
**Corresponding Author:**	Stijn Bruneel<br>Ghent University: Universiteit Gent<br>Ghent, BELGIUM
**Keywords:**	Video transects; Fish; Experimental design; Inter-observer variability; Design error; Precision
**Abstract:**	Video monitoring is a rapidly evolving tool in aquatic ecological research because of its non-destructive ability to assess fish assemblages. Nevertheless, methodological considerations of video monitoring techniques are often overlooked, especially in more complex sampling designs, causing inefficient data collection, processing, and interpretation. In this study, we discuss how video transect sampling designs should be assessed and how the inter-observer variability, design errors and sampling variability should be quantified and accounted for. The study took place in the coastal areas of the Galapagos archipelago and consisted of a hierarchical repeated-observations sampling design with multiple observers. Although observer bias was negligible for the assessment of fish assemblage structure, diversity and counts of individual species, sampling variability caused by simple counting/detection errors, observer effects and instantaneous fish displacement was often important. Especially for the counts of individual species, sampling variability most often exceeded the variability of the transects and sites. An extensive part of the variability in the fish assemblage structure was explained by the different transects (13%), suggesting that a sufficiently high number of transects is required to account for the within-location variability. Longer transect lengths allowed a better representation of the fish assemblages as sampling variability decreased with 33% if transect length was increased from 10 to 50 meters. However, to increase precision, including more repeats was typically more efficient than using longer transect lengths. The results confirm the suitability of the technique to study reef fish assemblages, but also highlight the importance of a sound methodological assessment since different biological responses and sampling designs are associated with different levels of sampling variability, precision and ecological relevance. Therefore, besides the direct usefulness of the developed guidelines, the procedures to establish them may be just as valuable for researchers aiming to optimize their own sampling technique and design.
**Order of Authors:**	Stijn Bruneel
	Long Ho
	Wout Van Echelpoel
	Amber Schoeters
	Heleen Raat
	Tom Moens
	Rafael Bermudez
	Stijn Luca
	Peter Goethals
**Additional Information:**	
**Question**	**Response**

Powered by Editorial Manager® and ProduXion Manager® from Aries Systems Corporation

**Financial Disclosure**<br><br>Enter a financial disclosure statement that describes the sources of funding for the work included in this submission. Review the submission guidelines for detailed requirements. View published research articles from *PLOS ONE* for specific examples.<br><br>This statement is required for submission and **will appear in the published article** if the submission is accepted. Please make sure it is accurate.<br><br>**Unfunded studies**<br>Enter: *The author(s) received no specific funding for this work.*<br><br>**Funded studies**<br>Enter a statement with the following details:<br>• Initials of the authors who received each award<br>• Grant numbers awarded to each author<br>• The full name of each funder<br>• URL of each funder website<br>• Did the sponsors or funders play any role in the study design, data collection and analysis, decision to publish, or preparation of the manuscript?<br>• **NO** - Include this sentence at the end of your statement: *The funders had no role in study design, data collection and analysis, decision to publish, or preparation of the manuscript.*<br>• **YES** - Specify the role(s) played.<br><br>* typeset	The research was partly funded by VLIR-UOS Biodiversity Network Ecuador, Special Research Fund of UGent and VLIR-UOS Global Minds. Travelling scholarships for staff and students were provided by FWO, CWO (UGent), VLIR-UOS (Global Minds) and VLIR-UOS Biodiversity Network Ecuador. The funders had no role in study design, data collection and analysis, decision to publish, or preparation of the manuscript.
**Competing Interests**<br><br>Use the instructions below to enter a competing interest statement for this submission. On behalf of all authors, disclose any competing interests that could be perceived to bias this work—acknowledging all financial support and any other relevant financial or non-financial competing interests.<br><br>This statement is required for submission	The authors have declared that no competing interests exist.

and **will appear in the published article** if the submission is accepted. Please make sure it is accurate and that any funding sources listed in your Funding Information later in the submission form are also declared in your Financial Disclosure statement.<br><br>View published research articles from *PLOS ONE* for specific examples.<br><br>**NO authors have competing interests**<br><br>Enter: *The authors have declared that no competing interests exist.*<br><br>**Authors with competing interests**<br><br>Enter competing interest details beginning with this statement:<br><br>*I have read the journal's policy and the authors of this manuscript have the following competing interests: [insert competing interests here]*<br><br>* typeset	
**Ethics Statement**<br><br>Enter an ethics statement for this submission. This statement is required if the study involved:<br><br>• Human participants<br>• Human specimens or tissue<br>• Vertebrate animals or cephalopods<br>• Vertebrate embryos or tissues<br>• Field research<br><br>Write "N/A" if the submission does not require an ethics statement.<br><br>General guidance is provided below. Consult the submission guidelines for detailed instructions. **Make sure that all information entered here is included in the Methods section of the manuscript.**	All methods were carried out in accordance with the relevant guidelines and regulations of the Galapagos National Park Directorate under research permit PC-02-19. All experimental protocols were reviewed and approved by the Galapagos National Park Directorate Applied Research Department, which assesses animal care in research activities.

**Format for specific study types**<br><br>**Human Subject Research (involving human participants and/or tissue)**<br>• Give the name of the institutional review board or ethics committee that approved the study<br>• Include the approval number and/or a statement indicating approval of this research<br>• Indicate the form of consent obtained (written/oral) or the reason that consent was not obtained (e.g. the data were analyzed anonymously)<br><br>**Animal Research (involving vertebrate animals, embryos or tissues)**<br>• Provide the name of the Institutional Animal Care and Use Committee (IACUC) or other relevant ethics board that reviewed the study protocol, and indicate whether they approved this research or granted a formal waiver of ethical approval<br>• Include an approval number if one was obtained<br>• If the study involved *non-human primates*, add *additional details* about animal welfare and steps taken to ameliorate suffering<br>• If anesthesia, euthanasia, or any kind of animal sacrifice is part of the study, include briefly which substances and/or methods were applied<br><br>**Field Research**<br><br>Include the following details if this study involves the collection of plant, animal, or other materials from a natural setting:<br>• Field permit number<br>• Name of the institution or relevant body that granted permission	
**Data Availability**<br><br>Authors are required to make all data underlying the findings described fully available, without restriction, and from the time of publication. PLOS allows rare exceptions to address legal and ethical concerns. See the PLOS Data Policy and FAQ for detailed information.	Yes - all data are fully available without restriction

A Data Availability Statement describing where the data can be found is required at submission. Your answers to this question constitute the Data Availability Statement and **will be published in the article**, if accepted.<br><br>**Important:** Stating 'data available on request from the author' is not sufficient. If your data are only available upon request, select 'No' for the first question and explain your exceptional situation in the text box.<br><br>Do the authors confirm that all data underlying the findings described in their manuscript are fully available without restriction?	
**Describe where the data may be found in full sentences. If you are copying our sample text, replace any instances of XXX with the appropriate details.**<br><br>• If the data are **held or will be held in a public repository**, include URLs, accession numbers or DOIs. If this information will only be available after acceptance, indicate this by ticking the box below. For example: *All XXX files are available from the XXX database (accession number(s) XXX, XXX.).*<br>• If the data are all contained **within the manuscript and/or Supporting Information files**, enter the following: *All relevant data are within the manuscript and its Supporting Information files.*<br>• If neither of these applies but you are able to provide **details of access elsewhere**, with or without limitations, please do so. For example:<br><br>*Data cannot be shared publicly because of [XXX]. Data are available from the XXX Institutional Data Access / Ethics Committee (contact via XXX) for researchers who meet the criteria for access to confidential data.*<br><br>*The data underlying the results presented in the study are available from (include the name of the third party*	The R code, subset of the data and documentation will be made available via Mendeley Data after acceptance of the manuscript: XXX

and contact information or URL).<br>• This text is appropriate if the data are owned by a third party and authors do not have permission to share the data.<br><br>* typeset	
Additional data availability information:	Tick here if the URLs/accession numbers/DOIs will be available only after acceptance of the manuscript for publication so that we can ensure their inclusion before publication.

Cover Letter

Click here to access/download;Manuscript;Optimization_of_a_video_monitorin

[illegible]ampling errors and variability in video transects for assessment of reef fish assemblage structure and [illegible]versity

Video transects for reef fish assemblages

Stijn Bruneel[1,2*], Long Ho[1], Wout Van Echelpoel[1], Amber Schoeters[1], Heleen Raat[1], Tom Moens[2], Rafael Bermudez[3,4], Stijn Luca[5], Peter Goethals[1]

[1] *Department of Animal Sciences and Aquatic Ecology, Ghent University, Coupure Links 653, 9000 Ghent, Belgium,*

[2] *Marine Biology Research Group, Ghent University, Krijgslaan 281, 9000 Ghent, Belgium,*

[3] *Galapagos Marine Research and Exploration, GMaRE. Joint ESPOL-CDF program, Charles Darwin Research Station, 200102 Galapagos Islands, Ecuador*

[4] *Facultad de Ingeniería Marítima y Ciencias del Mar, Escuela Superior Politécnica del Litoral (ESPOL), Campus Gustavo Galindo, 09-01-5863 Guayaquil, Ecuador*

[5] *Department of Data Analysis and Mathematical Modelling, Ghent University, Coupure Links 653, 9000 Ghent, Belgium,*

* *To whom correspondence should be addressed; E-mail: stijn.bruneel@ugent.be*

Abstract

Video monitoring is a rapidly evolving tool in aquatic ecological research because of its non-destructive ability to assess fish assemblages. Nevertheless, methodological considerations of video monitoring techniques are often overlooked, especially in more complex sampling designs, causing inefficient data collection, processing, and interpretation. In this study, we discuss how video transect sampling designs should be assessed and how the inter-observer variability, design errors and sampling variability should be quantified and accounted for. The study took place in the coastal areas of the Galapagos

archipelago and consisted of a hierarchical repeated-observations sampling design with multiple observers.

Although observer bias was negligible for the assessment of fish assemblage structure, diversity and counts of individual species, sampling variability caused by simple counting/detection errors, observer effects and instantaneous fish displacement was often important. Especially for the counts of individual species, sampling variability most often exceeded the variability of the transects and sites. An extensive part of the variability in the fish assemblage structure was explained by the different transects (13%), suggesting that a sufficiently high number of transects is required to account for the within-location variability. Longer transect lengths allowed a better representation of the fish assemblages as sampling variability decreased with 33% if transect length was increased from 10 to 50 meters. However, to increase precision, including more repeats was typically more efficient than using longer transect lengths.

The results confirm the suitability of the technique to study reef fish assemblages, but also highlight the importance of a sound methodological assessment since different biological responses and sampling designs are associated with different levels of sampling variability, precision and ecological relevance. Therefore, besides the direct usefulness of the developed guidelines, the procedures to establish them may be just as valuable for researchers aiming to optimize their own sampling technique and design.

Highlights

- Observer bias is generally limited, but sampling variability is extensive
- Assemblage structure and diversity estimates are more reliable than estimates of abundance
- Species-specific observer effects and detectability are important
- Longer transect lengths improve the representativeness of sampling units
- To improve precision more repeats are preferred over longer transects

Keywords: Video transects, Fish, Experimental design, Inter-observer variability, Design error, Precision

1. Introduction

Anthropogenic pressures are known to affect the physical habitat and chemical water conditions of many reef ecosystems (Halpern et al., 2007). Although biotic indicators, such as the diversity and structure of reef fish assemblages, have proven useful to quantify these environmental and ecological changes (Ford et al., 2018; González-Sansón and Aguilar, 2010; Guillemot et al., 2014), the conclusiveness of any ecological or behavioural field study heavily depends on a proper choice of the sampling method and sampling design. When assessing the abundance, diversity and species composition of fish assemblages, visual census has been the preferred non-destructive method,

with stationary point counts and strip transects being the most common options (Caldwell et al., 2016; Denny and Willis, 2003; Jones et al., 2015). As such methods require substantial training and considerable time in the field, the number of locations that can be sampled is limited (Thompson and Mapstone, 1997). In addition, such traditional visual census techniques are often characterized by high detection heterogeneity, caused by observer bias and observer effects (Dickens et al., 2011; Langlois et al., 2010; Lindfield et al., 2014; Pais and Cabral, 2017; Willis et al., 2000; Willis, 2001). ~~In literature,~~ observer effects are often considered as the spatiotemporal variation in observer bias due to gained experience or replacement of observers (Thompson and Mapstone, 1997). We argue, however, that the proceeding of sampling through time and space will not only alter the perception of the environment, but also the environment itself. More specifically, observers might attract or deter species, while also gaining more experience and an altered perception. Therefore, although partly related, observer bias is considered the result of consistent differences in the perception of a unique set of observers, while observer effects are considered the result of differences in observers' perception through time and space in combination with fish species-specific traits such as detectability and response to presence of observers. These observer effects in combination with random counting/detection errors and "random" or instantaneous fish displacement (see Appendix A.1) at very short time scales (e.g. minutes) constitute the sampling variability which is referred to as baseline or instantaneous variability in observational fish assemblage studies.

This sampling variability should be accounted for (or at least acknowledged) when designing sampling schemes and interpreting data (McClanahan et al., 2007; Irigoyen et al., 2013).

Nowadays, stationary video cameras are often used to remove observer bias, to reduce the impact on fish behavior and to be able to store the video data for future studies and validation (Cappo et al., 2006, 2011; Schmid et al., 2017). Nevertheless, stationary cameras only provide a limited view of the study area as only small patches are monitored, and are therefore of limited use when an extensive survey of a study area has to be done. Because fish species, and in particular reef fish, are often strongly associated with specific micro-habitats (Chapman and Kramer, 2000), a more mobile approach may be appropriate for more cost-effective monitoring. Video transects still suffer from observer bias and observer effects, but the possibility to store video data for later analysis, to reduce the amount of time spent on field work and to standardize observations, are important incentives to upgrade traditional visual census techniques. Video transects have already been applied in recent studies (Mallet and Pelletier, 2014; Wartenberg and Booth, 2015), and remotely operated vehicles (ROVs) are likely to soon replace camera-operating humans in a variety of applications (Schramm et al., 2020; Sward et al., 2019). Although studies comparing different video and other visual census techniques are becoming more frequent (Andradi-Brown et al., 2016; Langlois et al., 2010; Tessier et al., 2013; Watson et al., 2005, 2010; Wilson et al., 2018), few have focused on the methodological aspects of video tran-

sects, such as observer bias, design errors and sampling variability (Goetze et al., 2019). Video transects are often considered a simple extension of visual census transects, although the ability to store and standardize video observations (see Appendix A.2) justifies a specific methodological assessment of the technique.

On the one hand, applied ecological studies aiming to understand ecological processes and how they are affected by management practices typically make use of multiple random transects within one location to obtain a general picture of the local fish assemblage, each transect being covered only once by a single observer (Denny and Willis, 2003; Labrosse et al., 2002; Ruttenberg et al., 2005). The lack of repeated observations renders any assessment of the variability related to observers, transects and methodological parameters difficult (Katsanevakis et al., 2012). On the other hand, applied studies are often characterized by complex hierarchical designs, while methodological studies typically entail more simple sampling designs (Bijoux et al., 2013; Dickens et al., 2011; Watson et al., 2005), introducing a conceptual mismatch between both. Besides differences in the sampling design, there are often also differences in the statistical tools used by both types of studies. While applied studies typically involve either multivariate (for fish assemblages) and/or univariate (for single species) models (Andradi-Brown et al., 2016; Denny and Willis, 2003; Samoilys and Carlos, 2000; Zintzen et al., 2012), methodological studies generally apply the latter (Pelletier et al., 2012; Edgar et al., 2004; Langlois et al., 2010; Thompson and Mapstone, 1997), but there are

exceptions which integrate both (Cappo et al., 2011; Gladstone et al., 2012; Lindfield et al., 2014).

To ensure a close connection between methodology and application while maintaining a sound statistical basis to assess the importance of different factors, the present study laid out a hierarchical design of repeated fixed video transects, with each transect being covered multiple times by multiple observers. The sampling design allowed us to assess the suitability of video transects to study reef fish assemblages and to set up some practical guidelines. Although repetitions of transects are not common in literature, they were necessary here to provide answers to our research questions, which are of interest for both non-repetitive and repetitive studies.

- Are there (species-specific) observer effects which cause dependence between observations?
- How important are observer bias and sampling variability?
- How important are the methodological parameters transect length, number of repeats, counting metric and data transformation?

2. Material and methods

2.1. Data collection

The study took place in the Galapagos archipelago as part of an extensive project to map and monitor the unique, yet fragile, coastal ecosystems

Glossary	
Design error	Error associated with the methodological characteristics of the sampling technique (e.g. transect length, swimming speed)
Detectability	Probability of observing a particular species during a given sampling occasion conditional on its presence at that location (Kulbicki, 1998; MacNeil et al., 2008b)
Instantaneous variability	Sampling variability caused by the instantaneous displacement of fish, observer effects and random counting/detection errors (Irigoyen et al., 2013; McClanahan et al., 2007)
Observer bias	Effect of consistent differences in the perception of a unique set of observers on fish count
Observer effects	The effect of differences in observers' perception through time and space in combination with fish species-specific traits such as detectability and response to presence of observers on fish count

of the islands. At ten locations, fixed transects were used to assess reef fish assemblages in rocky habitats close to the coast. The locations ~~(n=10)~~ were situated along the coasts of two cities of two different islands; Puerto Ayora (Academy Bay) on Santa Cruz island (n=5) and Puerto Velazco Ibarra on Floreana island (n=5). The fish data collection was based on a standard operation procedure developed by the Aquatic Ecology Research Group of Ghent University. In each location, 3 transects with a length of 50 meters each were laid out using ropes. For fish to be included they had be recorded within 2.5 m of either side of the transect line (area = 50 x 5 = 250 m^2). Observers were trained to recognize whether fish had to be considered, depending on the estimated distance from the rope. All transects were monitored at a constant depth of 1.5 meter, parallel to the coastline, and only locations with a limited exposure to waves were selected to guarantee the safety of the observers and to avoid predominant effects of wave exposure on the structure of fish assemblages. All transects were approximately 20 meters apart. To

account for observer bias and sampling variability, each of these transects was recorded six consecutive times with single GoPro cameras (GoPro Hero 5 Black, 1080p, 60fps, wide FOV) by three different observers equipped with a mask and snorkel. Hence, 2 islands x 5 locations x 3 transects x 3 observers x 6 repeats yielded 540 observations (Fig. 1).

The observers covered the transects in a browsing fashion, similar to the S-type transects introduced by Pelletier et al. (2011). Observers browse through the transect at a fixed speed, but varying angle and can zoom in if needed to find individuals hiding in crevices. This S-transect was chosen over the more standarized I-transect with fixed angle and without zooming to enable detection of more individuals and species (Pelletier et al., 2011). Transects were placed during low tide and monitoring occurred during flood tides of consecutive days from the 19th until the 31st of August 2017. The duration of each observation was approximately 4.6 ~~minutes, but varied (~~ ± 0.7 minutes). The time between successive observations was at least one minute. The analysis of the videos included species identification and the estimation of the number of individuals per species. Each fish identification and count was labeled with a time stamp to allow later splitting of the data in different subsets. Fish were counted in such a way that both MaxCount (total number of individuals per species) and MinCount (maximum number of individuals per species in one frame, also referred to as MaxN) could be determined (see section Appendix B). The videos were randomly assigned to one of two video analysts to reduce any potential experience bias from improving identification

skills (Williams et al., 2006). Each video was only analyzed once. Each observation was split up in 5 distance intervals (0-50, 5-45, 10-40, 15-35, 20-30 meters), based on the time stamps of the fish identifications, to assess the added value of increasing transect length. The exact distance could not be determined, but the observers aimed to maintain a constant swimming speed, so distance could be estimated from the time stamps.

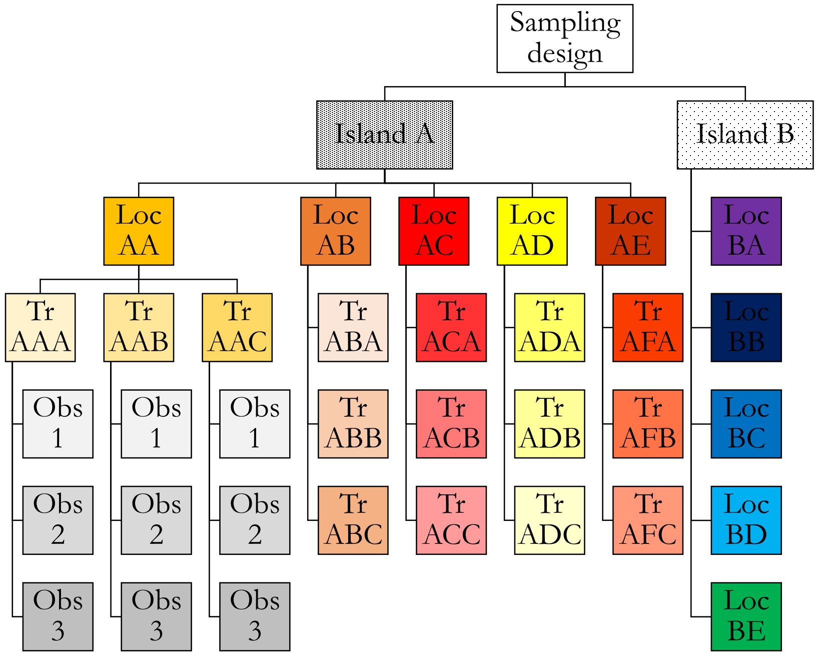

Figure 1: Sampling design of the study. Three spatial nested levels can be identified: Island, Location (Loc) and Transect (Tr). Each observer (Obs) covered each transect six times. For clarity reasons, not all subdivisions are presented in this figure. All transects of the different locations of island A were covered by the three observers and the locations of island B were further subdivided in transects, each covered by all three observers. The sampling design was balanced.

2.2. Data analysis

To understand patterns in the data, models for variation partitioning were developed. For this models to be relevant for methodological assessments, it is important to translate model results back to the context of data collection and sampling design (Fig. 2). Although ideally the inter-observer variability of our models would only contain the observer bias, the potential temporal dependence of observations over observers might cause the estimates to be inflated by the variability caused by observer effects and instantaneous fish displacement. Indeed, because observers covered transects sequentially the disturbances caused by the first observer might affect the observations of the next observers. Furthermore, it is likely that in case an individual fish was recorded during a certain observation, it might still be there during the subsequent observation because it is foraging or hiding for predators for a specific period of time. If this period of residence is longer than the time lapse between observations, the chance that a specific observer encounters the species after it was observed during the previous observation becomes inflated (this inflated chance is the result of the non-randomness of instantaneous fish displacement). The remaining sampling variability is contained within the residual variability.

To assess the independence of successive observations and to evaluate the importance of observer effects, auto-correlation functions and binomial models were developed (section 2.2.1). To quantify sampling variability and to evaluate the methodological parameters, models allowing to assess univari-

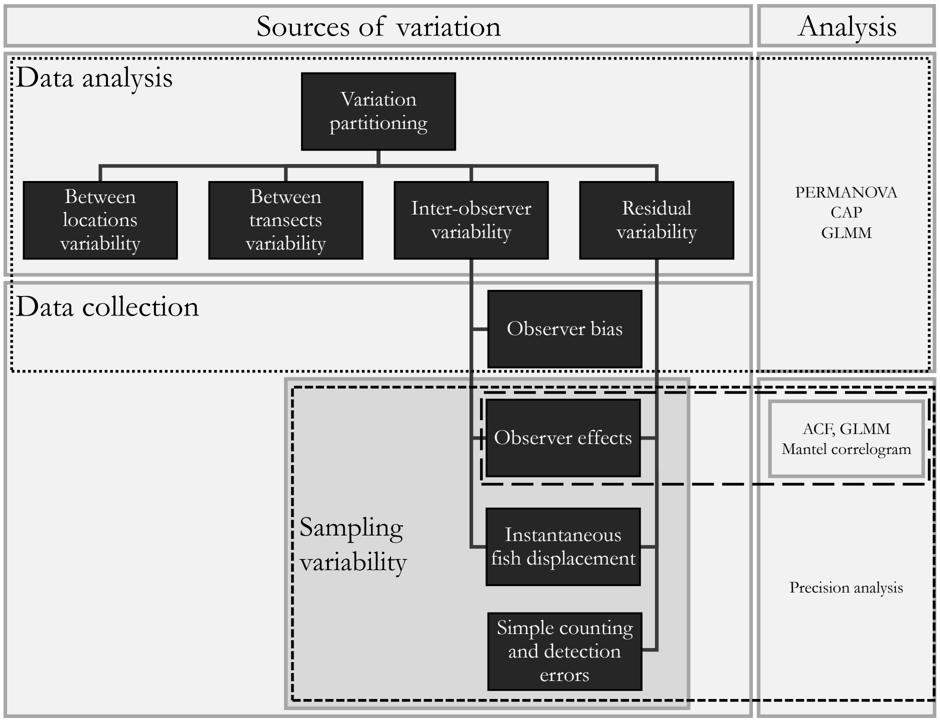

Figure 2: Methodological framework for the study. Variation partitioning using PERMANOVA (permutational multivariate analysis of variance), CAP (canonical analysis of principal components), and GLMM (generalized linear mixed models) to quantify the different sources of variation for multivariate and univariate data respectively. The modeled inter-observer variability not only comprises the observer bias, but also sampling variability, i.e. observer effects and instantaneous fish displacement. The residual variability comprises the remaining sampling variability. ACF (auto-correlation functions), GLMM and partial Mantel correlograms to assess observer effects. Precision analysis was used to assess the effect of number of repeats and transect length on quantification and reduction of sampling variability.

ate (section 2.2.3) and multivariate variability (section 2.2.4) were developed. Transect length and number of repeats were evaluated through assessment of the design error (section 2.2.2) and precision estimates (section 2.2.5). Since independence and design error (section 2.2.1 and 2.2.2) need to be considered in model development, they were evaluated prior to the assessments of

univariate and multivariate variability and precision (section 2.2.3, 2.2.4 and 2.2.5).

All analyses were performed using the R software (version 3.6.2, R Developer Core Team, R Foundation for Statistical Computing, Vienna, Austria) and the Primer v6 multivariate statistics package (Clarke and Gorley, 2006) with PERMANOVA add-on (Anderson et al., 2008).

2.2.1. Independence of observations and observer effects

To assess whether successive observations within the same transect were independent, the auto-correlation functions (ACF; $r_{k,j}$) of the counts of each species of the 18 successive observations ($Y_{1,j}$, $Y_{2,j}$, ..., $Y_{i,j}$, ..., $Y_{18,j}$) per transect j for lag k, were determined,

$$\overline{r_{k,j}} = \frac{\sum_{i=1}^{18-k}(Y_{i,j} - \overline{Y_j})(Y_{i+k,j} - \overline{Y_j})}{\sum_{i=1}^{18}(Y_{i,j} - \overline{Y_j})^2} \tag{1}$$

In addition, for each observation lag k, the average ACF of the count of each species over the transects was determined, providing detailed information on the temporal scale at which repeated observations exhibited interdependence. Finally, to assess whether individual species were affected in their behavior by the presence of an observer, binomial mixed models were constructed (Linearity between predictors and the logit of the outcomes was evaluated using scatter plots). Models were constructed for the 18 successive observations per transect and for the 6 successive observations per observer and per transect. The binomial mixed models had the Presence/Absence of

the concerning species ($Y'_{1,j}$, $Y'_{2,j}$, ..., $Y'_{18,j}$) as response, Order or sequence number of the observation i as a fixed effect and transect j as a random effect.

$$\begin{aligned} Y'_{i,j} &\sim Binomial(n_{i,j}, p_{i,j}) \\ \log\left(\frac{p_{i,j}}{1-p_{i,j}}\right) &= \alpha + \beta \times Order_{i,j} + b_j \times Transect_j \end{aligned} \quad (2)$$

Species which were only observed during the first of 18 observations were likely scared off by observers, while species which were only observed during the last observations were likely attracted by the observers. Patterns for single observers could indicate that observers specifically targeted or avoided areas where species were found earlier. For these analyses, all series of observations which had at least one observed presence of the species concerned were considered. The level of independence of the observed structure of fish assemblages was assessed using a partial Mantel correlogram (Legendre and Legendre, 2012). The correlation between (1) the number of repeats/lags between observations and (2) the Bray-Curtis dissimilarity values was determined while partialling out the effect the sampling unit (i.e. transect) and observer might have had (categorical variable included as matrix of dummy variables).

2.2.2. Design error

Observed absences (zeros) often have multiple origins: True zeros may either be structural, i.e. related to habitat unsuitability, environmental conditions and/or dispersal limitations; or they can be "random", i.e. related to sampling variability. False zeros may be related to counting errors, observer

effects or errors in the experimental design (Blasco-Moreno et al., 2019). An important design error may arise from the choice of transect length. If transect lengths are too short, many observations may contain no species at all and there may also be pairs of observations having no or only few species in common, leading to uninformative dissimilarity values and questionable multivariate analyses (Clarke et al., 2006). In those cases, lack of information within individual observations may require pooling sets of observations (Anderson and Santana-Garcon, 2015). Therefore, to assess whether the transects were long enough to ensure that observations could be treated as solid repeats rather than subsamples, Monte Carlo simulations were performed during which percentage difference (Bray-Curtis) dissimilarity matrices were calculated (Anderson and Santana-Garcon, 2015). Permutation scenarios (10^4 permutations) included the random pooling of 1 to 6 or 1 to 18 observations (mean) for different transect lengths and over two different spatial factors (i.e. Transects and Locations, respectively). The permutations and the calculation of the dissimilarity values were obtained within each level of the considered factor, allowing to assess the dissimilarity in recorded fish assemblages among different subsets of pooled observations for a given Transect or Location. Since there were 30 transects and 10 locations, 30 and 10 series of 10^4 permutations each were conducted. Finally, the average frequency distributions of the dissimilarity values were calculated, to assess the overall trend in dissimilarity with increasing transect length and number of pooled observations.

2.2.3. *Variability in abundance and diversity estimates*

The importance of the different grouping factors (Location, Transect and Observer) for the estimates of species density and Shannon diversity were assessed using linear mixed models. To relate the counts (MaxCount) of individual species to the different factors, zero-inflated generalized linear mixed models (GLMM) were developed. The output of models with Conway-Maxwell-Poisson (COM-Poisson) distribution with log-link were reported. The COM-Poisson distribution generalizes the Poisson distribution by adding a parameter to model both underdispersion and overdispersion (Lynch et al., 2014), while the negative binomial only accounts for overdispersion (Imoto, 2014). Since one-third of the species distributions were underdispersed and two-thirds overdispersed, the COM-Poisson distribution seemed most suitable. In addition, the count COM-Poisson models outperformed, based on AIC, count models using a Poisson or negative binomial distribution for most species. A subset of four species, with the potential to serve as indicator species for differences in the structure of fish assemblages, were selected to provide a more in-depth assessment (for a more detailed description, see Appendix D). Both types of models had Island as fixed effect, Location and Transect as nested random effects and Observer as a crossed random effect. The Intraclass Correlation Coefficient (ICC) was estimated to quantify the proportion of variance explained by the different random factors (Nakagawa

and Schielzeth, 2017),

$$
\begin{aligned}
&ICC_r = \frac{\sigma_r^2}{\sum \sigma_r^2 + \sigma_\varepsilon^2} \\
&ICC_{Total} = \frac{\sum \sigma_r^2}{\sum \sigma_r^2 + \sigma_\varepsilon^2} \\
&ICC_\varepsilon = \frac{\sigma_\varepsilon^2}{\sum \sigma_r^2 + \sigma_\varepsilon^2} = 1 - ICC_{Total} \\
&ICC_{Sampling} = ICC_\varepsilon + ICC_{Observer:Transect} + ICC_{Observer:Location}
\end{aligned} \tag{3}
$$

with σ_r^2 the variance of random factor r and σ_ε^2 the residual variance. ICC$_\varepsilon$ measures the proportion of unexplained variability, while ICC$_{Sampling}$ measures the proportion of sampling variability. The lmer and glmmTMB R packages were used to develop the linear and generalized linear mixed models, respectively (Brooks et al., 2017).

2.2.4. Variability in the structure of fish assemblages

The observed fish assemblages (MaxCount) were assessed using PERMANOVA models (10^4 permutations under a reduced model). Data was fourth-root transformed and Bray-Curtis dissimilarities were calculated. The models had Island as fixed factor, Location and Transect as nested random factors and Observer as crossed random factor. The addition of the factor Video Analyst was evaluated separately because of its unbalanced nature. The square root components of variation (σ') were estimated by equating the mean squares of the PERMANOVA models to their expectations (Anderson et al., 2008). To determine the proportion of the variability explained by the different grouping factors, the percentage of variation of each com-

ponent to the total variation was used (correction for different degrees of freedom among grouping factors). The combined contribution of $\sigma'_{Residuals}$, $\sigma'_{Observer:Location}$ and $\sigma'_{Observer:Transect}$ was used as a proxy of the proportion of sampling variability (see section 2.2.3). R^2 values (ratio of sum of squares of the grouping factors over the total sum of squares: no correction for different degrees of freedom among grouping factors) were estimated to assess the effect of longer transect lengths, different metrics (MinCount and MaxCount), data transformations (fourth root, logarithm and presence/absence) and dissimilarity indices (Bray-Curtis, Euclidean, Gower and Kulczynski) on the goodness-of-fit of the PERMANOVA models. To evaluate the performance of a specific methodological parameter, all possible combinations of parameter values were tested, yielding 120 PERMANOVA models. CAP (Canonical Analysis of Principal components) was applied over the different transect lengths to assess the distinctiveness of the different Locations and Transects using the classification error after leave-one-out cross-validation (Anderson and Willis, 2003).

2.2.5. Precision estimates of abundance, diversity and fish assemblage structure

Precision for both the univariate, i.e. abundance and diversity, and multivariate, i.e. fish assemblage structure, variables was estimated to determine the required number of repeats and required transect length to account for sampling variability. Univariate precision was determined as the inverse of

the standard error (of the mean) to mean ratio (Andrew and Mapstone, 1987). The precision for species density, Shannon diversity and count (MaxCount) of the observed species was determined using permutations (n=10^4) over the transects using the 18 observations per transect (Manly, 1992). To compare the effect of more repeats and longer transect lengths, the precision was assessed as a function of the total swim distance, determined as the number of repeats multiplied by the transect length. The effect of turbidity on the precision of the species density and Shannon diversity was assessed after classifying the 30 transects in three equally sized groups based on the rank-order of the level of visually graded turbidity.

For the multivariate analysis, the square root of the residual mean square of the PERMANOVA models divided by the number of repeats was used as a measure of multivariate pseudo error variance (multSE) (Anderson and Santana-Garcon, 2015). This proxy of multivariate precision was evaluated against transect length and number of repeats. For each transect length, permutations (n=10^4) over the transects using the 18 observations per transect were applied to assess the effect of different numbers of repeats.

3. Results

3.1. Independence of observations and observer effects

Although some evidence of temporal auto-correlation was found, this was only significant for two species (p<0.05), the Harlequin wrasse (*Bodianus eclancheri*) and the Pacific dog snapper (*Lutjanus novemfasciatus*) (Table C.1). The order of the observations, determined using all 18 observations of each transect (Table C.2; mixed binomial models), had a significant negative effect on the probability of observing the Cortez rainbow wrasse (*Thalassoma lucasanum*, p<0.001), Marbled goby (*Gobio manchada*, p=0.015) and Stone scorpion fish (*Scorpaena mystes*, p=0.006), suggesting that these fish species were scared away by observers. By contrast, the order of the observations had a significant positive effect on the probability of observing the Amarillo snapper (*Lutjanus argentiventris*, p=0.048), Galapagos triplefin blenny (*Lepidonectes corallicola*, p=0.001), Mexican hogfish (*Bodianus diplotaenia*, p=0.007), Panamic sergeant major (*Abudefduf troschelii*, p<0.001) and White salema (*Xenichthys agassizii*, p=0.034), suggesting that these fish species were attracted by the observers. For the mixed binomial models using all 6 observations of each transect per observer, the order of the observations only had a significant positive effect on the probability of observing the Panamic sergeant major (*Abudefduf troschelii*, p=0.007) and Reef cornet fish (*Fistularia commersonii*, p=0.048) and a marginally positive effect on the Bravo clinid (*Gobioclinus dendriticus*) (p=0.078). The order of the observations had a significant negative effect on the probabil-

ity of observing the Blue and gold snapper (*Lutjanus viridis*, p=0.024) and Yellowtail damselfish (*Chrysiptera parasema*, p=0.001). The partial Mantel correlogram indicated that temporal dependencies in the structure of fish communities were not significant (p<0.001), suggesting that observations could be treated as independent.

3.2. Design error

Pooling observations had a clear effect on the frequency distributions of the dissimilarity values for both spatial factors, Transect and Location, and for all different transect lengths (10 to 50 meters) (Fig. 3). As expected, the observations became more similar (lower dissimilarity values) when pooling observations, a trend which was most pronounced for the longer transect lengths (Fig. 3). However, even at transects of 10 meters, hardly any uninformative dissimilarity values were obtained (Table G.1 and G.2). Therefore, there was no indication that the observations had to be pooled and the original observations could be used for the multivariate models. The higher number of lower dissimilarity values when pooling within Transect compared to pooling within Location was expected, since the repeated observations of a single sampling unit (Transect) are likely to be more similar than the observations of two different sampling units within the same location. Because of the clear difference between transects of the same location, it was decided to only determine the precision within Transect (over the different observers) and not within Location. Hence, per response variable, 30 (=number of

396 transects) precision curves could be constructed (see section 3.5).

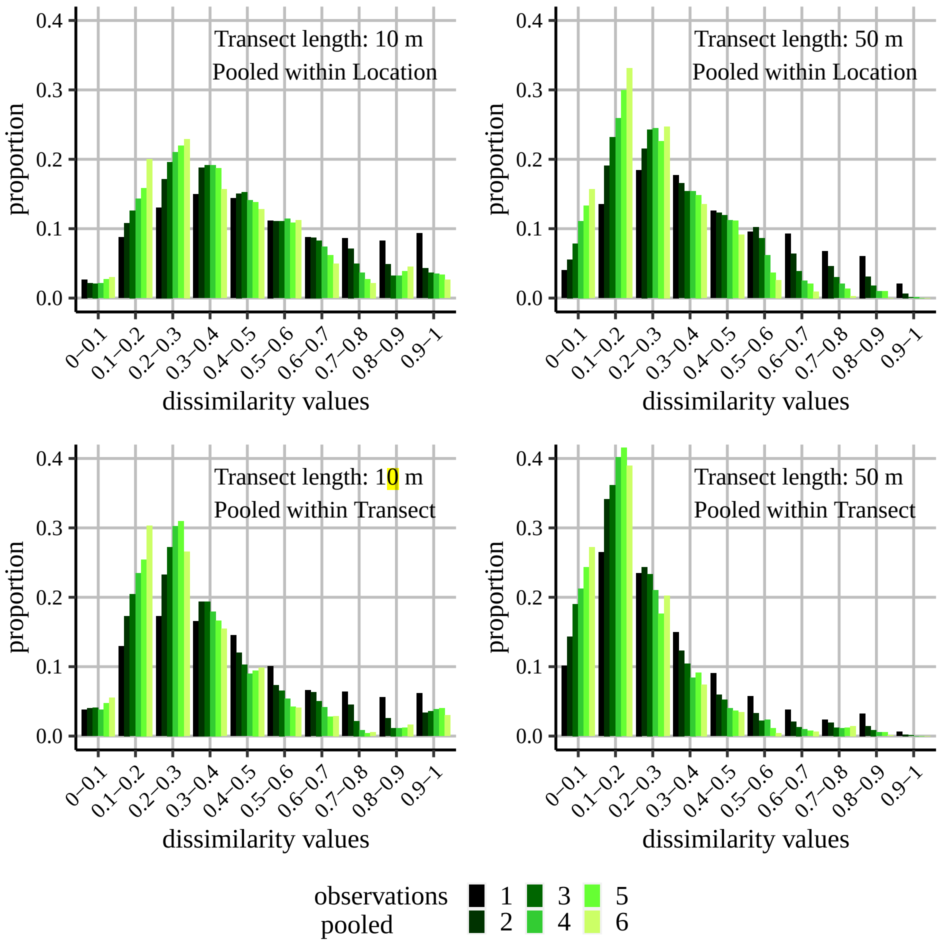

Figure 3: Histograms representing the frequency distributions of dissimilarity values (fourth-root transformed, Bray-Curtis) for different pooling scenarios. Low dissimilarity values indicate that observations are relatively similar. Different spatial scales (Location versus Transect), Transect lengths (10 versus 50 meters) and number of observations to be pooled (1 to 6) were assessed. As observations within transects and locations are compared, we would expect relatively low dissimilarity values.

3.3. *Variability in abundance and diversity estimates*

The ICC_{Total}, representing the proportion of the variability of the species density and Shannon diversity explained by the factors Location, Transect, Observer and their interaction increased with increasing transect length (Table 1). For both the species density and Shannon diversity the proportion of sampling variability decreased with increasing transect length. For species density, there was a slight decrease in the proportion of variability explained by Location, but this was compensated by the increase in the proportion of variability explained by Transect. The factors Observer and interaction Observer:Location explained none or only little of the variability of the species density, but the proportion of variability explained by the interaction Observer:Transect was relatively high. For a transect length of 10 and 20 meters it was even higher than the proportion of variability explained by Transect.

Similarly, the proportion of variability of the Shannon diversity explained by Transect increased with increasing transect length (Table 2). The proportion of variability explained by Location decreased rapidly, while the proportion of variability explained by the interactions Observer:Transect and Observer:Location showed a clear increase. The latter were even higher than the proportion of variability explained by Location from transect lengths 20 to 50 meters.

The proportion of variability of the counts of the potential indicator species explained by the factors Location, Transect, Observer and their interaction did not show any consistent trend with increasing transect length

	10 meters	20 meters	30 meters	40 meters	50 meters
$\mathrm{ICC}_{Transect}$	0.073	0.116	0.145	0.180	0.204
$\mathrm{ICC}_{Location}$	0.369	0.354	0.345	0.351	0.345
$\mathrm{ICC}_{Observer}$	0.000	0.003	0.010	0.010	0.007
$\mathrm{ICC}_{Observer:Transect}$	0.103	0.131	0.092	0.112	0.099
$\mathrm{ICC}_{Observer:Location}$	0.015	0.000	0.004	0.000	0.007
ICC_{Total}	0.560	0.604	0.596	0.653	0.662
$\mathrm{ICC}_{Sampling}$	0.558	0.527	0.500	0.459	0.444

Table 1: Model output (ICC (Intraclass Correlation Coefficient) of the random effects, total ICC explained by the factors of the model and ICC associated with the sampling variability) for a linear mixed model with species density as response, Island as fixed effect, Location and Transect as nested random effects and Observer as crossed random effect. Transect lengths of 10, 20, 30, 40 and 50 meters were assessed.

	10 meters	20 meters	30 meters	40 meters	50 meters
$\mathrm{ICC}_{Transect}$	0.184	0.261	0.380	0.420	0.442
$\mathrm{ICC}_{Location}$	0.104	0.044	0.000	0.000	0.000
$\mathrm{ICC}_{Observer}$	0.001	0.000	0.009	0.008	0.010
$\mathrm{ICC}_{Observer:Transect}$	0.082	0.119	0.066	0.078	0.161
$\mathrm{ICC}_{Observer:Location}$	0.065	0.081	0.081	0.110	0.105
ICC_{Total}	0.436	0.505	0.536	0.616	0.718
$\mathrm{ICC}_{Sampling}$	0.711	0.695	0.611	0.572	0.548

Table 2: Model output (ICC (Intraclass Correlation Coefficient) of the random effects, total ICC explained by the factors of the model and ICC associated with the sampling variability) for a linear mixed model with Shannon diversity as response, Island as fixed effect, Location and Transect as nested random effects and Observer as crossed random effect. Transect lengths of 10, 20, 30, 40 and 50 meters were assessed.

(Table F.1). The proportion of variability explained by Observer (i.e. observer bias) was lower than the proportion of variability explained by Transect and Location for 34 out of 36 species. However, interaction effects of Observer with either Transect or Location were often important as the proportion of variability explained by the interactions Observer:Transect and Observer:Location, exceeded the proportion of variability explained by Transect and Location for 18 out of 36 species. For 28 out of 36 species the sampling variability exceeded the variability explained by Transect and Location (Table F.2).

3.4. Variability in the structure of fish assemblages

For all different transect lengths, the factors Island, Location and Transect explained a larger amount of the observed variability than the factor Observer and its interactions (largest proportion of σ'^2: estimates of components of variation) in the PERMANOVA models (10^4 permutations) (Table 3). The factor Observer only explained a limited proportion of the variability (between 0 and 0.3 %). However, a relatively large part of the variability (between 0.9 and 4.4 %) was explained by the interactions of Observer with Location and Observer with Transect. With increasing transect length the proportion of sampling variability decreased (from 25.9 to 17.4 %), while the proportion of variability explained by the factors Location and Transect increased (from 8.2 to 12.5 % and from 10.0 to 12.8 % respectively). Inclusion of the factor Video Analyst had a non-significant and negligible effect (Table

	10 meters	20 meters	30 meters	40 meters	50 meters
σ'_{Island}	**45.70	**43.13	**41.66	**40.21	**39.39
$\sigma'_{Observer}$	3.17	-0.42	1.99	2.31	2.72
$\sigma'_{Location}$	**17.58	**17.49	**17.47	**18.02	**18.41
$\sigma'_{Island:Observer}$	1.40	2.82	2.37	2.46	1.25
$\sigma'_{Transect}$	**19.35	**19.60	**19.54	**19.27	**18.61
$\sigma'_{Location:Observer}$	*5.84	**8.34	**8.14	**8.18	**8.98
$\sigma'_{Transect:Observer}$	**11.90	**12.34	**11.53	**11.15	**10.92
$\sigma'_{Residuals}$	28.19	23.28	20.66	18.58	16.49
% σ'^2_{Island}	55.64	56.00	56.76	56.52	57.09
% $\sigma'^2_{Observer}$	0.27	0.00	0.13	0.19	0.27
% $\sigma'^2_{Location}$	8.23	9.21	9.98	11.35	12.48
% $\sigma'^2_{Island:Observer}$	0.05	0.24	0.18	0.21	0.06
% $\sigma'^2_{Transect}$	9.97	11.57	12.48	12.98	12.75
% $\sigma'^2_{Location:Observer}$	0.91	2.09	2.17	2.34	2.97
% $\sigma'^2_{Transect:Observer}$	3.77	4.59	4.34	4.34	4.40
% $\sigma'^2_{Residuals}$	21.16	16.31	13.95	12.07	10.01
% $\sigma'^2_{Sampling}$	25.89	23.23	20.64	18.96	17.44

Table 3: Square root estimates of components of variation (σ') and the percentage of variation of each component to the total variation of PERMANOVA models based on Bray-Curtis dissimilarities (fourth-root transformed) with Island as fixed factor, Location and Transect as nested random factors and Observer as crossed random factor for transect lengths of 10, 20, 30, 40 and 50 meters.*$p<0.05$, **$p<0.01$

H.2).

Using different transect lengths, metrics, data transformations and methods to calculate dissimilarity matrices had a significant ($p<0.05$) effect on the goodness-of-fit of the PERMANOVA models (pairwise paired t-test of R^2 values of each level of every considered factor with Bonferroni correction. Interactions were not considered). The most significant differences were due to the increase of the transect length followed by the method to calculate the dissimilarity matrices (Bray-Curtis, Euclidean, Gower and Kulczynski),

	Leave-one-out cross-validation classification error				
	10 meters	20 meters	30 meters	40 meters	50 meters
Location	35.37	36.11	28.70	25.56	25.00
Transect	60.37	52.78	46.85	42.04	36.85

Table 4: Classification error of leave-one-out cross-validation of CAP analyses (Bray-Curtis, fourth-root transformed, eight PCO axes (m)). CAP analyses were performed for different transect lengths (10, 20, 30, 40 and 50 meters) and different grouping factors (Location and Transect).

the metrics (MaxCount and MinCount) and finally the data transformation (fourth-root, logarithm and presence/absence) (Figs H.2 and H.1).

The leave-one-out cross-validation classification error of the CAP analysis decreased with increasing transect length for both the grouping factors Transect and Location (Table 4). The decrease was most pronounced for the analysis using Transect as grouping factor.

3.5. Precision estimates of abundance, diversity and fish assemblage structure

Precision, defined as the inverse of the standard error over the mean, of our estimates of both species density and Shannon diversity increased with increasing number of observations and increasing transect length (Fig. 4, A and C). Although the precision improved markedly with increasing total swim distance (transect length x number of repeats), it did not matter much whether more repeats or longer transects were considered: 10-meter transects were slightly more efficient to obtain precise results on the species density (Fig. 4 B), while 20 or 30-meter transects were slightly more efficient for the Shannon diversity (Fig. 4 D). For both diversity measures, the preci-

sion seemed to level off at approximately 250 meters of total swim distance. Higher levels of turbidity caused lower levels of precision for both the species density and Shannon diversity (Fig. I.1). For all observed species, shorter transects (10 and 20 meters) with more repeats turned out to be most efficient to improve precision in terms of total swim distance, with the exception of the Galapagos Sea Bream (*Archosargus pourtalesi*) and the Black Striped Salema (*Xenocys jessiae*), for which longer transect lengths and less repeats were more efficient (Fig. I.2). The stronger effect of repeats over transect lengths was more clear for the counts of the individual species than for the diversity indices, but the total swim distance at which the precision curve levelled off was highly species dependent. Multivariate pseudo error variance (MultSE) was also clearly affected more strongly by the number of repeats than by transect length, hence choosing 10-meter transects turned out to be most efficient to improve precision (Fig. 5). MultSE leveled off at longer total swim distances for the longer transect lengths; for example, for 50-meter transects this was around 350 meters, while for 20-meter transects this was around 200 meters.

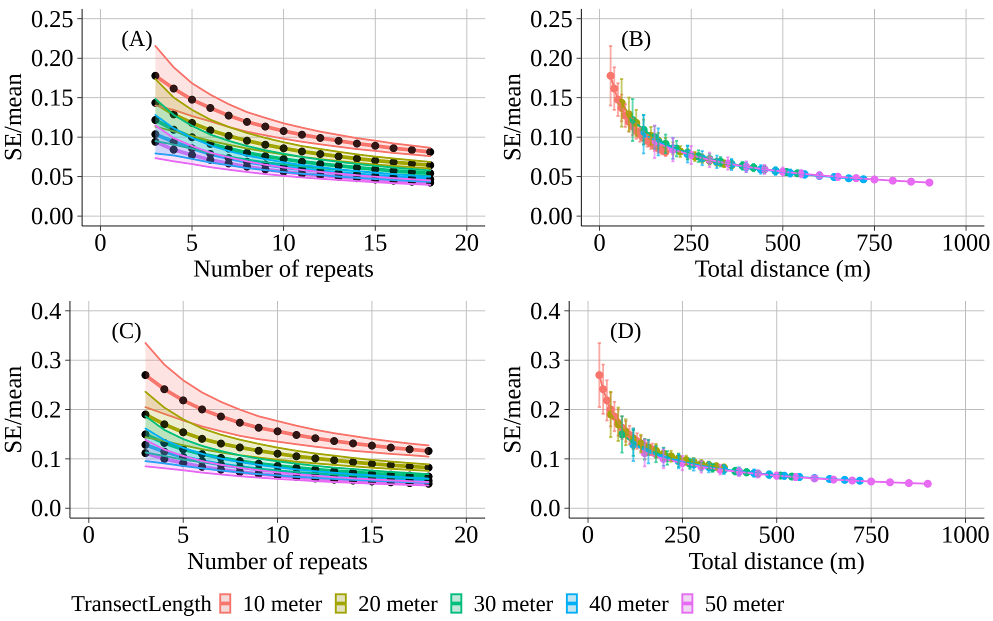

Figure 4: Estimates of the standard error over the mean for species density (A) and Shannon diversity (C) in function of the number of repeats. Precision is defined as the inverse of the standard error over the mean. In (B) and (D) the standard error over the mean of species density and Shannon diversity is given as a function of the total swim distance. Total swim distance is defined as the number of repeats multiplied by the transect length. Different transect lengths were considered ranging from 10, 20, 30, 40 to 50 meters. Monte Carlo simulations (n=10^4) were applied to determine the standard error over the mean per transect. The average values over all transects are visualized. The error bars represent the 95% confidence intervals which were constructed using the pooled standard deviation of the estimates.

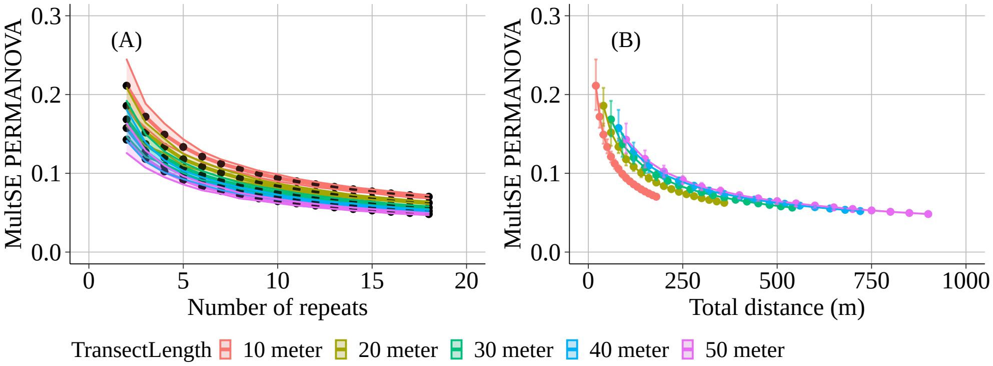

Figure 5: Multivariate pseudo error variance (multSE) of PERMANOVA analysis based on Bray-Curtis dissimilarities (fourth-root transformed) with Island as fixed factor and Location and Transect as nested random factors. Monte Carlo simulations (n=10^4) were applied to determine the multSE per number of repeats. The average values over all transects are visualized. The error bars represent the 95% confidence intervals which were constructed using the pooled standard deviation of the estimates.

4. Discussion

4.1. Observer bias, observer effects and instantaneous fish displacement

Observer biases and sampling variability are important aspects to consider in observational studies of fish assemblages, but the simple sampling designs of most studies hampers a detailed assessment (Bernard et al., 2013; Murphy and Jenkins, 2010). The hierarchical sampling design of this study allowed to estimate inter-observer variability related to the observers themselves (i.e. observer bias) and their interaction with the sampling units and sites (i.e. observer effects and instantaneous fish displacement). Although the provided estimates of the observer bias and sampling variability can be considered as relatively reliable, the additional partitioning of sampling variability in observer effects, instantaneous fish displacement and random counting/detection errors is subjected to much more uncertainty. Although fish displacement at very short time scales is often referred to as random, perfect randomness seldomly occurs in ecology and undetected spatiotemporal patterns are more likely the rule than the exception (e.g. undocumented foraging and reproduction behavior and the conditional nature of animal movement in terms of space and time) (Dornelas et al., 2006). Since patterns related to observer effects and instantaneous fish displacement can therefore be confounded and difficult to unravel, additional insights in the biological and ecological traits of fish can be useful to distinguish between causes of variation.

The relatively low $ICC_{Observer}$ values, which are an indication of the pro-

portion of variability explained by the factor Observer, of most univariate models in combination with the low $\sigma'_{Observer}$ of the multivariate models suggested that systematic observer biases were generally limited. However, the interaction between the factor Observer and the factor Transect or Location often turned out to be important, suggesting that some more complex processes involving observer effects and instantaneous fish displacement might be involved. This corroborates with the results of visual census studies in which observer biases were less important than sampling variability (Edgar et al., 2004; Williams et al., 2006). For the observed structure of the fish assemblages and the diversity estimates, i.e. species density and Shannon diversity, these interaction effects were considerably smaller than the effects attributed to the factors Location and Transect. However, for the univariate species count models, 18 out of 36 species had considerably high inter-observer variabilities compared to the effects attributed to the factors Location and Transect. Observers covered the transects sequentially, meaning that each observer first finished six observations of one transect before moving to another transect. Therefore, one would expect that observations closer in time would be more similar, causing significant interaction effects of the factors Transect and Observer. Although mostly not significant, there were indeed some clear patterns in temporal auto-correlation for some species, with similar amounts of species being significantly deterred (5) or attracted (7) by observers. In addition, differences in detectability due to intrinsic factors of fish species such as crypsis and color or extrinsic factors such as

observer behavior may have caused an additional bias (Boulinier et al., 1998; MacNeil et al., 2008b; Willis, 2001). For example, the Marbled goby *(Gobio manchada)*, a shy and cryptic species that hides in crevices, was scared away by observers, while the Panamic sergeant major *(Abudefduf troschelii)* and Mexican hogfish (*Bodianus diplotaenia*), two pelagic curious species, appeared attracted to observers as they were typically recorded more often during the first and last observations, respectively. It was unlikely that individuals of scared-away fish species would be recorded by the second and third observer, while curious fish would be more likely to be observed by all observers. Indeed, models for fish that were scared away typically showed higher levels of inter-observer variability than models for fish that seemed to be attracted to the observer.

Besides the effect of observers on fish behavior, there are also effects of fish behavior on observer perception. Schooling behaviour may cause higher inter-observer variability (Kulbicki, 1998; McClanahan et al., 2007). As a single observer records a school of fish that moves out of view during the subsequent observations of other observers surveying that specific transect or location, the effect on inter-observer variability would be much larger than when a single individual would move out of view. This was most likely the case for the White salema (*Xenichthys agassizii*) and Galapagos grunt (*Orthopristis forbesi*) which are known to spatially cluster and move around in group. Similarly, the displacement of individual fish in and out of view will have a stronger effect on the inter-observer variability of the counts of rare

species (such as the Jewel moray (*Muraena lentiginosa*)) compared to that of more common species, as instantaneous fish displacement will affect fish density more severely, potentially even causing species to be detected during only some of the observations of a specific transect or location (Boulinier et al., 1998).

Finally, the behavior of an observer might also affect his/her perception. Individual observers covering a transect multiple times may gain knowledge regarding the location of species that were hiding and may unconsciously give these locations more attention. For example, on the level of a single observer, once the sedentary Bravo clinid (*Gobioclinus dendriticus*) was detected, it was much more likely that it would also be detected during successive observations. Therefore, the interaction effects are likely the result of a combination of temporal auto-correlation between observations, instantaneous fish displacement and biases related to traits of both fish and observer.

Overall, for the multivariate structure of the fish assemblages, the factor Observer and its interaction with spatial factors turned out to be much less important than the factors Location and Transect. This result is in strong contrast with the results of traditional visual census studies which are often faced with high levels of inter-observer variability (Bernard et al., 2013; Edgar et al., 2004; Dickens et al., 2011; Tessier et al., 2013). Although, the "unwanted" sampling variability associated with instantaneous fish displacement, observer effects and random counting/detection errors could be relatively large for short transects (1.42 times the variability explained by

transects and locations), longer transects allowed for a significant improvement (0.69 times the variability explained by transects and locations). Although not assessed in this study, detectabilty of species has been shown to be positively related to density (Dorazio and Royle, 2005). Hence, the choice of the transformation and/or dissimilarity measure, which differ in the weights given to rare and common species, might also affect the importance of observer effects and instantaneous fish displacement. In addition, if researchers are interested in the distributions of individual species, species-specific observer effects and instantaneous fish displacement might still be very important as the proportion of sampling variability of most species (i.e. 28 out of 36 species for transect lengths of 50 meters) exceeded the proportion of variability explained by the sampling units and sites.

The overall low observer bias suggests that one observer is sufficient and that observations of different observers under similar conditions are interchangeable, given these observers received a similar training. Training can limit counting/detection errors and observer effects to some extent, but an important part of the sampling variability related to the observer effects, counting/detection errors, and instantaneous fish displacement will remain. Although this “uncontrollable” yet important part of the sampling variability cannot be reduced, it can be accounted for when sufficient repetitions of sufficiently long transects are available (see section 4.2).

4.2. Design error and sampling variability

The definition of a design error depends on the hypothesis and on the scale of a study. For example, if one is interested in the association of a species or fish assemblage with different classified habitat patches, the size of the repeated sampling unit is likely to affect the sampling variability. If instead, one is interested in the association of a species or fish assemblage with a specific area, sampling units that are too small may introduce a design error because of their inability to account for the range of different habitat types present in the study area.

Although habitat patches were not explicitly classified in this study, each transect (or each subset of a transect) could be thought of as a unique series of habitat patches that did not alter during sampling. To assess whether species or fish assemblages are effectively associated with a specific transect (or subset of a transect), some repeated observations are required. Precision quantifies the concordance of multiple observations and was determined for species density, Shannon diversity and counts of individual species of each transect. The precision of most parameters increased with the number of repeats and the transect length. Hence, longer transect lengths were characterized by lower sampling variabilities. In addition, the lower CAP cross-validation classification errors (Table 4) and the lower dissimilarity values of the longer transects (Fig. 3) suggested that also for a multivariate response (e.g. fish assemblage structure), sampling variability decreased with increasing sampling length.

In addition to associations with habitats, researchers are often interested in associations of fish species or assemblages with specific areas or locations. Instead of the assessment of small-scale relationships between fish and their environment, one then wants to obtain a more general idea of how fish are affected by their surrounding environment. Such an approach makes sense, given the high mobility of different fish species and the often strong spatiotemporal dynamics of environmental conditions in coastal areas (Bruneel et al., 2018). Therefore, rather than assessing the sampling variability within transects, the variability between transects and between locations should be assessed. The linear mixed models of species density and Shannon diversity fitted the data better with increasing transect length, which is likely a consequence of the smaller chance of discovering additional species with further sampling effort in a specific location or transect, as can be seen in the species accumulation curves of Figs. E.1, E.2 and E.3. Similarly, the PERMANOVA models fitted the data better (Table 3) and CAP cross-validations had lower classification errors for the different locations (Table 4) with increasing transect length. If more different micro-habitats are monitored, the observed fish assemblages within a location will likely be more representative for that location (Fig. H.2). The improved goodness-of-fit of the PERMANOVA models with increasing transect length (R^2_{10m}=0.761; R^2_{50m}=0.880), could mainly be attributed to the factors Location ($R^2_{Location;10m}$=0.145; $R^2_{Location;50m}$=0.212) and to a lesser extent to Transect ($R^2_{Transect;10m}$=0.117; $R^2_{Transect;50m}$=0.142), suggesting that, in addition to the reduced sampling variability within tran-

sects, the representativeness of the transects for a specific location improved. The Observer-related factors of the PERMANOVA models remained limited, suggesting that, unlike the sampling variability and design error, the inter-observer variability did not change much from short to long transects (Table 3). In addition, the design error associated with short transects did not appear to impede multivariate analyses of the assemblage structure for the transect lengths considered in this study: Even the lowest transect length of 10 meters provided interpretable data for analyses of the assemblage structure as uninformative dissimilarity values were almost non-existent, indicating that there was no need to pool samples (Fig. 3).

Not only the procedures to collect video material, but also the procedures to analyze them should be evaluated. The PERMANOVA model using log-transformed MaxCount data from 50-meter transects with six repeats performed best, while the PERMANOVA models using presence-absence or transformed MinCount data of lower transect lengths performed worst (Fig. H.1). MaxCount models were significantly better in explaining the variability than the MinCount models, probably because the latter may not scale linearly with true abundance (Schobernd et al., 2014). MinCount is often used instead of MaxCount in video monitoring studies to avoid double counts (Schobernd et al., 2014). However, since we used video transects rather than stationary devices, the risk of double counting was considerably less. Although neglecting the abundance of a species scored worst in terms of goodness-of-fit, the difference between MinCount and presence-absence

data was rather small. Therefore, in this specific case, if one wanted to reduce the time required for video analysis, the presence-absence instead of the abundance data could have been used, without much information being lost.

Transect length remained the most important parameter affecting the performance of the PERMANOVA models, but longer transects also increased the time required for both the data collection and processing. The square root of the residual mean square of the PERMANOVA analysis decreased with increasing number of repeats and with increasing transect length. This proxy for multivariate precision was more affected by number of repeats than by transect length, suggesting that more repeats are preferred over longer transects to increase the precision. However, more repeats do not alter the representativeness of the sampling unit for the study area. Therefore, the choice between more repeats or longer transects should be guided by the required level of precision and representativeness of the sampling unit for the study area.

The precision of the MaxCount of most encountered species, with the exception of the Galapagos Sea Bream (*Archosargus pourtalesi*) and the Black Striped Salema (*Xenocys jessiae*), was also affected more strongly by the number of repeats than by transect length. Although there was a clear increase in the precision of species density and Shannon diversity with total swim distance, it did not matter much whether longer transect lengths or more repeats were applied. For the assessment of species density, 10-meter transects were slightly more efficient, while for the Shannon diversity, 20 to

30-meter transects were slightly more efficient. In addition to the number of repeats and the transect length, the visibility also alters the precision (Figueroa-Pico et al., 2020; Bicknell et al., 2016; MacNeil et al., 2008a). A higher turbidity resulted in a lower visibility which caused clearly lower precision levels of the species density and Shannon diversity. Observers had to swim longer transect lengths or perform more repeats in turbid conditions compared to clear water conditions. Overall, if researchers want to improve precision because of low visibility or pronounced spatial and temporal dynamics, more repeats should be preferred over longer transects. The improved ability to account for the existing sampling variability when including more repeats is stronger (per unit of sampling effort) than the decrease in sampling variability when using longer transects.

It should be noted that, besides the objectives of the study, time constraints and the nature of the parameter of interest, the most optimal distribution of the sampling effort among different methodological aspects may also be affected by more local logistic constraints. For example, in order to chose between more repeats and longer transects, researchers need to balance the expected degree of representativeness, precision and disturbance tied up with the sampling units. On the one hand, it may be more difficult to maintain homogeneous areas with respect to the observed fish assemblages when transects are longer (Anderson and Santana-Garcon, 2015), but on the other hand, more repeats of the same transect might cause more disturbances and may be less informative when the objective is to obtain representative ob-

servations of the local fish assemblages. More and longer transects would in that case be more suitable.

5. Conclusion

Deciding on the distribution of the sampling effort among different methodological aspects is an important step for any study. In this study we described how researchers should take into account a broad range of potentially conflicting theoretical and practical aspects when setting up an experimental design for video transects. Sampling variability, methodological parameters, environmental conditions, and ecological knowledge, e.g. species-specific traits affecting detectability, were found important for video transects and should be accounted for to properly assess the ecological state and the effect of management practices on the environment.

Acknowledgements

All methods were carried out in accordance with the relevant guidelines and regulations of the Galapagos National Park Directorate under research permit PC-02-19. All experimental protocols were reviewed and approved by the Galapagos National Park Directorate Applied Research Department, which assesses animal care in research activities. N. De Troyer, L. Dominguez, M. Forio, M. Guerra, R. Narea, J. Ponton, A. Rosado, R. Sor, J. Torrico, T. Truong Trinh Tu, C. Van der heyden, R. Vandeputte aided in the

data collection. This research has benefited from a statistical consult with Ghent University FIRE (Fostering Innovative Research based on Evidence).

Authors' contributions

SB+RB+PG: Conceptualization, SB: Data curation, Visualization, Writing - original draft, Formal analysis, Software, RB+PG: Funding acquisition, Supervision, Project administration, SB+LH+HR+AS+PG: Investigation, SB+RB+PG: Methodology, RB+PG: Resources, All authors: Writing - review and editing

Funding

The research was partly funded by VLIR-UOS Biodiversity Network Ecuador, Special Research Fund of UGent and VLIR-UOS Global Minds. Travelling scholarships for staff and students were provided by FWO, CWO (UGent), VLIR-UOS (Global Minds) and VLIR-UOS Biodiversity Network Ecuador.

Data accessibility

The R code, subset of the data and documentation will be made available via Mendeley Data after acceptance of the manuscript.

6. References

Anderson, M., Willis, T., 2003. Canonical Analysis of Principal Coordinates: a Useful Method of Constrained Ordination for Ecology. Ecology 84 (2), 511–525.

Anderson, M. J., Gorley, R. N., Clarke, K. R., 2008. PERMANOVA+ for PRIMER: Guide to Software and Statistical Methods.

Anderson, M. J., Santana-Garcon, J., 2015. Measures of precision for dissimilarity-based multivariate analysis of ecological communities. Ecology Letters 18 (1), 66–73.

Andradi-Brown, D. A., Macaya-Solis, C., Exton, D. A., Gress, E., Wright, G., Rogers, A. D., 2016. Assessing caribbean shallow and mesophotic reef fish communities using Baited-Remote Underwater Video (BRUV) and diver-operated video (DOV) survey techniques. PLoS ONE 11 (12), 1–23.

Andrew, N., Mapstone, B., 1987. Sampling and the description of spatial pattern in marine ecology. Oceanogr. Mar. Biol. Annu. Rev. 25, 39–90.

Bernard, A. T. F., Götz, A., Kerwath, S. E., Wilke, C. G., 2013. Observer bias and detection probability in underwater visual census of fish assemblages measured with independent double-observers. Journal of Experimental Marine Biology and Ecology 443, 75–84.

Bicknell, A. W., Godley, B. J., Sheehan, E. V., Votier, S. C., Witt, M. J.,

2016. Camera technology for monitoring marine biodiversity and human impact. Frontiers in Ecology and the Environment 14 (8), 424–432.

Bijoux, J. P., Dagorn, L., Gaertner, J. C., Cowley, P. D., Robinson, J., 2013. The influence of natural cycles on coral reef fish movement: Implications for underwater visual census (UVC) surveys. Coral Reefs 32 (4), 1135–1140.

Blasco-Moreno, A., Pérez-Casany, M., Puig, P., Morante, M., Castells, E., 2019. What does a zero mean? Understanding false, random and structural zeros in ecology. Methods in Ecology and Evolution 10, 949–959.

Boom, B. J., Huang, P. X., Beyan, C., Spampinato, C., Palazzo, S., He, J., Beauxis-Aussalet, E., Lin, S. I., Chou, H. M., Nadarajan, G., 2012. Long-Term Underwater Camera Surveillance for Monitoring and Analysis of Fish Populations, 2–5.

Boulinier, T., Nichols, J. D., Sauer, J. R., Hines, J. E., Pollock, K. H., 1998. Estimating species richness: The importance of heterogeneity in species detectability. Ecology 79 (3), 1018–1028.

Brooks, M. E. J. K. K., van Benthem, K., Magnusson, A., Berg, C. W., Nielsen, A., Skaug, H. J., Maechler, M., Bolker, B. M., 2017. glmmTMB balances speed and flexibility among packages for zero-inflated generalized linear mixed modeling. The R Journal 9 (2), 378–400.

Bruneel, S., Gobeyn, S., Verhelst, P., Reubens, J., Moens, T., Goethals, P., 2018. Implications of movement for species distribution models - Rethinking environmental data tools. Science of the Total Environment 628-629, 893–905.

Caldwell, Z. R., Zgliczynski, B. J., Williams, G. J., Sandin, S. A., 2016. Reef Fish Survey Techniques: Assessing the Potential for Standardizing Methodologies. PLoS ONE 11 (4), 1–14.

Cappo, M., Harvey, E., Malcolm, H., Speare, P., 2003. Potential of video techniques to monitor diversity , abundance and size of fish in studies of Marine Protected Areas. In: Beumer, J., Grant, A., Smith, D. (Eds.), Aquatic Protected Areas – What Works Best and How Do We Know? pp. 455–464.

Cappo, M., Harvey, E. S., Shortis, M., 2006. Counting and measuring fish with baited video techniques - an overview. In: Australian Society for fish biology. pp. 101–114.

Cappo, M., Stowar, M., Syms, C., Johansson, C., Cooper, T., 2011. Fish-habitat associations in the region offshore from James price point - a rapid assessment using baited remote underwater video stations (BRUVS). Journal of the Royal Society of Western Australia 94 (2), 303–321.

Chapman, M. R., Kramer, D. L., 2000. Movements of fishes within and

among fringing coral reefs in Barbados. Environmental Biology of Fishes 57 (1), 11–24.

Clarke, K. R., Gorley, R. N., 2006. PRIMER v6: Manual/Tutorial. PRIMER-E, Plymouth.

Clarke, K. R., Somerfield, P. J., Chapman, M. G., 2006. On resemblance measures for ecological studies , including taxonomic dissimilarities and a zero-adjusted Bray – Curtis coefficient for denuded assemblages. Journal of Experimental Marine Biology and Ecology 330 (1), 55–80.

Denny, C., Willis, T. J., 2003. Effects of Poor Knights Islands Marine Reserve on demersal fish populations. DOC Science Internal Series 142.

Dickens, L. C., Goatley, C. H., Tanner, J. K., Bellwood, D. R., 2011. Quantifying relative diver effects in underwater visual censuses. PLoS ONE 6 (4).

Dorazio, R. M., Royle, J. A., 2005. Estimating size and composition of biological communities by modeling the occurrence of species. Journal of the American Statistical Association 100 (470), 389–398.

Dornelas, M., Connolly, S. R., Hughes, T. P., 2006. Coral reef diversity refutes the neutral theory of biodiversity. Nature.

Edgar, G. J., Barrett, N. S., Morton, A. J., 2004. Biases associated with the use of underwater visual census techniques to quantify the density and size-structure of fish populations. Journal of Experimental Marine Biology and Ecology 308 (2), 269–290.

Figueroa-Pico, J., Carpio, A. J., Tortosa, F. S., 2020. Turbidity: A key factor in the estimation of fish species richness and abundance in the rocky reefs of Ecuador. Ecological Indicators 111.

Ford, A. K., Eich, A., McAndrews, R. S., Mangubhai, S., Nugues, M. M., Bejarano, S., Moore, B. R., Rico, C., Wild, C., Ferse, S. C., 2018. Evaluation of coral reef management effectiveness using conventional versus resilience-based metrics. Ecological Indicators 85, 308–317.

Gladstone, W., Lindfield, S., Coleman, M., Kelaher, B., 2012. Optimisation of baited remote underwater video sampling designs for estuarine fish assemblages. Journal of Experimental Marine Biology and Ecology 429, 28–35.

Goetze, J. S., Bond, T., McLean, D. L., Saunders, B. J., Langlois, T. J., Lindfield, S., Fullwood, L. A., Driessen, D., Shedrawi, G., Harvey, E. S., 2019. A field and video analysis guide for diver operated stereo-video. Methods in Ecology and Evolution 10 (7), 1083–1090.

González-Sansón, G., Aguilar, C., 2010. Reef fish diversity components as indicators of cumulative effects in a highly impacted fringe reef. Ecological Indicators 10 (3), 766–772.

Guillemot, N., Chabanet, P., Kulbicki, M., Vigliola, L., Léopold, M., Jollit, I., Le Pape, O., 2014. Effects of fishing on fish assemblages in a coral reef

ecosystem: From functional response to potential indicators. Ecological Indicators 43, 227–235.

Halpern, B. S., Selkoe, K. A., Micheli, F., Kappel, C. V., 2007. Evaluating and ranking the vulnerability of global marine ecosystems to anthropogenic threats. Conservation Biology 21 (5), 1301–1315.

Harvey, E., Fletcher, D., Shortis, M., 2001. Improving the statistical power of length estimates of reef fish: A comparison of estimates determined visually by divers with estimates produced by a stereo-video system. Fishery Bulletin.

Imoto, T., 2014. A generalized Conway-Maxwell-Poisson distribution which includes the negative binomial distribution. Applied Mathematics and Computation 247, 824–834.

Irigoyen, A. J., Galván, D. E., Venerus, L. A., Parma, A. M., 2013. Variability in Abundance of Temperate Reef Fishes Estimated by Visual Census. PLoS ONE 8 (4), 1–12.

Jones, T., Davidson, R. J., Gardner, J. P., Bell, J. J., 2015. Evaluation and optimisation of underwater visual census monitoring for quantifying change in rocky-reef fish abundance. Biological Conservation 186, 326–336.

Katsanevakis, S., Weber, A., Pipitone, C., Leopold, M., Cronin, M., Scheidat, M., Doyle, T. K., Buhl-Mortensen, L., Buhl-Mortensen, P., D'Anna, G., de Boois, I., Dalpadado, P., Damalas, D., Fiorentino, F., Garofalo, G.,

Giacalone, V. M., Hawley, K. L., Issaris, Y., Jansen, J., Knight, C. M., Knittweis, L., Kröncke, I., Mirto, S., Muxika, I., Reiss, H., Skjoldal, H. R., Vöge, S., 2012. Monitoring marine populations and communities: Methods dealing with imperfect detectability. Aquatic Biology 16 (1), 31–52.

Kulbicki, M., 1998. How the acquired behaviour of commercial reef fishes may influence the results obtained from visual censuses. Journal of Experimental Marine Biology and Ecology 222 (1-2), 11–30.

Labrosse, P., Kulbicki, M., Ferraris, J., 2002. Underwater fish visual census surveys. Tech. rep.

Langlois, T. J., Harvey, E. S., Fitzpatrick, B., Meeuwig, J. J., Shedrawi, G., Watson, D. L., 2010. Cost-efficient sampling of fish assemblages: Comparison of baited video stations and diver video transects. Aquatic Biology 9 (2), 155–168.

Legendre, P., Legendre, L., 2012. Numerical ecology, 3rd Edition. Elsevier.

Lindfield, S. J., Harvey, E. S., Mcilwain, J. L., Halford, A. R., 2014. Silent fish surveys: Bubble-free diving highlights inaccuracies associated with SCUBA-based surveys in heavily fished areas. Methods in Ecology and Evolution 5 (10), 1061–1069.

Lynch, H. J., Thorson, J. T., Shelton, A. O., 2014. Dealing with under- and over-dispersed count data in life history, spatial, and community ecology. Ecology 95 (11), 3173–3180.

MacNeil, M. A., Graham, N. A., Conroy, M. J., Fonnesbeck, C. J., Polunin, N. V., Rushton, S. P., Chabanet, P., McClanahan, T. R., 2008a. Detection heterogeneity in underwater visual-census data. Journal of Fish Biology 73 (7), 1748–1763.

MacNeil, M. A., Tyler, E. H., Fonnesbeck, C. J., Rushton, S. P., Polunin, N. V., Conroy, M. J., 2008b. Accounting for detectability in reef-fish biodiversity estimates. Marine Ecology Progress Series 367, 249–260.

Mallet, D., Pelletier, D., 2014. Underwater video techniques for observing coastal marine biodiversity: A review of sixty years of publications (1952-2012). Fisheries Research 154, 44–62.

Manly, B. F. J., 1992. Bootstrapping for determining sample sizes in biological studies. Journal of Experimental Marine Biology and Ecology 158 (2), 189–196.

McClanahan, T. R., Graham, N. A., Maina, J., Chabanet, P., Bruggemann, J. H., Polunin, N. V., 2007. Influence of instantaneous variation on estimates of coral reef fish populations and communities. Marine Ecology Progress Series 340, 221–234.

Murphy, H. M., Jenkins, G. P., 2010. Observational methods used in marine spatial monitoring of fishes and associated habitats: A review. Marine and Freshwater Research 61 (2), 236–252.

Nakagawa, S., Schielzeth, H., 2017. Coefficient of determination R 2 and intra-class correlation coefficient ICC from generalized linear mixed-effects models. Journal of the Royal Society Interface 14.

Pais, M. P., Cabral, H. N., 2017. Fish behaviour effects on the accuracy and precision of underwater visual census surveys. A virtual ecologist approach using an individual-based model. Ecological Modelling 346, 58–69.

Pelletier, D., Leleu, K., Mallet, D., Mou-Tham, G., Hervé, G., Boureau, M., Guilpart, N., 2012. Remote high-definition rotating video enables fast spatial survey of marine underwater macrofauna and habitats. PLoS ONE 7 (2), 1–13.

Pelletier, D., Leleu, K., Mou-Tham, G., Guillemot, N., Chabanet, P., 2011. Comparison of visual census and high definition video transects for monitoring coral reef fish assemblages. Fisheries Research 107 (1-3), 84–93.

Ruttenberg, B. I., Haupt, A. J., Chiriboga, A. I., Warner, R. R., 2005. Patterns, causes and consequences of regional variation in the ecology and life history of a reef fish. Oecologia 145 (3), 394–403.

Samoilys, M. A., Carlos, G., 2000. Determining methods of underwater visual census for estimating the abundance of coral reef fishes. Environmental Biology of Fishes 57, 289–304.

Schmid, K., Reis-Filho, J. A., Harvey, E., Giarrizzo, T., 2017. Baited remote underwater video as a promising nondestructive tool to assess fish

assemblages in clearwater Amazonian rivers: testing the effect of bait and habitat type. Hydrobiologia 784 (1), 93–109.

Schobernd, Z. H., Bacheler, N. M., Conn, P. B., Trenkel, V., 2014. Examining the utility of alternative video monitoring metrics for indexing reef fish abundance. Canadian Journal of Fisheries and Aquatic Sciences 71 (3), 464–471.

Schramm, K. D., Harvey, E. S., Goetze, J. S., Travers, M. J., Warnock, B., Saunders, B. J., 2020. A comparison of stereo-BRUV, diver operated and remote stereo-video transects for assessing reef fish assemblages. Journal of Experimental Marine Biology and Ecology 524.

Sward, D., Monk, J., Barrett, N., 2019. A Systematic Review of Remotely Operated Vehicle Surveys for Visually Assessing Fish Assemblages. Frontiers in Marine Science 6, 134.

Tessier, A., Pastor, J., Francour, P., Saragoni, G., Crec'hriou, R., Lenfant, P., 2013. Video transects as a complement to underwater visual census to study reserve effect on fish assemblages. Aquatic Biology 18 (3), 229–241.

Thompson, A. A., Mapstone, B. D., 1997. Observer effects and training in underwater visual surveys of reef fishes. Marine Ecology Progress Series 154, 53–63.

Wartenberg, R., Booth, A. J., 2015. Video transects are the most appropriate underwater visual census method for surveying high-latitude coral

reef fishes in the southwestern Indian Ocean. Marine Biodiversity 45 (4), 633–646.

Watson, D. L., Harvey, E. S., 2007. Behaviour of temperate and sub-tropical reef fishes towards a stationary SCUBA diver. Marine and Freshwater Behaviour and Physiology.

Watson, D. L., Harvey, E. S., Anderson, M. J., Kendrick, G. A., 2005. A comparison of temperate reef fish assemblages recorded by three underwater stereo-video techniques. Marine Biology 148 (2), 415–425.

Watson, D. L., Harvey, E. S., Fitzpatrick, B. M., Langlois, T. J., Shedrawi, G., 2010. Assessing reef fish assemblage structure: How do different stereo-video techniques compare? Marine Biology 157 (6), 1237–1250.

Williams, I. D., Walsh, W. J., Tissot, B. N., Hallacher, L. E., 2006. Impact of observers' experience level on counts of fishes in underwater visual surveys. Marine Ecology Progress Series 310, 185–191.

Willis, T. J., 2001. Visual census methods underestimate density and diversity of cryptic reef fishes. Journal of Fish Biology 59 (5), 1408–1411.

Willis, T. J., Millar, R. B., Babcock, R. C., 2000. Detection of spatial variability in relative density of fishes: Comparison of visual census, angling, and baited underwater video. Marine Ecology Progress Series 198, 249–260.

Wilson, S. K., Graham, N. A., Holmes, T. H., MacNeil, M. A., Ryan, N. M.,

2018. Visual versus video methods for estimating reef fish biomass. Ecological Indicators 85, 146–152.

Zintzen, V., Anderson, M. J., Roberts, C. D., Harvey, E. S., Stewart, A. L., Struthers, C. D., 2012. Diversity and Composition of Demersal Fishes along a Depth Gradient Assessed by Baited Remote Underwater Stereo-Video. PLoS ONE 7 (10).

Appendix A. Additional information

Appendix A.1. Instantaneous fish displacement

As fish displacement is not necessarily random, even at very short time scales, we prefer to use the term instantaneous fish displacement, instead of random fish displacement (Irigoyen et al., 2013; MacNeil et al., 2008b), throughout the article to include all fish displacement that would take place under natural conditions at very short time scales. While the instantaneous displacement causes actual variation in the local (i.e. within sampling unit) abundance, the random counting/detection errors introduce false variation in local abundance.

Appendix A.2. Applications of video monitoring

Video material has been used for different purposes at different times after the video material was collected. It has been used to characterize physical habitats (Cappo et al., 2003), to obtain biomass estimates (Harvey et al., 2001), to study fish behavior (Watson and Harvey, 2007), and to train algorithms to pick out fish and identify them automatically (Boom et al., 2012). In addition, because the collecting and analyzing of the video material can be done by two different persons, people trained in underwater video collection do not necessarily have to be trained in identifying and counting fish.

Appendix B. Video analysis

Fish were counted in such a way that both the MinCount and MaxCount of each observation could be determined. For example, if two individuals of the same species were recorded in $frame_1$ and a few moments later a third individual of the same species entered the field of view (FOV) of $frame_2$, while the other two individuals were still present in the FOV, than only the recording of $frame_2$ would be included. However, if the two individuals encountered in $frame_1$ moved away before the individual of $frame_2$ moved within the FOV, then both recordings were included separately as the third individual may actually have been one of the first two individuals.

Appendix C. Independence of observations

Species	lag 0	lag 1	lag 2	lag 3	lag 4	lag 5	lag 6	lag 7	lag 8
Amarillo snapper (*Lutjanus argentiventris*)	1	-0.03	-0.05	-0.02	-0.11	0	-0.03	-0.1	-0.01
Balloon fish (*Diodon holocanthus*)	1	-0.06	-0.07	-0.07	-0.07	-0.08	-0.08	-0.08	-0.03
Banded wrasse (*Halichoeres notospilus*)	1	0.2	0.16	0.03	-0.03	-0.07	-0.13	-0.15	-0.07
Black striped salema (*Xenocys jessiae*)	1	-0.01	0.03	0.02	0.03	-0.01	-0.04	-0.09	-0.05
Blacktip cardinalfish (*Apogon atradorsatus*)	1	0.06	0.14	-0.03	0.04	-0.08	-0.09	-0.13	-0.11
Blue and gold snapper (*Lutjanus viridis*)	1	0.06	0.07	-0.09	-0.11	-0.09	-0.1	-0.03	-0.04
Blue chin parrotfish (*Scarus ghobban*)	1	0.02	0.01	0.04	0.02	-0.07	-0.07	-0.03	-0.04
Bravo clinid (*Gobioclinus dendriticus*)	1	0.16	0.11	0.01	0.02	-0.03	-0.08	-0.1	-0.14
Bullseye puffer (*Sphoeroides annulatus*)	1	0.09	-0.05	-0.07	0.01	-0.04	-0.1	-0.11	-0.06
Chameleon wrasse (*Halichoeres dispilus*)	1	0.29	0.18	0	0	-0.03	-0.09	-0.09	-0.09
Cortez rainbow wrasse (*Thalassoma lucasanum*)	1	0.13	0.17	0	-0.01	-0.07	-0.13	-0.1	-0.06
Eagle ray (*Aetobatus narinari*)	1	-0.06	-0.07	-0.07	-0.07	-0.02	-0.02	-0.02	-0.03
Flag cabrilla (*Epinephelus labriformis*)	1	0.04	0.01	-0.04	-0.07	-0.06	-0.07	-0.03	-0.04
Galapagos bullhead shark (*Heterodontus quoyi*)	1	0	-0.01	-0.01	-0.01	-0.02	-0.02	-0.02	-0.03
Galapagos grunt (*Orthopristis forbesi*)	1	0.21	0.03	0.01	0.09	-0.01	-0.17	-0.11	-0.07
Galapagos ringtail damselfish (*Stegastes beebei*)	1	0.26	0.22	0.04	0.03	-0.11	-0.12	-0.11	-0.15
Galapagos Seabream (*Archosargus pourtalesi*)	1	0.27	-0.05	0	0.09	-0.06	-0.18	-0.04	-0.08
Galapagos shark (*Carcharhinus galapagensis*)	1	-0.06	-0.05	-0.05	-0.03	-0.02	-0.02	-0.02	-0.03
Galapagos sheepshead wrasse (*Semicossyphus darwini*)	1	-0.06	-0.07	-0.07	-0.07	-0.08	-0.02	-0.02	-0.03
Galapagos triplefin blenny (*Lepidonectes corallicola*)	1	0.16	-0.03	-0.01	-0.04	-0.04	-0.08	-0.07	-0.03
Giant hawkfish (*Cirrhitus rivulatus*)	1	-0.02	0.13	-0.03	-0.07	-0.07	-0.14	-0.05	-0.1
Harlequin wrasse (*Bodianus eclancheri*)	1	0.73*	0.47*	0.2	-0.06	-0.08	-0.1	-0.11	-0.13
Jewel moray (*Muraena lentiginosa*)	1	-0.06	-0.06	-0.06	0.13	-0.05	-0.06	-0.05	-0.05
King angelfish (*Holacanthus passer*)	1	0.06	-0.02	-0.02	-0.01	-0.05	-0.07	-0.06	-0.06
Loosetooth parrotfish (*Nicholsina denticulata*)	1	-0.06	-0.07	-0.07	-0.07	-0.02	-0.02	-0.02	-0.03
Marbled goby (*Gobio manchada*)	1	0.17	0.15	0.04	0.02	-0.06	-0.11	-0.12	-0.12
Marbled ray (*Taeniurops meyeni*)	1	-0.06	-0.07	-0.07	-0.01	-0.02	-0.02	-0.02	-0.03
Mexican hogfish (*Bodianus diplotaenia*)	1	0.11	0.07	0.04	0.05	-0.07	-0.04	-0.07	-0.08
Mojarra grunt (*Haemulon scudderii*)	1	0	0.03	0	0.01	-0.05	-0.04	-0.04	-0.05
Mullet snapper (*Lutjanus aratus*)	1	0.42	-0.08	-0.02	-0.03	-0.03	-0.04	-0.05	-0.06
Pacific dog snapper (*Lutjanus novemfasciatus*)	1	0.46*	0.2	0.29	0.32	0.14	-0.13	-0.15	-0.17
Pacific spotfin mojarra (*Eucinostomus dowii*)	1	0.14	0.09	0.07	0.03	0.01	0.02	-0.11	-0.04
Panamic fanged blenny (*Ophioblennius steindachneri*)	1	0	0	-0.07	0	-0.09	-0.07	-0.04	-0.03
Panamic sergeant major (*Abudefduf troschelii*)	1	0.37	0.19	0.07	0.04	-0.06	-0.1	-0.14	-0.08
Razor surgeonfish (*Prionurus laticlavius*)	1	0.06	0.16	-0.09	-0.1	-0.07	-0.15	-0.06	-0.05
Reef cornetfish (*Fistularia commersonii*)	1	0.29	0.06	-0.04	-0.03	-0.02	-0.09	-0.08	-0.08
Sabertooth blenny (*Plagiotremus azaleus*)	1	0.12	0.08	0.01	-0.04	-0.11	-0.12	-0.13	-0.07
Spinster wrasse (*Halichoeres nicholsi*)	1	0.11	0.12	0.02	-0.03	-0.06	-0.06	-0.04	-0.05
Spotted cabrilla (*Epinephelus analogus*)	1	0	-0.01	-0.01	-0.01	-0.02	-0.02	-0.02	-0.03
Stone scorpionfish (*Scorpaena mystes*)	1	0.27	0.08	0.04	-0.02	-0.08	-0.07	-0.08	-0.08
Striped mullet (*Mugil cephalus*)	1	-0.06	-0.01	-0.01	-0.01	-0.02	-0.02	-0.02	-0.03
Three banded butterfly fish (*Chaetodon humeralis*)	1	0.07	0.05	0.04	-0.04	-0.13	0.04	-0.04	-0.07
Throat-spotted blenny (*Malacoctenus tetranemus*)	1	0.29	-0.07	-0.06	-0.06	-0.07	-0.08	-0.08	-0.09
Tiger snake eel (*Myrichthys maculosus*)	1	0.04	-0.07	-0.08	-0.01	-0.04	-0.05	-0.13	-0.09
Triplefin blenny (*Lepidonectes corallicola*)	1	-0.06	-0.01	-0.01	-0.01	-0.02	-0.02	-0.02	-0.03
White mullet (*Mugil curema*)	1	-0.13	0.42	-0.15	-0.09	-0.1	-0.04	-0.05	-0.06
White salema (*Xenichthys agassizii*)	1	0.13	0.07	0.1	0.03	-0.1	-0.08	-0.1	-0.09
Wounded wrasse (*Halichoeres chierchiae*)	1	0.17	0.15	0.1	0.04	-0.03	-0.12	-0.12	-0.1
Yellowtail damselfish (*Microspathodon bairdii*)	1	0.14	0.18	-0.02	0.02	-0.08	-0.08	-0.16	-0.09

Table C.1: Averaged auto-correlation function (ACF) for observation lags from 0 to 8. Per transect 18 observations were made by three different observers subsequently. The considered response variables were the fourth-root transformed counts of every species. Auto-correlations were determined per transect and averaged afterwards. Per species, only transects were considered of which at least one observation had at least one observed individual. significant auto-correlations were indicated (p<0.05) with *.

Species	Variable	18 Observations~					6 Observations				
		Estimate	SE	z-value	p-value	n	Estimate	SE	z-value	p-value	n
Amarillo snapper	Intercept	-2.48	0.61	-4.05	0.000052	7	-1.13	0.56	-2.01	0.044399	14
Lutjanus argentiventris	Order	0.09	0.05	1.98	0.047997		0.06	0.14	0.42	0.671628	
Balloonfish	Intercept	-3.45	2.45	-1.41	0.159794	1	-4.93	4.61	-1.07	0.284486	1
Diodon holocanthus	Order	0.06	0.20	0.29	0.768144		0.79	0.94	0.85	0.397174	
Banded wrasse	Intercept	-0.87	0.31	-2.76	0.005776	21	0.12	0.38	0.32	0.747055	29
Halichoeres notospilus	Order	-0.03	0.02	-1.46	0.144351		-0.05	0.09	-0.57	0.565406	
Black striped salema	Intercept	-0.57	0.68	-0.84	0.401543	6	-0.12	0.84	-0.14	0.891117	10
Xenocys jessiae	Order	0.02	0.04	0.52	0.603868		0.14	0.15	0.92	0.356786	
Blacktip cardinalfish	Intercept	0.32	0.70	0.46	0.648619	12	0.49	0.66	0.74	0.457521	20
Apogon atradorsatus	Order	0.02	0.03	0.69	0.490417		0.09	0.11	0.82	0.409622	
Blue and gold snapper	Intercept	-2.23	0.72	-3.11	0.001862	7	0.47	0.71	0.67	0.504090	10
Lutjanus viridis	Order	0.04	0.05	0.79	0.429302		-0.41	0.18	-2.26	0.023927	
Blue chin parrotfish	Intercept	-1.67	0.63	-2.66	0.007844	6	-0.47	0.65	-0.72	0.470633	9
Scarus ghobban	Order	0.00	0.05	-0.08	0.939047		-0.04	0.17	-0.25	0.802441	
Bravo clinid	Intercept	0.20	0.38	0.53	0.598770	26	0.30	0.43	0.71	0.477527	50
Gobioclinus dendriticus	Order	0.00	0.02	-0.22	0.827335		0.12	0.07	1.76	0.078212	
Bullseye puffer	Intercept	0.28	0.65	0.44	0.661254	12	0.60	0.59	1.02	0.309205	24
Sphoeroides annulatus	Order	0.01	0.03	0.22	0.829083		0.01	0.10	0.05	0.95902	
Chameleon wrasse	Intercept	4.23	1.93	2.19	0.028528	9	4.27	2.03	2.11	0.035024	18
Halichoeres dispilus	Order	0.06	0.03	1.72	0.085938		0.18	0.11	1.62	0.104518	
Cortez rainbow wrasse	Intercept	4.58	1.04	4.4	0.000011	15	3.40	0.88	3.86	0.000113	29
Thalassoma lucasanum	Order	-0.11	0.03	-3.5	0.000460		-0.03	0.09	-0.37	0.713771	
Eagle ray	Intercept	-5.38	3.82	-1.41	0.158958	1	0.62	2.43	0.25	0.799435	1
Aetobatus narinari	Order	0.22	0.27	0.81	0.419141		-0.79	0.94	-0.85	0.397174	
Flag cabrilla	Intercept	-2.40	0.43	-5.63	0.001000	17	-1.38	0.47	-2.95	0.003156	23
Epinephelus labriformis	Order	0.02	0.04	0.59	0.552298		0.06	0.12	0.53	0.599324	
Galapagos bullhead shark	Intercept	-699.80	332061.42	0.00	0.998319	1	-253.12	470628.30	0.00	0.999571	1
Heterodontus quoyi	Order	40.00	19072.48	0.00	0.998327		46.06	86811.83	0.00	0.999577	
Galapagos grunt	Intercept	-1.47	0.55	-2.67	0.007593	6	-1.58	0.79	-1.99	0.046188	8
Orthopristis forbesi	Order	0.00	0.05	-0.02	0.980965		0.37	0.19	1.91	0.055509	
Galapagos ringtail damselfish	Intercept	6.84	2.26	3.02	0.002521	9	5.69	2.36	2.41	0.016135	17
Stegastes beebei	Order	0.03	0.04	0.89	0.375651		0.16	0.12	1.32	0.185678	
Galapagos Seabream	Intercept	0.34	0.75	0.45	0.653361	3	-0.53	0.88	-0.60	0.548921	7
Archosargus pourtalesi	Order	-0.01	0.06	-0.17	0.865352		0.33	0.20	1.63	0.103143	
Galapagos shark	Intercept	-7.21	3.19	-2.26	0.024065	3	-1.37	1.40	-0.98	0.327356	3
Carcharhinus galapagensis	Order	0.34	0.21	1.64	0.100432		-0.07	0.37	-0.18	0.853274	
Galapagos sheepshead wrasse	Intercept	-1.64	1.78	-0.92	0.357165	1	-253.12	470628.30	0.00	0.999571	1
Semicossyphus darwini	Order	-0.15	0.23	-0.66	0.511200		46.06	86811.83	0.00	0.999577	
Galapagos triplefin blenny	Intercept	-2.65	0	-930.18	0.001000	11	-0.36	0.57	-0.64	0.522337	14
Lepidonectes corallicola	Order	0.07	0	24.72	0.001000		-0.1	0.14	-0.69	0.488474	
Giant hawkfish	Intercept	-1.32	0.47	-2.79	0.005201	8	-1.34	0.62	-2.19	0.028840	13
Cirrhitus rivulatus	Order	-0.06	0.05	-1.29	0.196009		0.06	0.15	0.39	0.699636	
Harlequin wrasse	Intercept	-592.04	337281.7	0.00	0.998599	1	-117.08	268839.08	0.00	0.999653	1
Choerodon fasciatus	Order	40.83	23269.99	0.00	0.998600		46.79	103459.15	0.00	0.999639	
Jewel moray	Intercept	-3.52	0.95	-3.71	0.000209	6	-1.25	0.91	-1.37	0.170569	6
Muraena lentiginosa	Order	0.09	0.08	1.25	0.212205		0.00	0.23	0.00	1.000000	
Kingangelfish	Intercept	-2.8	0.66	-4.26	0.000002	8	-0.29	0.7	-0.42	0.676893	8
Holacanthus passer	Order	0.06	0.06	1.02	0.306740		-0.18	0.19	-0.92	0.355855	
Loosetooth parrotfish	Intercept	-1.29	1.75	-0.74	0.460641	1	-4.93	4.61	-1.07	0.284486	1
Nicholsina denticulata	Order	-0.22	0.27	-0.81	0.419141		0.79	0.94	0.85	0.397174	
Marbled goby	Intercept	-0.22	0.37	-0.59	0.555023	27	-0.05	0.43	-0.11	0.911877	39
Gobio manchada	Order	-0.05	0.02	-2.43	0.015042		0.11	0.08	1.44	0.151206	
Marbled ray	Intercept	-6.62	4.95	-1.34	0.181138	1	-0.92	2.33	-0.39	0.69451	1
Taeniurops meyeni	Order	0.30	0.33	0.92	0.356654		-0.21	0.66	-0.32	0.751098	
Mexican hogfish	Intercept	-2.00	0.39	-5.15	0.001000	20	-0.81	0.51	-1.57	0.117022	33
Bodianus diplotaenia	Order	0.07	0.03	2.68	0.007294		0.06	0.09	0.74	0.456656	
Mojarra grunt	Intercept	-1.93	0.79	-2.45	0.014254	4	-1.01	1.03	-0.98	0.329466	4
Haemulon scudderii	Order	-0.03	0.08	-0.42	0.674035		0.03	0.26	0.13	0.895424	
Mullet snapper	Intercept	-12.06	8.31	-1.45	0.146901	1	-2.92	2.68	-1.09	0.276547	1
Lutjanus aratus	Order	0.71	0.52	1.37	0.169709		0.59	0.62	0.95	0.344115	
Pacific dog snapper	Intercept	-12.15	6.75	-1.8	0.071946	1	0.92	2.33	0.39	0.694510	1
Lutjanus novemfasciatus	Order	0.90	0.50	1.81	0.070510		0.21	0.66	0.32	0.751098	
Pacific spotfin mojarra	Intercept	0.77	0.80	0.97	0.333770	9	1.32	0.64	2.05	0.040643	19
Eucinostomus dowii	Order	0.13	0.04	3.47	0.000528		0.17	0.11	1.58	0.114509	
Panamic fanged blenny	Intercept	-0.92	0.42	-2.19	0.028323	24	-0.72	0.38	-1.91	0.056012	46
Ophioblennius steindachneri	Order	0.01	0.02	0.63	0.526563		0.15	0.08	1.94	0.052235	
Panamic sergeant major	Intercept	2.20	2.51	0.88	0.380888	8	2.07	1.14	1.81	0.070653	10
Abudefduf troschelii	Order	0.31	0.07	4.61	0.000004		0.46	0.17	2.69	0.007223	
Razor surgeonfish	Intercept	-1.36	0.51	-2.65	0.007970	10	-0.26	0.47	-0.55	0.580781	21
Prionurus laticlavius	Order	0.06	0.03	1.63	0.102885		0.01	0.11	0.05	0.957375	
Reef cornetfish	Intercept	-0.92	0.50	-1.84	0.066457	6	-1.73	0.88	-1.95	0.050716	9
Fistularia commersonii	Order	-0.05	0.05	-1.11	0.266538		0.38	0.19	1.98	0.047591	
Sabertooth blenny	Intercept	0.79	0.38	2.07	0.038441	23	1.39	0.51	2.76	0.005756	45
Plagiotremus azaleus	Order	-0.01	0.02	-0.29	0.774761		-0.13	0.07	-1.77	0.077200	
Spinster wrasse	Intercept	1.34	0.57	2.35	0.018863	18	1.41	0.57	2.47	0.013542	32
Halichoeres nicholsi	Order	0.04	0.03	1.47	0.141888		0.18	0.09	1.93	0.052991	
Spotted cabrilla	Intercept	-699.8	332061.42	0.00	0.998319	1	-253.12	470628.30	0.00	0.999571	1
Epinephelus analogus	Order	40.00	19072.48	0.00	0.998327		46.06	86811.83	0.00	0.999577	
Stonescorpionfish	Intercept	0.32	0.81	0.39	0.697075	4	-0.22	1.14	-0.19	0.849113	4
Scorpaena mystes	Order	-0.36	0.13	-2.77	0.005677		-0.11	0.27	-0.41	0.684013	
Striped mullet	Intercept	-15.29	14.39	-1.06	0.287992	1	-4.93	4.61	-1.07	0.284486	1
Mugil cephalus	Order	0.84	0.86	0.98	0.325896		0.79	0.94	0.85	0.397174	
Three banded butterfly fish	Intercept	-0.73	0.74	-0.99	0.321277	8	0.04	0.76	0.06	0.953188	12
Chaetodon humeralis	Order	-0.01	0.04	-0.24	0.812474		0.01	0.15	0.08	0.940187	
Throatspotted blenny	Intercept	-1.59	0.88	-1.81	0.069802	3	0.19	1.16	0.17	0.867320	3
Malacoctenus tetranemus	Order	-0.08	0.09	-0.85	0.397326		-0.35	0.33	-1.05	0.293921	
Tiger snake eel	Intercept	-2.13	0.60	-3.55	0.000385	7	-0.57	0.66	-0.87	0.386257	10
Myrichthys maculosus	Order	0.02	0.05	0.43	0.665442		-0.10	0.17	-0.59	0.557025	
triplefin blenny	Intercept	0.71	2.30	0.31	0.756327	1	0.62	2.43	0.25	0.799435	1
Lepidonectes corallicola	Order	-0.84	0.86	-0.98	0.325896		-0.79	0.94	-0.85	0.397174	
White mullet	Intercept	-0.38	1.35	-0.28	0.779185	1	-5.24	4.16	-1.26	0.207754	1
Mugil curema	Order	-0.23	0.2	-1.17	0.243282		1.14	0.93	1.23	0.217323	
White salema	Intercept	-2.41	0.64	-3.75	0.00018	11	-0.38	0.72	-0.52	0.603209	13
Xenichthys agassizii	Order	0.08	0.04	2.12	0.033621		0.02	0.14	0.15	0.880997	
Wounded wrasse	Intercept	-1.65	0.46	-3.59	0.000336	10	0.00	0.73	0.01	0.994750	10
Halichoeres chierchiae	Order	0.01	0.04	0.13	0.893250		-0.06	0.15	-0.39	0.698345	
Yellowtail damselfish	Intercept	4.01	1.21	3.33	0.00088	13	4.96	0.00	5765.33	0.001000	30
Chrysiptera parasema	Order	0.01	0.03	0.43	0.664362		-0.19	0.00	-220.48	0.001000	

Table C.2: Output of binomial mixed models with Presence/Absence as response variable, Order of the observation as fixed effect and Transect as random effect. The coefficients (Estimate), standard error (SE), z-value and p-value are given for each species. Significant positive effects ($p<0.05$) are indicated in green, Significant negative effects ($p<0.05$) are indicated in red. n represents the number of series of 6 or 18 repeats during which at least one individual of the concerning species was detected.

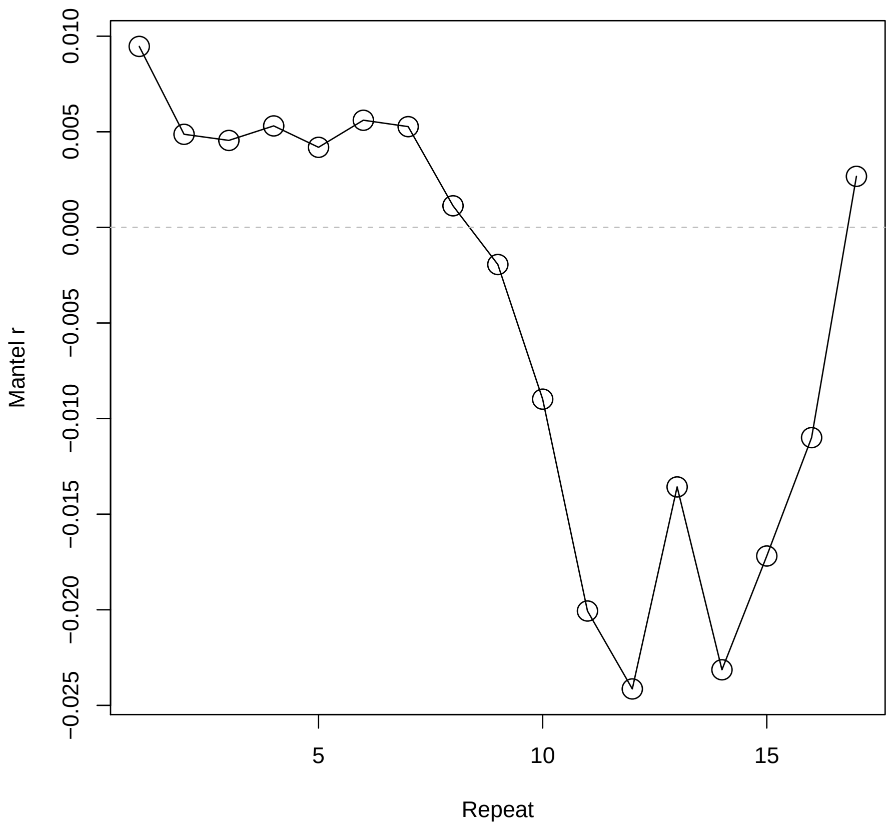

Figure C.1: Partial mantel correlogram for temporal auto-correlation of the observed structure of fish assemblages. The effect of the sampling units themselves was partialled out. No significant temporal auto-correlations were found.

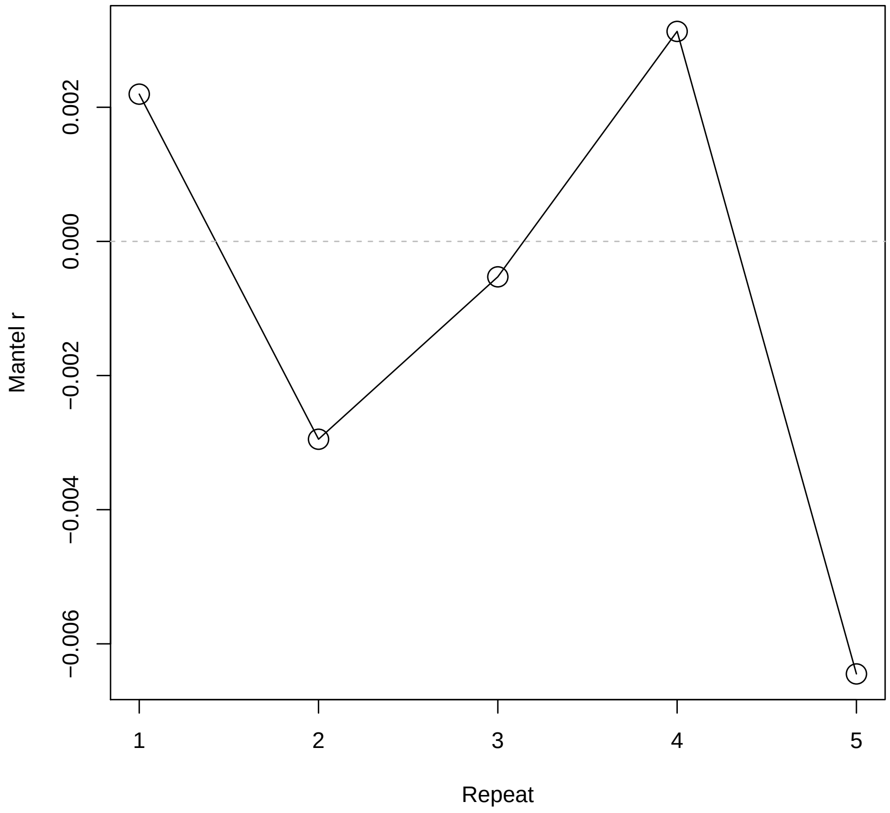

Figure C.2: Partial mantel correlogram for temporal auto-correlation of the observed structure of fish assemblages. The effect of the sampling units and observers themselves was partialled out. No significant temporal auto-correlations were found.

Appendix D. Indicator species

Applied ecological studies often assess the potential of different species to serve as indicators for water quality. For each island, two potential indicator species were determined using CAP analysis based on the group-

ing factor Location. The species with the highest Spearman correlation to the CAP axes (m=4) were retained (Table D.1), as they are representative for observed differences among groups. The full 50 meter dataset was split up for the two islands and separate CAP analyses were performed. Data was fourth-root transformed and Bray-Curtis dissimilarities were calculated. We decided to perform one CAP analysis per island as the differences in species between the islands may be more related to biogeography and dispersal limitations than to environmental conditions which dictate the water quality. The Spinster Wrasse (*Halichoeres nicholsi*) and Sabertooth Blenny (*Plagiotremus azaleus*) were selected for Santa Cruz, while the Bravo Clinid (*Gobioclinus dendriticus*) and Panamic Fanged Blenny (*Ophioblennius steindachneri*) were selected for Floreana. It should be noted that these species might not be actual indicator species for water quality, as the constrained ordination was performed on an artificial grouping factor, rather than on actual environmental gradients.

	Santa Cruz		Floreana	
	Spinster Wrasse	Sabertooth Blenny	Bravo Clinid	Panamic Fanged Blenny
CAP1	-0.815	-0.593	-0.645	0.665
CAP2	0.186	-0.414	0.426	0.420
CAP3	-0.071	-0.270	-0.025	-0.223
CAP4	0.172	0.093	-0.058	-0.224

Table D.1: Selection of potential indicator species using CAP discriminant analysis with Location as grouping factor. CAP analysis was performed for each island. Four CAP axes were determined as optimal and correlations of each species with the axes were determined. The species with the highest correlations were retained and given in the table.

Appendix E. Species Accumulation Curves (SACs)

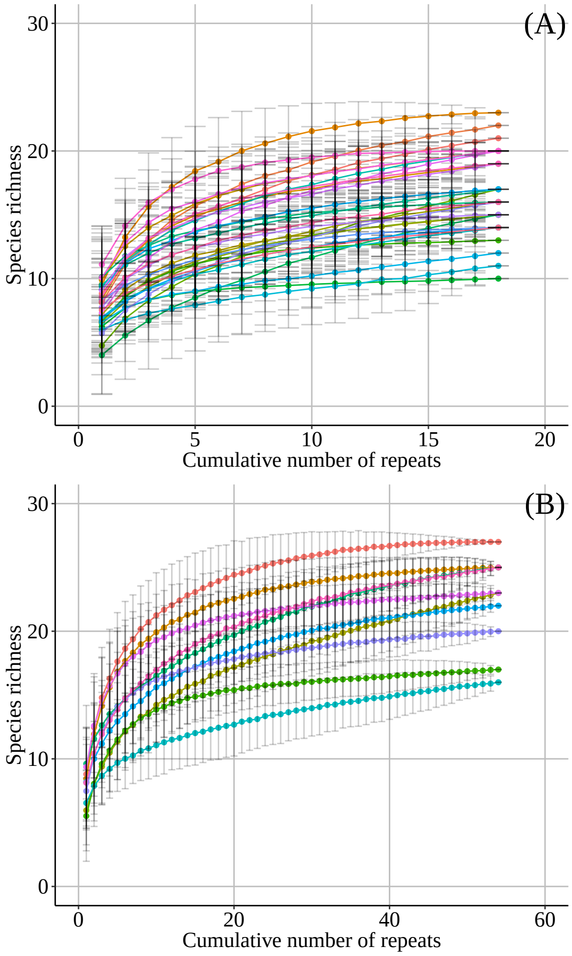

Figure E.1: Species Accumulation Curves (SACs) depicting the cumulative species richness in function of the cumulative number of repeats. Permutations or randomizations without replacement (n=10^4) of the repeats were used to determine the mean and standard deviation. The error bars represent the 95% confidence interval. In (A) the repeats were pooled within Transect, resulting in one SAC per transect (n=18). In (B) the repeats were pooled within Location, resulting in one SAC per location (n=54).

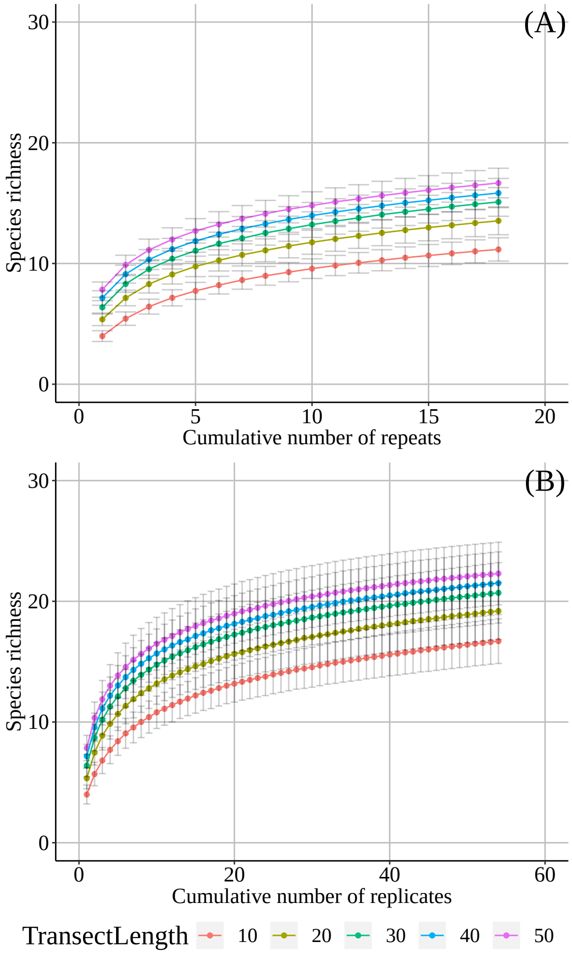

Figure E.2: Species Accumulation Curves (SACs) depicting the cumulative species richness in function of the cumulative number of repeats for different transect lengths. Permutations or randomizations without replacement (n=10^4) of the repeats were used to determine the mean and standard deviation. The error bars represent the 95% confidence interval. In (A) the repeats were pooled within Transect (n=18). In (B) the repeats were pooled within Location (n=54).

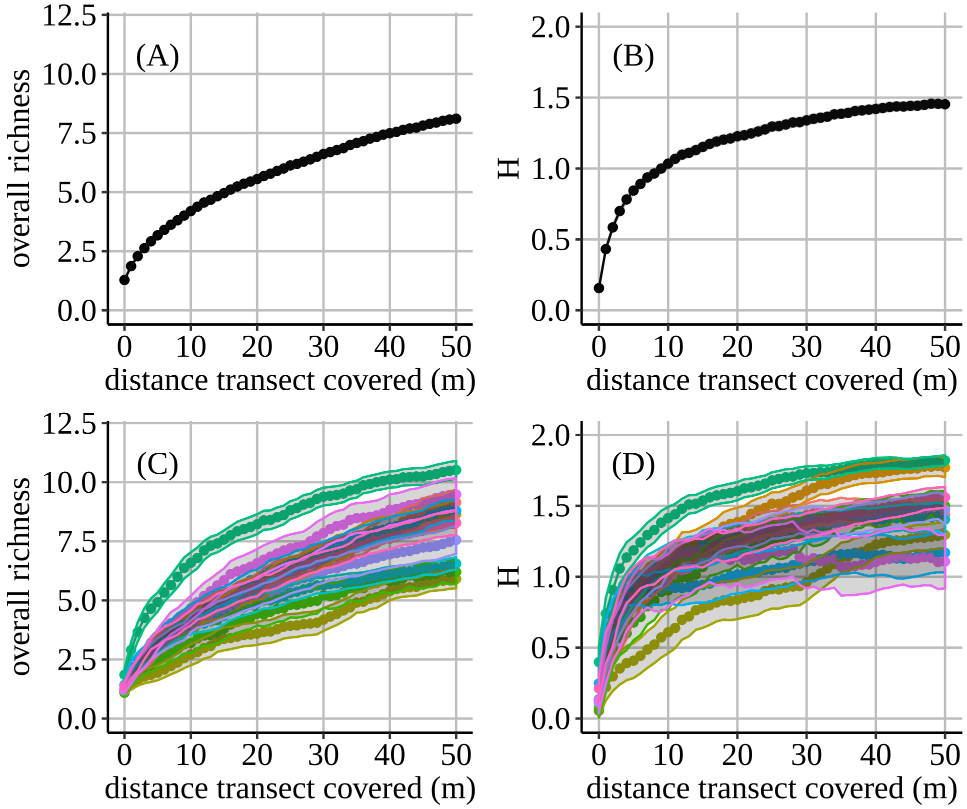

Figure E.3: Species Accumulation Curves (SACs) depicting the cumulative species density and cumulative Shannon diversity (H) in function of the transect distance. The error bars represent the 95% confidence interval. In (A) and (B) the average cumulative species density and Shannon diversity of all observations (n=540) are depicted respectively. In (C) and (D) a distinction is made between the 10 locations (n=54).

Appendix F. Generalized linear mixed models

Species	ICC	10 meters	20 meters	30 meters	40 meters	50 meters
Spinster wrasse	$ICC_{Transect}$	0.107	0.021	0.041	0.05	0.054
	$ICC_{Location}$	0.651	0.188	0.294	0.383	0.494
	$ICC_{Observer}$	0.007	0.001	0.006	0.009	0.010
	$ICC_{Observer:Location}$	0.025	0.007	0.009	0.007	0.008
	$ICC_{Observer:Transect}$	0.000	0.000	0.000	0.001	0.003
	ICC_{Total}	0.790	0.217	0.350	0.450	0.569
	$ICC_{Sampling}$	0.235	0.790	0.659	0.558	0.442
Sabertooth blenny	$ICC_{Transect}$	0.108	0.106	0.131	0.165	0.188
	$ICC_{Location}$	0.683	0.677	0.765	0.583	0.593
	$ICC_{Observer}$	0.000	0.000	0.000	0.000	0.000
	$ICC_{Observer:Location}$	0.003	0.000	0.000	0.000	0.000
	$ICC_{Observer:Transect}$	0.092	0.041	0.046	0.043	0.026
	ICC_{Total}	0.886	0.824	0.942	0.791	0.807
	$ICC_{Sampling}$	0.209	0.217	0.104	0.252	0.219
Bravo clinid	$ICC_{Transect}$	0.362	0.178	0.120	0.098	0.099
	$ICC_{Location}$	0.184	0.162	0.153	0.159	0.163
	$ICC_{Observer}$	0.055	0.047	0.019	0.027	0.013
	$ICC_{Observer:Location}$	0.188	0.223	0.268	0.207	0.184
	$ICC_{Observer:Transect}$	0.041	0.062	0.040	0.043	0.043
	ICC_{Total}	0.830	0.672	0.600	0.534	0.502
	$ICC_{Sampling}$	0.399	0.613	0.708	0.716	0.725
Panamic fanged blenny	$ICC_{Transect}$	0.000	0.070	0.184	0.135	0.158
	$ICC_{Location}$	0.000	0.158	0.414	0.388	0.469
	$ICC_{Observer}$	0.000	0.002	0.010	0.012	0.010
	$ICC_{Observer:Location}$	0.000	0.000	0.000	0.000	0.005
	$ICC_{Observer:Transect}$	0.000	0.000	0.025	0.024	0.022
	ICC_{Total}	0.000	0.230	0.633	0.559	0.664
	$ICC_{Sampling}$	1.0000	0.770	0.392	0.465	0.363

Table F.1: Model output (ICC (Intraclass Correlation Coefficient) of the random effects, total ICC explained by the factors of the model and ICC associated with the sampling variability) for generalized linear mixed models (zero-inflated Conway-Maxwell-Poisson) with either the count of the Spinster wrasse (*Halichoeres nicholsi*), Sabertooth blenny (*Plagiotremus azaleus*), Bravo clinid (*Gobioclinus dendriticus*) and Panamic fanged blenny (*Ophioblennius steindachneri*) as response, Location and Transect as nested random effects and Observer as crossed random effect. Transect lengths of 10, 20, 30, 40 and 50 meters were assessed.

Species	ICC *Transect*	ICC *Location*	ICC *Observer*	ICC *Observer: Location*	ICC *Observer: Transect*	ICC *Total*	ICC *Sampling*
Amarillo snapper	0.4584	0.5193	0.0000	0.0000	0.0000	0.9777	0.0223
Banded wrasse	0.0460	0.0303	0.0000	0.4178	0.0932	0.5873	0.9237
Black-striped salema	0.0328	0.0005	0.0000	0.0014	0.0001	0.0348	0.9985
Blacktip cardinalfish	0.0195	0.0000	0.0000	0.0005	0.0015	0.0215	0.9805
Blue and gold snapper	0.8299	0.0000	0.0000	0.0232	0.0000	0.8531	0.1701
Bluechin parrotfish	0.0052	0.0000	0.0000	0.0000	0.019	0.0242	0.9948
Bravo clinid	0.0837	0.0328	0.0000	0.2844	0.0382	0.4391	0.8835
Bullseye puffer	0.3082	0.3218	0.0196	0.0093	0.0238	0.6827	0.3504
Chameleon wrasse	0.0121	0.1224	0.0000	0.0047	0.0085	0.1477	0.8655
Cortez rainbow wrasse	0.0746	0.0621	0.0034	0.0026	0.0130	0.1557	0.8599
Flag cabrilla	0.0999	0.2053	0.0000	0.0421	0.3869	0.7342	0.6948
Galapagos grunt	0.0000	0.0000	0.0000	0.2610	0.0483	0.3093	1.0000
Galapagos ringtail damselfish	0.1248	0.3435	0.0006	0.0156	0.0246	0.5091	0.5311
Galapagos seabream	0.8974	0.0000	0.0000	0.0000	0.0187	0.9161	0.1026
Galapagos triplefin blenny	0.0000	0.0000	0.0000	0.5883	0.3947	0.9830	1.0000
Giant hawkfish	0.7725	0.0000	0.0000	0.2037	0.0000	0.9762	0.2275
Jewel moray	0.0001	0.0003	0.0000	0.0030	0.9937	0.9971	0.9996
Marbled goby	0.0654	0.0765	0.0000	0.2367	0.0270	0.4056	0.8581
Mexican hogfish	0.2232	0.0000	0.1765	0.0000	0.3115	0.7112	0.6003
Mojarra grunt	0.0000	0.0000	0.0000	0.0829	0.8992	0.9821	1.0000
Mullet snapper	0.0000	0.0000	0.0000	0.0000	0.9914	0.9914	1.0000
Pacific dog snapper	0.0000	0.0000	0.0000	0.0000	0.9899	0.9899	1.0000
Pacific spotfin mojarra	0.0320	0.0109	0.0000	0.0037	0.0024	0.0490	0.9571
Panamic fanged blenny	0.1255	0.4897	0.0000	0.0300	0.0235	0.6687	0.3848
Panamic sergeant major	0.0299	0.0739	0.0010	0.0000	0.0316	0.1364	0.8952
Razor surgeonfish	0.0191	0.0000	0.0000	0.0000	0.0028	0.0219	0.9809
Reef cornetfish	0.0186	0.0362	0.0000	0.0000	0.9258	0.9806	0.9452
Sabertooth blenny	0.1248	0.2837	0.0017	0.0368	0.0412	0.4882	0.5898
Spinster wrasse	0.1045	0.4102	0.0000	0.0207	0.0174	0.5528	0.4853
Striped mullet	0.0543	0.0184	0.8041	0.0277	0.0753	0.9798	0.1232
Three banded butterflyfish	0.6487	0.1585	0.0208	0.0000	0.0980	0.9260	0.1720
Tiger snake eel	0.0000	0.1814	0.0000	0.0000	0.4897	0.6711	0.8186
White mullet	0.0000	0.0000	0.0000	0.0000	0.9961	0.9961	1.0000
White salema	0.0535	0.0000	0.0000	0.2214	0.0020	0.2769	0.9465
Wounded wrasse	0.0679	0.0000	0.0000	0.4247	0.0000	0.4926	0.9321
Yellowtail damselfish	0.0055	0.0045	0.0002	0.0019	0.0001	0.0122	0.9898

Table F.2: ICC values of generalized linear mixed models (zero-inflated Conway-Maxwell-Poisson) with the counts of the different observed species as response, Island as fixed effect, Location and Transect as nested random effects and Observer as crossed random effect. The data originating from the full 50-meter transect was used.

Appendix G. Design error

Observations pooled	Dissimilarity	Transect length (meters) 10	20	30	40	50
1	NA	0.007	0.000	0.000	0.000	0.000
2	NA	0.000	0.000	0.000	0.000	0.000
3	NA	0.000	0.000	0.000	0.000	0.000
4	NA	0.000	0.000	0.000	0.000	0.000
5	NA	0.000	0.000	0.000	0.000	0.000
6	NA	0.000	0.000	0.000	0.000	0.000
1	0	0.0908	0.014	0.014	0.007	0.000
2	0	0.0094	0.000	0.000	0.000	0.000
3	0	0.0026	0.000	0.000	0.000	0.000
4	0	0.0013	0.000	0.000	0.000	0.000
5	0	0.000	0.000	0.000	0.000	0.000
6	0	0.000	0.000	0.000	0.000	0.000
1	1	4.2488	0.9783	0.3494	0.0699	0.000
2	1	0.1689	0.0177	0.0023	0.000	0.000
3	1	0.0065	0,001	0.000	0.000	0.000
4	1	0.000	0.000	0.000	0.000	0.000
5	1	0.000	0.000	0.000	0.000	0.000
6	1	0.000	0.000	0.000	0.000	0.000

Table G.1: Effect of different pooling scenarios (1 to 6 observations with steps of 1) within locations for different transect lengths on the percentage of undefined (NA) dissimilarity values and perfectly similar (0) and dissimilar (1) observations. Monte Carlo simulations (n=10000) were applied to pool the observations.

Observations pooled	Dissimilarity	Transect length (meters)				
		10	20	30	40	50
1	NA	0.0023	0.000	0.000	0.000	0.000
2	NA	0.000	0.000	0.000	0.000	0.000
3	NA	0.000	0.000	0.000	0.000	0.000
4	NA	0.000	0.000	0.000	0.000	0.000
5	NA	0.000	0.000	0.000	0.000	0.000
6	NA	0.000	0.000	0.000	0.000	0.000
1	0	0.0116	0.0023	0.0047	0.0023	0.000
2	0	0.0019	0.000	0.000	0.000	0.000
3	0	0,001	0.000	0.000	0.000	0.000
4	0	0.000	0.000	0.000	0.000	0.000
5	0	0.000	0.000	0.000	0.000	0.000
6	0	0.000	0.000	0.000	0.000	0.000
1	1	0.2399	0.0349	0.014	0.000	0.000
2	1	0.0141	0,001	0.000	0.000	0.000
3	1	0.0017	0.000	0.000	0.000	0.000
4	1	0.000	0.000	0.000	0.000	0.000
5	1	0.000	0.000	0.000	0.000	0.000
6	1	0.000	0.000	0.000	0.000	0.000

Table G.2: Effect of different pooling scenarios (1 to 6 observations with steps of 1) within transects for different transect lengths on the percentage of undefined (NA) dissimilarity values and perfectly similar (0) and dissimilar (1) observations. Monte Carlo simulations (n=10000) were applied to pool the observations.

1031 Appendix H. PERMANOVA

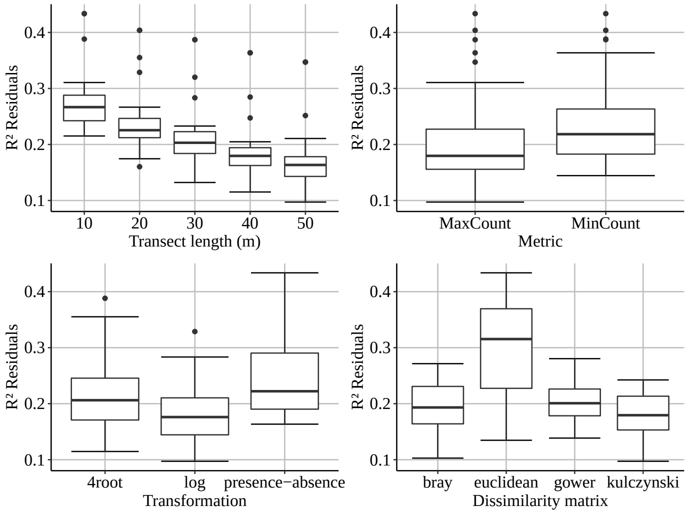

Figure H.1: R^2 of the residuals of the PERMANOVA models with Island as fixed factor, Location and Transect as nested random factors and Observer as crossed random factor. $R^2_{Residuals}$ is defined here as the ratio of the sum of squares of the error over the total sum of squares. Different parameters were assessed including the transect length (10, 20, 30, 40 and 50 meters), metric (MaxCount and MinCount), transformation (4root, logarithm and presence-absence) and the method to calculate the dissimilarity matrix (Bray-Curtis, Euclidean, Gower and Kulczynski).

Source	df	MS	pseudo-F	p-value	Unique perms
Island	1	445960	15.17	0.001	999
Observer	2	3913	1.52	0.248	60
Location	8	26981	2.90	0.001	994
Island X Observer	2	2578	1.06	0.400	999
Transect	20	7222	7.32	0.001	997
Location X Observer	16	2437	2.47	0.001	997
Transect X Observer	40	987	3.63	0.001	998
Residuals	450	272			

Table H.1: PERMANOVA testing for effect of Island as fixed factor, Location and Transect as nested random factors and Observer as crossed random factor on the structure of the observed fish assemblages. PERMANOVA done on Bray-Curtis dissimilarity matrices (fourth-root transformation).

	10 meters	20 meters	30 meters	40 meters	50 meters
R^2_{Island}	0.420**	0.418**	0.428**	0.429**	0.437**
$R^2_{Observer}$	0.006	0.005	0.006	0.007	0.008
$R^2_{Location}$	0.145**	0.165**	0.178**	0.196**	0.212**
$R^2_{Island:Observer}$	0.003	0.004	0.004	0.004	0.004
$R^2_{Transect}$	0.145**	0.132**	0.140**	0.144**	0.142**
$R^2_{Location:Observer}$	0.022*	0.028*	0.027*	0.027*	0.031*
$R^2_{Transect:Observer}$	0.045**	0.046**	0.042**	0.040**	0.039**
$R^2_{VideoAnalyst}$	0.003	0.007	0.007	0.008	0.008
$R^2_{Residuals}$	0.239	0.193	0.166	0.144	0.120

Table H.2: Goodness-of-fit (R^2) estimates for PERMANOVA models based on Bray-Curtis dissimilarities (fourth-root transformation) with Island as fixed factor, Location and Transect as nested random factors and Observer and Video analyst as crossed random factors for transect lengths of 10, 20, 30, 40 and 50 meters.

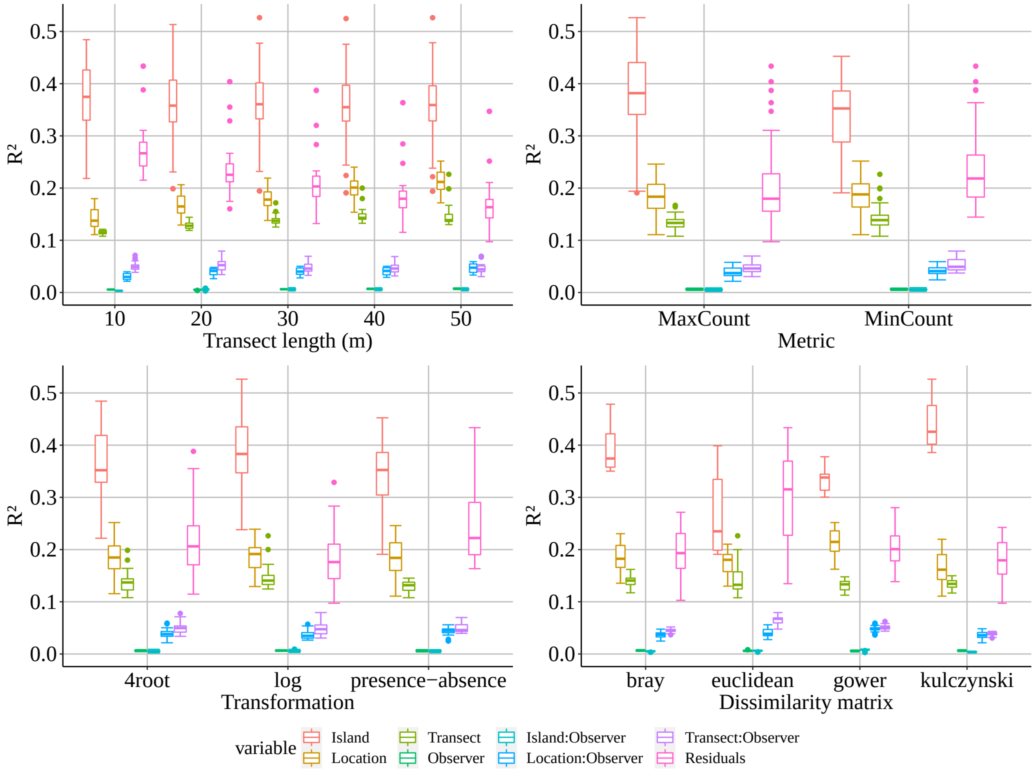

Figure H.2: R^2 of the different factors of the PERMANOVA models with Island as fixed factor, Location and Transect as nested random factors and Observer and its interactions with the other factors as crossed random factors. R^2 is defined here as the ratio of the sum of squares over the total sum of squares. Different parameters were assessed including the transect length (10, 20, 30, 40 and 50 meters), metric (MaxCount and MinCount), transformation (4root, logarithm and presence absence data) and the method to calculate the dissimilarity matrix (Bray-Curtis, Euclidean, Gower and Kulczynski).

Appendix I. Precision

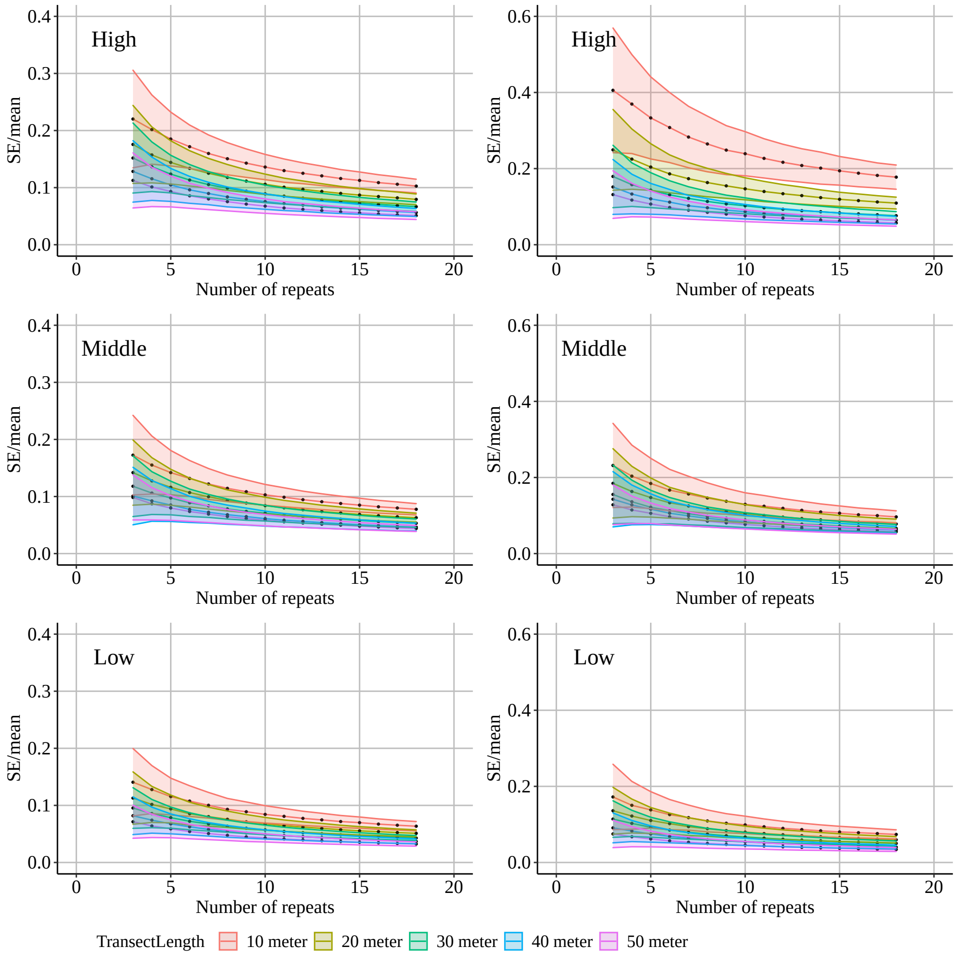

Figure I.1: Precision estimates of species density (left) and Shannon diversity (right) with precision defined as the inverse of the standard error over the mean. The 30 transects were divided in 3 equally sized groups with different levels of turbidity (High, Middle and Low). Different transect lengths were considered ranging from 10, 20, 30, 40 to 50 meters. Monte Carlo simulations ($n=10^4$) were applied to determine the precision per transect. The average precision over all transects is visualized. The error bars represent the 95% confidence intervals which were constructed using the pooled standard deviation of the estimates.

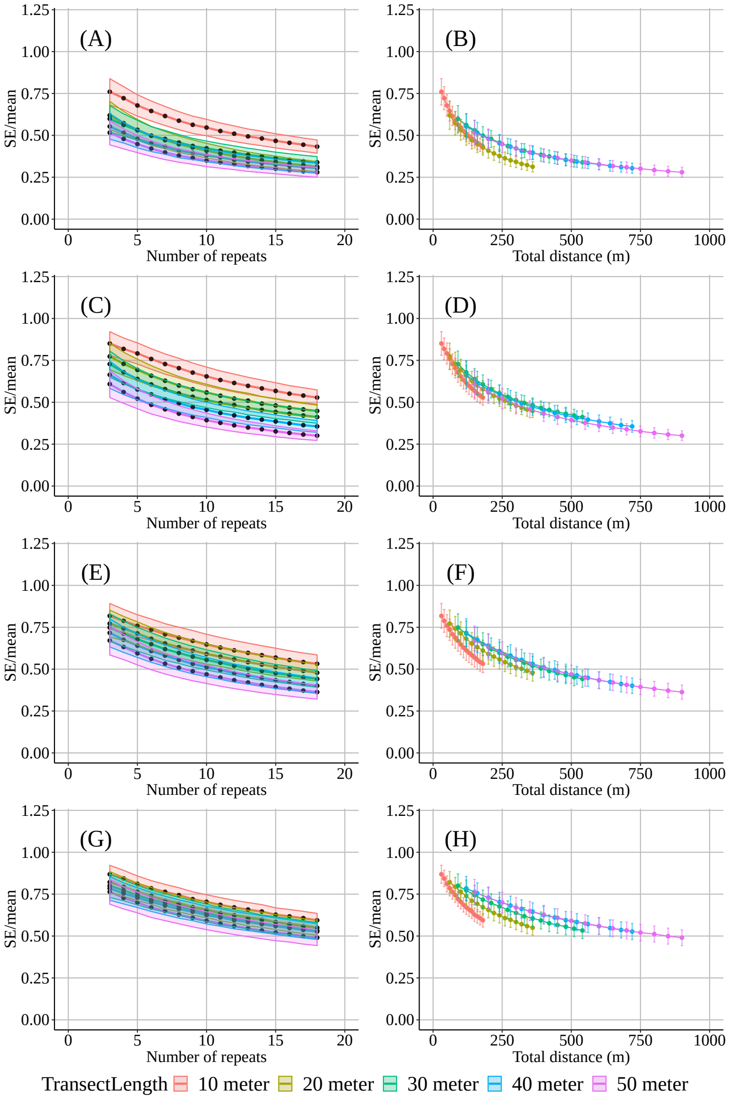

Figure I.2: Precision estimates of abundances (N) of different potential indicator species in function of the number of repeats (A,C,E,G) and in function of the total swim distance (B,D,F,H). Precision is defined as the inverse of the standard error over the mean. Total swim distance is defined as the number of repeats multiplied with the transect length. The potential indicator species were Spinster Wrasse (*Halichoeres nicholsi*); A, B, Sabertooth Blenny (*Plagiotremus azaleus*); C, D, Bravo Clinid (*Gobioclinus dendriticus*); E, F, and Panamic Fanged Blenny (*Ophioblennius steindachneri*)); G, H. Different transect lengths were considered ranging from 10, 20, 30, 40 to 50 meters. Monte Carlo simulations ($n=10^4$) were applied to determine the precision per transect. The average precision over all transects is visualized. The error bars represent the 95% confidence intervals which were constructed using the pooled standard deviation of the estimates.
