# Supplementary material for: Sampling errors and variability in video transects for assessment of reef fish assemblage structure and diversity
Source: PLoS One. 2022 Jul 25;17(7):e0271043. doi: 10.1371/journal.pone.0271043 (PMC9312474; doi:10.1371/journal.pone.0271043)
Supplement: Attachment — Submitted filename: Rebuttal_letter_May2022_PlosOne.pdf [file pone.0271043.s028.pdf]

Rebuttal letter

We thank the reviewers for their helpful comments and interest in the topic. The remarks have contributed to the outcome of the reviewed manuscript and have, in our opinion, increased the quality of its content and structure. In the next section we will clarify how we integrated the comments and suggestions in this reviewed version of the manuscript.

Reviewer #1:

recommended changing "should" in line 6 which was done by the authors.

Introduction:

Line 5:

What environmental and ecological changes are referred to here? The prior sentence doesn't quite reference changes just physical and chemical water conditions that are known to be affected by anthropogenic pressures. What are "these changes?" Remove "these" from line 4.

Answer: “these” was removed.

Line 34:

Remove the comma and add "thereby" before "providing a more...."

Answer: comma removed and “thereby” added.

Line 35:

"Baited stationary cameras" is written in the prior sentence but the authors refer to the camera systems as "its" in line 35. Replace "its" with something like "However, due to the point-based nature of these camera systems, it remains difficult....."

Answer: We corrected it as such.

Line 74 to 78: The questions seem to just be listed here with little connection to the paragraph above. Why not change the wording to "Although repetitions of transects are not common in the literature, they were necessary here to provide answers to the following research questions, which are of interest for both non-repetitive and repetitive studies:"

Answer: We corrected it as such.

Discussion:

Line 443: Remove "two curious species." This statement makes the assumption that the fish were in fact curious which is something that cannot be proven.

Answer: We have rewritten it as “two species which are known to not be frightened easily” and now also refer to literature.

Line 446: Replace curious with "non-scared" fish. Being attracted to the divers doesn't mean the fish are actually curious.

Answer: We corrected it as such.

Line 462 to line 469: Could the temporal auto-correlation, fish displacement, and observer biases be avoided or greatly reduced by sampling transects over a series of days not one after another on the same day? The sampling events would still be repeats.

Answer: The issue there might be that the time between observations becomes so large that the assemblages are completely different, not because of issues related to the observation method but simply because of the movement of animals. The question is then whether two observations in two subsequent days can be considered repeats or not.

Reviewer #2:

The authors have made some improvements in the text (some are not highlighted in the resubmitted document), such as including more references and adjusting word choice and clarifications. Unfortunately, the authors have not addressed other comments, and thus, there are still some inaccuracies that need attention before acceptance for publication. Please see comments below with reference to line numbers of the new submitted version.

Abstract (no numbered lines)

Conclusions/significance can be improved. "The results confirm the suitability of the technique to study reef fish assemblages," This was not the objective of the study, so this was not proved.

Answer: We included this now in the explicit description of the aim of the study: **L53-55**: 'The aim of this study was therefore to provide a detailed assessment of the observer effects, observer bias, sampling variability, sampling design and overall suitability of video transects to study reef fish assemblages.'

Introduction

Lines 33-67. Static videos (RUV) are not adequate for extensive sampling areas but baited videos can improve this. These ideas are incorrect. RUV can be designed to cover extensive areas. Western and Eastern Australia is an example of this. The plume-effect of baited videos is actually a problem, since it can attract fish species from kilometers away that normally would not be in the sampling location, modifying natural fish assemblages and habitat associations. Also, it is well known that fish densities and habitat heterogeneity can be accounted for in video analyses when designed appropriately (e.g. benthobox). Authors need to be careful with these kind of statements. I recommend to modify accordingly.

Answer: We rephrased this: **L31-38**: "Nevertheless, stationary cameras often only provide a limited view of the study area as only small patches are monitored, and are therefore of limited use when a rapid, yet spatially extensive survey of a study area has to be done \cite{Willis2000}. Baited stationary cameras remediate this limitation by attracting nearby fish thereby providing a more representative assessment of the local fish assemblages \cite{Harvey2007}. However, due to the point-based nature of these camera-systems, it remains difficult to accurately account for habitat heterogeneity and assess habitat preferences (especially of less mobile species) \cite{Andradi-Brown2016,Willis2000}."

Importantly, there has not been an improvement regarding the background information in the introduction to layout the knowledge gap being addressed by the study (Lines 52-65). Similarly, the explanation of the objectives remained unchanged. Thus, as it currently stands, the novel contribution of the study is not clear. Authors need to make this more explicit. This highlights the importance of the study more and also helps the readership to understand what the study is about easily. Aiming to evaluate "how important" is something, is not scientifically clear. The answer to this kind of questions would be "not important, important, mildly important, very important, etc" but we want quantitative answers. So it must be said exactly what you will test.

Answer: We described the aim of the study early on in the study now: **L53-55**: "The aim of this study was therefore to provide a detailed assessment of the observer effects, observer bias, sampling variability,

sampling design and overall suitability of video transects to study reef fish assemblages." The need to address this aim is also given in the paragraph before, by highlighting the limited number of studies focusing on only several of the methodological aspects that are targeted here. We have also added the following to highlight the need of this kind of research: **L46-50**: "Although studies comparing different video and other visual census techniques are becoming more frequent \cite{Andradi-Brown2016,Langlois2010,Tessier2013,Watson2005,Watson2010,Wilson2018}, few have focused on the methodological aspects of video transects, such as observer bias, design errors, sampling variability, counting metrics, data types and data transformations \cite{Bernard2013,Goetze2019}."

L 77. "Counting metric, data type and transformation" is referred in the third question/objective but this has not been mentioned at all in the background. If its in the objectives it must be explained why beforehand.

Answer: We have added this to the text: **L46-55**:"Although studies comparing different video and other visual census techniques are becoming more frequent \cite{Andradi-Brown2016,Langlois2010,Tessier2013,Watson2005,Watson2010,Wilson2018}, few have focused on the methodological aspects of video transects, such as observer bias, design errors, sampling variability, counting metrics, data types and data transformations \cite{Bernard2013,Goetze2019}. Video transects are often considered a simple extension of visual census transects, although the ability to store and standardize video observations (see \nameref{ap:add:applic}) justifies a specific methodological assessment of the technique. The aim of this study was therefore to provide a detailed assessment of the aforementioned methodological aspects and overall suitability of video transects to study reef fish assemblages." We also included a more detailed description of the meaning/interpretation of data transformations: **L274-279**: 'In addition, the considered transformation of abundance data to presence-absence data entails a broader discussion regarding the way data should be collected (i.e. identification and counting versus only identification respectively).' And **L591-595**: 'Although neglecting the abundance of a species scored worst in terms of goodness-of-fit, the difference between MinCount and presence-absence data was rather small. Therefore, in this specific case, if one wanted to reduce the time required for video analysis, the presence-absence instead of the abundance data could have been used, without much information being lost.' We do think however that the issue of data transformation does not fit well in the introduction as it would negatively affect the structure. If we would discuss all methodological aspects in the introduction in detail it would negatively affect the clarity of the introduction. We do, however, now also mention it explicitly in the aim of the study in the introduction: **L56-58**.

Methods

Table 1 (glossary) remains without legend or reference in the text.

Answer: We have added a caption and referred to the glossary when first using the terms that are explained in the glossary.

L83. Habitat description is missing (was all the sampling done in the same type of habitat?).

Answer: The habitat description was added: L91-93: "At ten locations, fixed transects were used to assess reef fish assemblages in rocky habitats close to the coast."

L86. I cannot see attachments with supplementary materials (e.g. Fig. B1 map).

Answer: These attachments were uploaded, but are perhaps not available to the reviewers for some reason.

L98. Why did you choose to survey the transect six consecutive times (why not less or more)?

Answer: We have now added this information: **L106-111**: 'To quantify observer bias and sampling variability, a sufficiently high number of repeats was required, yet the maximal number of observations per day was limited by light availability and fatigue of the observers. Balancing these requirements resulted in each of the transects being recorded six consecutive times with single GoPro cameras (GoPro Hero 5 Black, 1080p, 60fps, wide FOV) by three different observers equipped with a mask and snorkel.'

L102. How widely used is this technique (s-transect) around the world?

Answer: It is widely used because of its mentioned advantages. We added this L113: "The observers covered the transects in a browsing fashion, similar to the widely used S-type transects introduced by \cite{Pelletier2011}."

Answer
L110. Time between surveys of different observers was 1 minute, which according to other studies is too short to avoid effects between observers, you should account/discuss this (Reef fish communities are spooked by scuba surveys... PeerJ 6, e4886 (2018)).

That is one of the things we assess here: The observer effects. The effects of the presence of the observer on fish communities. Hence we do discuss it in depth. We have made this more clear in the methods section: L121-122: "The time between successive observations was at least one minute. The duration between observations was most often too short to consider observations as independent \cite{Emslie2018ReefRecover}. These dependencies between observations are described as observer effects, which will be discussed further."

L122. Why split the videos as 0-50, 5-45, 10-40, etc. and not 0-50, 0-40, 0-30m etc.?

To limit disturbances, observers would cover a transect in both directions. We have also added this in the text now. **L124-125**: "Observers covered the transects back to back, so three times in one direction and three times in the other direction." Unfortunately, it was not recorded in which direction an observer started the transect and it was very difficult to deduce this from the videos. Which means that the section 0-10 might actually have been 40-50. This is why we chose to work with the sections 5-45 and so on.

L125. Provide estimated times-distances (line 125)

Answer: this remark was not entirely clear to us.

The suggestion to include explicit analyses of species composition was also disregarded. Although indeed Diversity indices include composition, this does not tell you which taxa accounts for the differences, which is of high interest to practitioners. This is discussed briefly in the discussion for a few species, but its explicit inclusion in the analyses and presentation would add good value to the study.

Answer: We have now also added a description of the most important species affecting the fish assemblage structure differences between both islands. **L400-409**: "Given the pronounced difference of the fish assemblage structure between islands, the first PCO axis of a PCO analysis of the full data was a good proxy to distinguish the fish assemblage structures of both islands. Using the correlations of the full data with this first PCO axis, the species that are most important to explain this difference between islands, could be identified. The most important species to explain differences in fish assemblage structure between the islands included, for Santa Cruz, the Bullseye puffer fish (\textit{Sphoeroides annulatus}; \textit{r} = $-$0.62), Yellowtail damselfish (\textit{Microspathodon bairdii}; \textit{r} = $-$0.83) and Pacific spotfin mojarra (\textit{Eucinostomus dowii}; \textit{r} = $-$0.64), and, for Floreana, the Bravo clinid (\textit{Gobioclinus dendriticus}; \textit{r} = 0.56), Galapagos ringtail damselfish (\textit{Stegastes beebei}; \mbox{\textit{r} = 0.83}) and Chameleon wrasse (\textit{Halichoeres dispilus}; \textit{r} = 0.87)."

Discussion

The concluding statement has not been improved. The current text just says what is already known: "methodological parameters, environmental conditions, and ecological knowledge, e.g. species-specific traits affecting detectability, were found important for video transects and should be accounted for to properly assess the ecological state and the effect of management practices on the environment" but what exactly was discovered in the study that is new? (refer to the hypotheses and objectives).

Answer: We have adapted the conclusion accordingly and provide clear answers to the hypotheses and objectives of the study: **L535-559**: "Deciding on the distribution of the sampling effort among different methodological aspects is an important step for any study. In this study we described how researchers should take into account a broad range of potentially conflicting theoretical and practical aspects when setting up an experimental design for video transects. For most species, the presence of an observer has a significant effect on fish behavior which introduces severe dependence between subsequent observations. These observer effects seem however negligible when the overall fish assemblage structure is assessed rather than the counts of individual species. Similarly, the overall sampling variability (consisting of observer effects, instantaneous fish displacement and random counting errors) can for some individual species be very high (up to 100 \%), while for community assessments it remains relatively low (17-26\%). The errors associated with the perception of the observers themselves on the other hand, i.e. observer bias, is negligible for both community and species assessments (mostly below 1\%). Longer transects have a much lower sampling variability (33\% lower for community assessments), yet to increase precision of repeated observations, more repeats seem more effective than longer transects. For transects, the MaxCount is preferred over MinCount as the former seems to yield a lower sampling variability even though the video processing time is the same. Using counts instead of presence/absence data is also preferred, as the sampling variability of the observations of the former is somewhat lower. However, if the amount of time available for video processing is limited, presence/absence observations still provide observations of reasonably low sampling variability. In summary, sampling variability, methodological parameters, environmental conditions, and ecological

knowledge, e.g. species-specific traits affecting detectability, are important for video transects and should be accounted for to properly assess the ecological state and the effect of management practices on the environment.”

Reviewer #3:

Comments to the Author(s):

The manuscript was reviewed and I am in agreement with regard to the comments/corrections suggested by the reviewers. The manuscript was revised to address many of the comments suggested by the reviewers and the authors gave a response for each comment or question. However, some of the responses were not completely addressed or could be completely addressed without repeating the analyses or data collection. For example, counting or lumping all species together with no separation of species by behavior or life stage is an issue which cannot be addressed without reorganizing and re-analyzing the data. The authors did offer responses and added statements to try and address in the revision but the issue is not fixed.

Answer: We have added in Table G.2, a literature-based score of the reaction to divers of the different species. "The reaction-to-observer provides a literature-based score from 1, for seemingly shy and easily frightened species, to 6, for seemingly curious species \citep{Humann2003ReefGalapagos}." In the methods section we mention that "A literature-based score of reaction-to-observer was compared with the results of these models \cite{Humann2003ReefGalapagos}." And this is described in the results section as: **L326-329**: "For 9 out of 11 species with significant observer effects, the literature-based score of the reaction-to-observer corresponded with the observed fish behavior towards observers (Table G.2). There was a discrepancy in the observed effects and literature-based score for the Amarillo snapper and Yellowtail damselfish."

The following are specific comments that require further attention:

Not determining the exact length of transects may have introduced additional variability but this variability isn't measured by the authors or quantified. They suggest it can be assumed to be stochastic and limited. Can it be measured or is assuming it to be stochastic and limited adequate? It may or may not be adequate but a better justification should be given for this point and others that were questioned by the reviewers.

Answer: It is not possible to quantify the additional variability that is introduced by not exactly knowing how long the transects are, but we provided more information to support our assumptions: **L141-143**: "Although the inability to provide exact distances will introduce some additional variability that cannot be quantified, we assumed this variability to be stochastic and limited as observers were specifically trained to maintain a constant swimming speed."

As reviewer 2 pointed out, the Introduction should be written to emphasize the importance of this study in comparison to others and its contribution to the existing literature. I don't believe that the authors response to address this comment is adequate. The foundation for the methods to be use should be established in the Introduction along with clearly stated objectives.

Answer: We have rewritten the last part of the introduction. Now the aim of the study is written down more explicitly, more early on in the manuscript and within the context of existing gaps in literature. **L46-55**: "Although studies comparing different video and other visual census techniques are becoming more frequent \cite{Andradi-

Brown2016,Langlois2010,Tessier2013,Watson2005,Watson2010,Wilson2018}, few have focused on the methodological aspects of video transects, such as observer bias (Table \ref{tab:gloss}), design errors (Table \ref{tab:gloss}), sampling variability, counting metrics, data types and data transformations \cite{Bernard2013,Goetze2019}. Video transects are often considered a simple extension of visual census transects, although the ability to store and standardize video observations (see \nameref{ap:add:applic}) justifies a specific methodological assessment of the technique. The aim of this study was therefore to provide a detailed assessment of the aforementioned methodological aspects and overall suitability of video transects to study reef fish assemblages."

Both reviewers suggested making the objectives more clear in the Introduction section. The authors mentioned that the objectives are given from line 107 to 135. However, the Introduction ends with line 78. There are 3 questions listed at the end of this section but those are not objectives. If the objectives are in fact given at the end, I cannot find them. The study objectives should be stated clearly at the end of the Introduction which they are not. I also agree with reviewers that the Introduction needs to include the a description of the type of data that is important for ecologists and managers and how the study will address ways to collect and analyze the desired types of data.

Answer: We have written down the objectives as research questions. We now specify the general aim of the study and break it down in three research questions, which are introduced more clearly now in the introduction.

The choice of the data transformation was stated by the authors as being highly dependent on the research question(s) of the study. Though readers may be interested in how different transformations may affect the results of analyses, the authors didn't really address the how the data transformation addresses their questions or in other words, what the point of using different data transformation was for the conclusions of the current study. There should be more to the answer than just stating that researchers might be interested in. State the importance of determining this result to researchers in the Introduction.

Answer: We included a more detailed description of the meaning/interpretation of data transformations: **L274-279**: 'In addition, the considered transformation of abundance data to presence-absence data entails a broader discussion regarding the way data should be collected (i.e. identification and counting versus only identification respectively).' And **L591-595**: 'Although neglecting the abundance of a species scored worst in terms of goodness-of-fit, the difference between MinCount and presence-absence data was rather small. Therefore, in this specific case, if one wanted to reduce the time required for video analysis, the presence-absence instead of the abundance data could have been used, without much information being lost.' We do think however that the issue of data transformation does not fit well in the introduction as it would negatively affect the structure. If we would discuss all methodological aspects in the introduction in detail it would negatively affect the clarity of the introduction. We do, however, now also mention it explicitly in the aim of the study in the introduction.

The choice of statistics does make the manuscript rather complicated but justify why you are going to use these methods this in the Introduction. I understand what the authors were trying to do but it's not

explained completely within the manuscript so that the readers will understand what the objectives were and why specific analyses were done.

Answer: Discussing this in the introduction in detail would make the introduction very hard to follow and draw the attention away from the other aspects that need to be addressed in the introduction. We added following in the introduction to highlight the need for the specific methods and also refer the reader to the part of the manuscript at which more information is provided: **L78-82**: “The innovative, yet complex design of this study can provide new insights in the methodology of observation techniques, yet requires multiple advanced statistical methods. An overview of these methods and reasoning behind their use is provided in section \ref{Data_analysis}.”

Lastly, the following two comments given by reviewer 2 were not adequately addressed.

1. The study considers abundance and diversity of fish, but did not presented changes in species composition, which is one of the most fundamental factors affected by different survey methods. I suggest including this explicitly. The authors did not mention if they will add anything to the manuscript regarding changes in species composition. If not, why ?

Answer: We made this more clear in the text by adding: **L276-279**: ‘Finally, following the predominant terminology used in literature, the multivariate analyses of abundance and presence/absence data will yield insights in the structure and species composition of fish assemblages, respectively.’ Hence the distinction between fish assemblage structures and species composition can be brought back to the use of abundance versus presence/absence data. The repercussions of different types of data (abundance versus presence/absence) is discussed in the discussion.

2. Similarly, the conclusions should focus on the contributions of your study. I would also suggest that given the extensive nesting design and interacting factors, the interpretation and concluding remarks should be stated clearly in the discussion as a table summarizing a set of recommendations for practitioners framed within the specific context of the study (i.e. ecosystem/habitat surveyed). It would be good for example to have carried out surveys in different habitats (need to specify in the manuscript if all transects were done in the same habitat) so the findings could have a greater applicability. The authors did not add the specific recommendations to the revision nor did they give any indication if they will or will not add them and why.

Answers: it was added that all transects were laid out in rocky habitats (**L91-92**). Specific habitat characteristics were not available, but habitats were very similar as rocky habitats were targeted for placement of the transects. Instead of adding a table we have extended the conclusions with the most important results of the manuscript. Most of the recommendations need to be discussed in the context of the aims, area, etc. which would take too much space in a table. Recommendations in a table without this context would lead to potential misinterpretation and we want to avoid this.

Just a note, MaxN and MinCount are not density estimates. There is no unit of area included in their calculation. These can be considered abundance metrics but not density metrics.

Indeed. They are not being referred to as density metrics in the manuscript. All mention of fish densities have been removed and/or replaced by ‘fish abundance(s)’.
