# Supplementary material for: Sampling errors and variability in video transects for assessment of reef fish assemblage structure and diversity
Source: PLoS One. 2022 Jul 25;17(7):e0271043. doi: 10.1371/journal.pone.0271043 (PMC9312474; doi:10.1371/journal.pone.0271043)
Supplement: Attachment — Submitted filename: Rebuttal_Plos_One_Stijn_Bruneel.docx [file pone.0271043.s026.docx]

# Rebuttal letter

We thank the reviewers for their helpful comments and interest in the topic. The remarks have contributed to the outcome of the reviewed manuscript and have, in our opinion, increased the quality of its content and structure. In the next section we will clarify how we integrated the comments and suggestions in this reviewed version of the manuscript.

## Reviewer #1: Overview

This paper reports on the accuracy, precision and repeatability of visual transects of shallow water reef fish recorded with underwater video. In particular, the authors aim to address whether there were species-specific observer affects, the importance of observer bias sampling variability, and the importance of transects length number repeats the counting metrics and the way that data was transformed.

As an initial comment the authors do not recognise that the **level of transformation that you use is largely dependent on the ecological question that you are asking**.

Answer: Indeed, the choice for a transformation is largely dependent on the research question, but that does not alter the fact that it might be interesting to know how different the results would have been if different transformations would have been used. We clarified this by adding following sentence: “Although the choice for a data transformation or dissimilarity index is highly dependent on the research question, researchers might still be interested in their effect on model fit.”

I also note that they are missing some of the **fundamental literature** including previous similar work and recommend that they actually read the reference below which is fundamental to this field of study.

McCormick, M.I., Choat, J.H. Estimating total abundance of a large temperate-reef fish using visual strip-transects. Mar. Biol. 96, 469–478 (1987). <https://doi.org/10.1007/BF00397964>

Answer: We have gone through the reference and found indeed many conclusions that were re-established in more recent publications which we already cited. We now also included this reference to support some statements in the manuscript.

I found the paper **extremely long and overly complicated**. Also, while I recognise that the statistics used are valid, I am not convinced they are the **optimal methods** for conveying and supporting the authors interpretation presented in the discussion.

Answer: We agree that the manuscript might be quite complex, but we believe that it is important to discuss aspects which are often gone over quite quickly such as the correct partioning of variability among different factors, the limitations of the chosen design, etc. The choice for the different statistical techniques was not made lightly, we believe the chosen methods to be most appropriate.

I’m also concerned that the authors may have **unwittingly introduced another source of bias** into their design which may well greatly impact their analysis and data collection.

At lines 144 to 145 the authors describe laying out a transact rope and recording 2.5 m either side of the transact. Did they delineate the whole transect by laying out a rectangle or did they lay out a rope down the centre and then the estimated the distance either side of that transect by the image analyst? If they did the latter can the author’s please clearly define how they actually estimated distance. Distance estimates are 1 of the main sources of error that come into fish transects as it can be extremely difficult to consistently estimate distance. As a consequence, an observer may greatly over or underestimate the actual area observed.

Answer: Indeed, both the observers and image analysts were trained to estimate a distance of 2.5 meter on either side of the rope and to decide whether fish had to be included or not. They were trained to estimate the distance but the transects themselves had no delineation of the 2.5 meter borders. These borders had to be estimated on site. It is indeed correct that these estimates will not always be accurate. Therefore we have added following to the manuscript: “It should be noted that, despite their training, observers and image analysts might have over or underestimated the distance from the rope, inflating as such the inter-observer variability and/or sampling variability to some extent.”

See Harvey E, Fletcher D, Shortis MR, Kendrick GA. A comparison of underwater visual distance estimates made by scuba divers and a stereo-video system: implications for underwater visual census of reef fish abundance. Marine and Freshwater Research. 2004 Sep 23;55(6):573-80.

Answer: Thank you for the interesting reference that discusses the issue of distance estimates. We have included it as a reference for the earlier mentioned statement.

The authors also talk about a standard operating procedure developed by the aquatic ecology research group of Ghent University. It would be really useful if this could be deposited as an online appendix so that people could refer to that. Also, how does that align with already published standard operating procedures for doing video transects surveys of fish?

Answer: The methodology description that follows is actually the standard operating procedure we are referring to. We have removed this sentence now as it might actually be confusing for the reader.

I also believe there are some real fundamental errors with this paper in that the authors are trying to count mobile fishes that are quite large and also small fishes such as travel fins and Lenny’s which are habitat associated. Most fish ecologists would use transects of different sizes depending on the mobility and size of the species that they are trying to investigate. Trying to sample them all in one survey is probably not realistic or ecologically valid. I certainly would not be trying to survey fish such as the Galapagos triplefin blenny (max size 9cm and highly territorial) in the same transects that I would be targeting the Amarillo snapper (a species up to 71 cm in length and highly mobile).

Answer: Our main aim is to get an idea of the fish assemblage structure with as little effort as possible. Including more transects of different sizes to assess different species would indeed be more correct, but would also require much more time in the field. Here we want to optimize video transects to assess fish assemblage structures and compare them among sites in a relatively short time.

It would be great if the authors can make some comment about why they have lumped the species together when most guidance on surveying reef fish would recommend keeping them separate.

Answer: We added following to the text to clarify our choice: “Because we aimed to provide a quick and mobile assessment of the local fish assemblages and limit the sampling effort per site, it was decided to count all fish species \citep{Bernard2013,Watson2010} and not to count discrete groups of species in transects of different, earlier established, optimal designs \citep{McClanahan2007,Wilson2018}.”

Overall I think the paper has the potential to be a valid contribution to science, but it needs to be substantially rewritten so that the text and the data presented a simplified. At the moment it is overly and unnecessarily complex. I recommend simplifying the analysis and the presentation of results so that readers can comprehend it and absorb the main takeaway messages. I have included a number of minor comments below.

Answer: We went over the manuscript and simplified many of the sentences and representations of the results.

Abstract

Line 6. I recommend that you change should to could given that this work (admittedly not for video sampling) is a repeat of past research.

Answer: done

Introduction

Line 56. I recommend that you write this as In the literature.

Answer: done

Line 62. I recommend that the authors put a reference about underwater visual census and some species being scared away and not been detected.

Answer: Three references were added

Lines 59-67. I commend the authors for this distinction which is actually really important.

Answer: Thank you

Line 78 to 80. This statement is actually incorrect. Because of the bait plume from stationary cameras (and I am assuming you are talking about baited remote underwater video systems given you refer to Mike Cappo’s research above) have been demonstrated to be a more effective tool sampling across broad habitats and depth scales than underwater visual census. They tend to sample more species, with greater statistical power than underwater visual census. However, the bias of baited remote underwater video systems are acknowledged and one of the things that you cannot do is get density or biomass estimates per unit area.

Answer: Indeed. The use of baited stationary devices remediates some of the limitations of traditional non-baited stationary devices. We have rewritten this part: Nevertheless, stationary cameras only provide a limited view of the study area as only small patches are monitored, and are therefore of limited use when an extensive survey of a study area has to be done \citep{Willis2000}. Baited stationary cameras remediate this limitation to some extent by attracting nearby fish, providing a more representative assessment of the local fish assemblages \citep{Harvey2007}. However, due to its point-based nature, it remains difficult to accurately estimate fish densities and account for habitat heterogeneity \citep{Andradi-Brown2016,Willis2000}. Because fish species, and in particular reef fish, are often strongly associated with specific micro-habitats \citep{Chapman2000}, a more mobile approach may often be more appropriate for more cost-effective monitoring of fish densities.

They are also not good for getting counts of some of the smaller and less mobile species. I think you probably want to either reword this sentence or alter it to reflect a true statement.

Similarly, I think in the introduction you need to talk about the types of information that ecologists and managers want to collect and use. You have not talked all about the use of length data which some ecologists would consider to be more fundamentally useful than count data. This aspect should probably be brought into the discussion as well.

Answer: In the introduction we delineate the objective of the manuscript and discuss the value of obtaining insight in the structure and diversity of fish assemblages. Length measurements can indeed be very useful for specific ecological questions, but we don’t believe that mentioning this here would provide much added value, especially since we have been trying to reduce the length of the manuscript.

Can you provide a reference for line 87 please.

Answer: We provided two references.

Lines 171-174. Can the authors please justify why they have used Max count or min count? One of the great advantages of transects is that you can actually obtain a density estimate. By counting all the fish that you see with in a transact you may increase your variability, but you will also increase data on length frequency. This is a fundamental advantage of doing transects over stationary camera systems. Max count is a term that comes from the baited underwater video literature and is probably inappropriate in this context. Essentially all you are doing is counting all the species and individuals that you see in a transect? I would clarify that and change your figures accordingly.

Answer: We have clarified why we determined both metrics: Fish were counted in such a way that both MaxCount (total number of individuals per species) and MinCount (maximum number of individuals per species in one frame, also referred to as MaxN) could be determined (see section \ref{Video_analysis}). Since MaxCount was found most appropriate for our video transects (see section \ref{Variabilityinthestructureoffishassemblages}), which is in line with results from previous studies on video transects \citep{Mallet2014,Wartenberg2015}, it was used instead of MinCount.

Somewhere in your methods you need to tell the reader how you analysed your video imagery and why used that particular method. At the moment, someone wanting to repeat your work couldn’t do it as that information is not in the main text or the appendices.

Answer: This information is provided in Appendix B. We have expanded the description of the method and included also why it was done as such.

Line 179 to 181. Please make a comment about what the effect of not being able to identify the exact length of the transects might have on the data. It seems like this is probably going to increase a source of error and variability as you may be greatly increasing or decreasing the area surveyed.

Answer: We have clarified what the effect might be of not being able to get exact distances: “Although the inability to provide exact distances will introduce some additional variability, this variability is assumed to be stochastic and limited.”

Line 190. Do you have an example or a reference supporting your thoughts about instantaneous fish displacement? Has anyone else observed this in the scientific literature?

Answer: The number of studies to repeat transects at relatively short time intervals is limited. In most studies transects are not repeated and hence this issue does not occur. However, these studies are also not able to identify the different origins of variability. There are advantages and disadvantages to both approaches, but given our research questions, we believed this approach to be most appropriate. The few studies that do repeat transects don’t go into much detail when describing the impact this might have on the data analysis. We believe this however an important aspect to include. We see this as a gap in current literature as we have not found any suitable references to support this message. A disadvantage of describing this in depth might however be that the text becomes more complex, but we believe it necessary to be included in order to be scientifically sound.

Figure 3. In the methods I think it would be really useful of the authors stated why they have used the similarity values to represent their transects and not looked at the cumulative number of species or individuals and also presented the means. The dissimilarity values are a valid way of presenting this, but an unusual way of presenting this data. I’m not convinced that this the optimal way of presenting it.

Answer: First, in this manuscript we mainly focussed on the ability of video transects to be used for assessments of fish assemblage structure. We also included assessments of diversity and in the appendix we included some species accumulation curves for different transect lengths which are briefly referred to in the discussion to support a statement. However the focus is on fish assemblage structure. Second, this design error we are interested in is defined in the methods section as: “An important design error may arise from the choice of transect length. If transect lengths are too short, many observations may contain no species at all and there may also be pairs of observations having no or only few species in common, leading to uninformative dissimilarity values and questionable multivariate analyses \citep{Clarke2006}”. We therefore clearly define this design error within the context of multivariate analyses of fish assemblage structure, justifying the use of frequencies of dissimilarity values.

Lines 429 - 442. Rather than using the components of variation have the authors considered using a distance based linear model which might be easier for the reader to understand?

Answer: Given that all the variables that we considered were categorical, a PERMANOVA was found more suitable and more straightforward to interpret than a DISTLM (Anderson et al., 2008).

Line 464. Please change the use of species density as if you are using min count or Max count you’re not counting all the individuals of a species. Again, you have used Max count inappropriately.

Answer: We define MaxCount as the total number of individuals per species, which means that all fish are counted. Species density is the number of species per unit of surface. That would be the same for MinCount or MaxCount. Only MaxCount is used throughout the analyses.

Line 533 - 534. Please note that both the species that you list as pelagic curious species are actually reef associated and benthic. They are not pelagic.

Answer: We corrected this.

## Reviewer #2: Overview

This study is interesting and could be a valuable contribution in the branch of fish ecology at local and regional scale; however, its contribution is limited in terms of added improvement in the field of methodologies to survey the structure of fish communities, and the manuscript must be written with this in consideration. The study of sources of variation is extensive in fish sampling and most of the elements analyzed here are well known within rules of thumb of sampling design and data capture:

1. Fish communities change in space and time, and this should be considered in sampling designs. 2. Different sampling efforts (number, size, or duration of sampling units) will render different results, the more the better, but the optimum depends on how diverse the community under study is. 3. Different methods of capturing information (video vs visual, static vs transects, camera vs diver) have specific advantages and disadvantages for the capture of abundance and diversity of fish, which need to be considered according to the habitat and question at hand. 4. Divers have an effect on the fish community and different divers will collect different information, and this will depend on their experience in great part, so surveyors need to have an adequate level of knowledge to be surveyors, again, according to the complexity of the system under study.

The study focused on a particular method of diver-based roaming video transects in a nested design with repeated-measures and its results confirm most of these rules of thumb. So, what exactly is this study addressing that has not been done before to make this study unique from previous studies? I believe the key point of the study is that previous studies focused on only a few aspects of the sources of variability while the authors of this paper carried out a more complete inclusion of these by carrying a complex sampling design. However, the benefits of doing this should be well explained. For this the study needs to clearly outline the work that has been done previously regarding sources of bias and error in fish communities’ assessments to clearly identify the knowledge gap that will be addressed. Objectives, questions and/or hypothesis of the study need to be clearly outlined in abstract and introduction, including independent variables (elements of sampling and analyses) and dependent variables (elements of community structure).

Answer: We believe that the motivation for this specific design has been described in the introduction from line 107 to line 135. Regarding the objectives, these are given from line 136 to 140 at the end of the introduction. We did not want to go too much into the methodological aspects of the objectives at this point because we hadn’t formulated the aspects of the sampling design and technique yet. This would be of little added value for the reader and might even be confusing.

I have also made several comments in the manuscript itself. but here I stress some general things. The language and terms used to refer to sampling variability must be clearly defined within the discipline of ecology. The use of terms such as design errors, detection heterogeneity, observer bias, observer effects, random counting and instantaneous fish displacement sounds all confusing, but I think it all can be explained more simply to avoid confusions. There are some statements in the introduction that are not accurate or sufficiently backed up with other studies regarding the use or adequacy of different survey methods.

Answer: In this manuscript we have relied on the terminology and definitions used in literature. We provided some additional clarifications for some terms throughout the text.

In the abstract only the most essential information can be presented. Therefore, we could not elaborate on all the aspects and results of the study in the abstract.

Comments in the pdf we have answered within the pdf: PONE-D-21-06060_errors and variability in video transects_SB.pdf

The study considers abundance and diversity of fish, but did not presented changes in species composition, which is one of the most fundamental factors affected by different survey methods. I suggest including this explicitly.

Answer: We provide assessments of the fish assemblage structure which takes into account both species composition and abundance. We also compared the repercussions of choosing presence/absence data versus abundance data, providing an additional assessment of species composition without considering abundance.

The discussion needs to avoid repeating results and just aiming to interpret the findings and its significance concisely.

Answer: We have improved the brevity of sentences in the discussion by focussing more on the interpretations and less on the results.

Similarly, the conclusions should focus on the contributions of your study. I would also suggest that given the extensive nesting design and interacting factors, the interpretation and concluding remarks should be stated clearly in the discussion as a table summarizing a set of recommendations for practitioners framed within the specific context of the study (i.e. ecosystem/habitat surveyed). It would be good for example to have carried out surveys in different habitats (need to specify in the manuscript if all transects were done in the same habitat) so the findings could have a greater applicability.

Answer: We prefer to keep these recommendations in the discussion as it allows to provide more context. If we would do this in the conclusion, the conclusion would be too long and not of much added value.

## References

Anderson, M.J., Gorley, R.N., Clarke, K.R., 2008. PERMANOVA+ for PRIMER: Guide to Software and Statistical Methods, PRIMER-E Ltd., Plymouth, UK.
