# Supplementary material for: Sampling errors and variability in video transects for assessment of reef fish assemblage structure and diversity
Source: PLoS One. 2022 Jul 25;17(7):e0271043. doi: 10.1371/journal.pone.0271043 (PMC9312474; doi:10.1371/journal.pone.0271043)
Supplement: Attachment — Submitted filename: PONE-D-21-06060R1_ManuscriptReview_15Nov2021.docx [file pone.0271043.s027.docx]

**Review of revised manuscript**

Title: Sampling errors and variability in video transects for assessment of reef fish assemblage structure and diversity.

**Comments to the Author(s):**

The manuscript was reviewed and I am in agreement with regard to the comments/corrections suggested by the reviewers. The manuscript was revised to address many of the comments suggested by the reviewers and the authors gave a response for each comment or question. However, some of the responses were not completely addressed or could be completely addressed without repeating the analyses or data collection. For example, counting or lumping all species together with no separation of species by behavior or life stage is an issue which cannot be addressed without reorganizing and re-analyzing the data. The authors did offer responses and added statements to try and address in the revision but the issue is not fixed.

**The following are specific comments that require further attention:**

Not determining the exact length of transects may have introduced additional variability but this variability isn't measured by the authors or quantified. They suggest it can be assumed to be stochastic and limited. Can it be measured or is assuming it to be stochastic and limited adequate? It may or may not be adequate but a better justification should be given for this point and others that were questioned by the reviewers.

As reviewer 2 pointed out, the Introduction should be written to emphasize the importance of this study in comparison to others and its contribution to the existing literature. I don't believe that the authors response to address this comment is adequate. The foundation for the methods to be use should be established in the Introduction along with clearly stated objectives.

Both reviewers suggested making the objectives more clear in the Introduction section. The authors mentioned that the objectives are given from line 107 to 135. However, the Introduction ends with line 78. There are 3 questions listed at the end of this section but those are not objectives. If the objectives are in fact given at the end, I cannot find them. The study objectives should be stated clearly at the end of the Introduction which they are not. I also agree with reviewers that the Introduction needs to include the a description of the type of data that is important for ecologists and managers and how the study will address ways to collect and analyze the desired types of data.

The choice of the data transformation was stated by the authors as being highly dependent on the research question(s) of the study. Though readers may be interested in how different transformations may affect the results of analyses, the authors didn't really address the how the data transformation addresses their questions or in other words, what the point of using different data transformation was for the conclusions of the current study. There should be more to the answer than just stating that researchers might be interested in. State the importance of determining this result to researchers in the Introduction.

The choice of statistics does make the manuscript rather complicated but justify why you are going to used these methods this in the Introduction. I understand what the authors were trying to do but its not explained completely within the manuscript so that the readers will understand what the objectives were and why specific analyses were done.

Lastly, the following two comments given by reviewer 2 were not adequately addressed.

**1.** The study considers abundance and diversity of fish, but did not presented changes in species

composition, which is one of the most fundamental factors affected by different survey methods. I

suggest including this explicitly.

The authors did not mention if they will add anything to the manuscript regarding changes in species composition. If not, why ?

**2.** Similarly, the conclusions should focus on the contributions of your study. I would also suggest that

given the extensive nesting design and interacting factors, the interpretation and concluding remarks

should be stated clearly in the discussion as a table summarizing a set of recommendations for

practitioners framed within the specific context of the study (i.e. ecosystem/habitat surveyed). It

would be good for example to have carried out surveys in different habitats (need to specify in the

manuscript if all transects were done in the same habitat) so the findings could have a greater

applicability.

The authors did not add the specific recommendations to the revision nor did they give any indication if they will or will not add them and why.

Just a note, MaxN and MinCount are not density estimates. There is no unit of area included in their calculation. These can be considered abundance metrics but not density metrics.

**The following are specific comment/corrections for the text:**

**Abstract:**

Reviewer #1 recommended changing "should" in line 6 which was done by the authors.

**Introduction:**

Line 5:

What environmental and ecological changes are referred to here? The prior sentence doesn't quite reference changes just physical and chemical water conditions that are known to be affected by anthropogenic pressures. What are "these changes?" Remove "these" from line 4.

Line 34:

Remove the comma and add "thereby" before "providing a more...."

Line 35:

"Baited stationary cameras" is written in the prior sentence but the authors refer to the camera systems as "its" in line 35. Replace "its" with something like "However, due to the point-based nature of these camera systems, it remains difficult....."

Line 74 to 78: The questions seem to just be listed here with little connection to the paragraph above. Why not change the wording to "Although repetitions of transects are not common in the literature, they were necessary here to provide answers to the following research questions, which are of interest for both non-repetitive and repetitive studies:"

**Discussion:**

Line 443: Remove "two curious species." This statement makes the assumption that the fish were in fact curious which is something that cannot be proven.

Line 446: Replace curious with "non-scared" fish. Being attracted to the divers doesn't mean the fish are actually curious.

Line 462 to line 469: Could the temporal auto-correlation, fish displacement, and observer biases be avoided or greatly reduced by sampling transects over a series of days not one after another on the same day? The sampling events would still be repeats.
